# Long-term and transgenerational phenotypic, transcriptional and metabolic effects in rabbit males born following vitrified embryo transfer

**DOI:** 10.1038/s41598-020-68195-9

**Published:** 2020-07-09

**Authors:** Ximo Garcia-Dominguez, Francisco Marco-Jiménez, David S. Peñaranda, Gianfranco Diretto, Víctor García-Carpintero, Joaquín Cañizares, José S. Vicente

**Affiliations:** 1 Laboratory of Biotechnology of Reproduction, Institute for Animal Science and Technology (ICTA), Universitat Politècnica de València, 46022 Valencia, Spain; 2 National Agency for New Technologies, Energy and Sustainable Economic Development (ENEA), Casaccia Research Centre, 00123 Rome, Italy; 3 Institute for the Conservation and Breeding of Agricultural Biodiversity (COMAV-UPV), Universitat Politècnica de València, 46022 Valencia, Spain

**Keywords:** Embryology, Reproductive biology, Animal physiology

## Abstract

The advent of assisted reproductive technologies (ART) in mammals involved an extraordinary change in the environment where the beginning of a new organism takes place. Under in vitro conditions, in which ART is currently being performed, it likely fails to mimic optimal in vivo conditions. This suboptimal environment could mediate in the natural developmental trajectory of the embryo, inducing lasting effects until later life stages that may be inherited by subsequent generations (transgenerational effects). Therefore, we evaluated the potential transgenerational effects of embryo exposure to the cryopreservation-transfer procedure in a rabbit model on the offspring phenotype, molecular physiology of the liver (transcriptome and metabolome) and reproductive performance during three generations (F1, F2 and F3). The results showed that, compared to naturally-conceived animals (NC group), progeny generated after embryo exposure to the cryopreservation-transfer procedure (VT group) exhibited lower body growth, which incurred lower adult body weight in the F1 (direct effects), F2 (intergenerational effects) and F3 (transgenerational effects) generations. Furthermore, VT animals showed intergenerational effects on heart weight and transgenerational effects on liver weight. The RNA-seq data of liver tissue revealed 642 differentially expressed transcripts (DETs) in VT animals from the F1 generation. Of those, 133 were inherited from the F2 and 120 from the F3 generation. Accordingly, 151, 190 and 159 differentially accumulated metabolites (DAMs) were detected from the F1, F2 and F3, respectively. Moreover, targeted metabolomics analysis demonstrated that transgenerational effects were mostly presented in the non-polar fraction. Functional analysis of molecular data suggests weakened zinc and fatty acid metabolism across the generations, associated with alterations in a complex molecular network affecting global hepatic metabolism that could be associated with the phenotype of VT animals. However, these VT animals showed proper reproductive performance, which verified a functional health status. In conclusion, our results establish the long-term transgenerational effects following a vitrified embryo transfer procedure. We showed that the VT phenotype could be the result of the manifestation of embryonic developmental plasticity in response to the stressful conditions during ART procedures.

## Introduction

Since the birth of the first test-tube baby in 1978, the use of assisted reproductive technologies (ART) has increased notably. Globally, ART use has doubled over the last decade, with a progressive rise in banking cycles in which all embryos are frozen for future ART cycles^1^. However, ART fails to mimic the optimal in vivo conditions due to the lack of knowledge regarding the dynamic regulation across the maternal womb^2,3^. Nowadays, it is well accepted that in responding to environmental cues the embryo demonstrates a high degree of developmental plasticity, modulating its metabolism, gene expression and phenotype^4^. But although this embryo adaptation is thought to increase short-term survival in suboptimal environments during ART, several studies have associated this embryo developmental deviation with adverse consequences later in life (Developmental Origins of Health and Disease theory), as has been extensively described in humans^5–11^. Furthermore, evidence of similar effects has been reported in animal studies, which avoid confounding factors related to parent infertility or other conditions such as lifestyle that further complicate interpretation of the data^5,11–15^.

However, it has been hypothesised that under extreme conditions, embryo reprogramming is likely to be the result of a direct perturbation of the process, rather than an embryo adaptation to suboptimal conditions. The underlying assumption is that depending on the nature of the in vitro manipulation, the embryo could be differentially impacted, leading to different phenotype outcomes in later life stages^4,5,11–15^. Embryo cryopreservation requires embryo exposure to an environment with toxic chemical agents and extremely low non-physiological temperatures, in which they have no intrinsic ability to survive^16^. So, it has been shown that some developmental changes could emerge following vitrified embryo transfer in the foetus and postnatal life^17–24^. In this context, we have established that each technique required in a cryopreservation-transfer procedure (i.e. embryo vitrification-warming and embryo transfer) produces an additive effect per se over the short- and long-term offspring development^24^. In particular, we also showed that the choice of the vitrification device is not a trivial decision, as different cooling-warming rates induced specific developmental responses^24^. However, in vitro embryo handling and transfer are fundamental techniques of the clinical operation in an embryo cryopreservation-transfer procedure (CTP)^7,11,25^. Then, when different stressors are present, these can act synergistically, inducing more negative effects^13^. To cover this possibility, we recently described a study where the offspring born from cryopreserved-transferred embryos were compared with those conceived naturally^26^. This study was the first demonstrating that CTP incur long-term phenotypic consequences correlated with molecular signatures. In the era of “omic” technologies, several studies are trying to elucidate the molecular mechanisms whereby these phenotypic and physiologic changes occur after embryo manipulation^14,15^. Epigenetic alterations, derived from a disturbed embryo reprogramming due to in vitro suboptimal conditions, have been proposed as causes of some long-term and heritable ART effects^5,27–30^.

Historically, fertility researchers have been trying to improve the success of ART based on the birth rate increase, but only a few are trying to discern whether ART leaves a subtle legacy in the offspring^31^. Thus, limited knowledge is available on the long-term effects of ART, and the studies assessing its heritability are almost non-existent. Therefore, to our best knowledge, there is a lack of knowledge about how an embryo CTP may change the offspring features, and how these effects can be transmitted through the germline and persevere in subsequent generations. Taking all these data into account, the aim in this work is to determine whether embryo exposure to a CTP can alter offspring features and molecular signatures across three generations (transgenerational effects).

## Results

### Establishment of the two experimental progenies throughout F1, F2 and F3 generations

**Figure 1:**
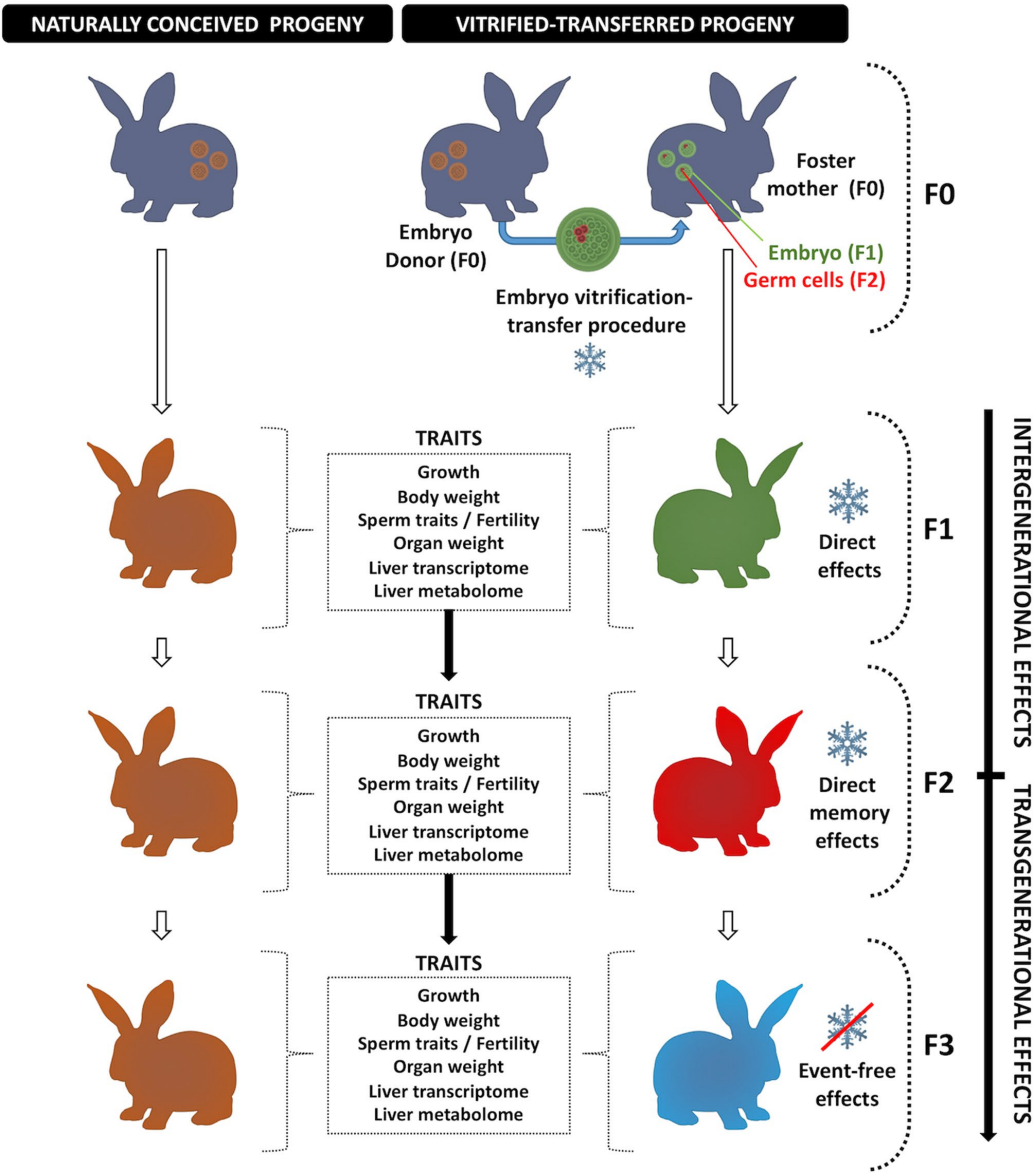
Experimental design. The experimental progenies were compared in each generation to assess the transgenerational effects of embryo vitrification procedure on body weight across the rabbit development. At adulthood, the seminal and fertility traits were evaluated. After that, animals were euthanised and the organs were weighed. Then, liver samples were collected to perform a molecular (transcriptomic and metabolomic) study.

After the transfer of 158 vitrified-thawed embryos of 13 donors into 13 foster mothers (100% pregnancy rate), 69 animals were generated (vitrified-transferred group; VT). In addition, 77 animals were generated from 14 pregnant females without embryo manipulation (naturally-conceived group; NC). The progeny produced by these methodologies (F1) were mated over two subsequent generations without embryo manipulations and respecting each experimental group (VT, NC), producing F2 and F3 animals (Fig. 1). Mating between individuals with common grandparents was avoided. Thus, 56 VT and 61 NC animals constituted the F2 generation, and 64 VT and 61 NC formed the F3 generation. There were no differences in litter size between VT and NC animals in each generation (Table 1). Thus, embryo cryopreservation-transfer procedure (CTP) did not affect the gross reproductive performance of VT progeny, compared to animals that generated without embryo manipulations (NC). Descriptive data and statistical details were annotated in Supplementary Tables S1 and S2.

**Table 1:** Efficiency in the establishment of the naturally conceived and vitrified-transferred progenies across three generations (F1, F2, F3).

Experimental groups	F1 generation				F2 generation				F3 generation			
	Founding parities	Litter size	Live births	Adult males	F1 parities	Litter size	Live births	Adult males	F2 parities	Litter size	Live births	Adult males
Naturally conceived	14	5.5 ± 0.62	77	35	10	6.1 ± 0.76	61	13	10	6.1 ± 0.89	61	24
Vitrified-transferred	13	5.3 ± 0.64	69	30	10	5.6 ± 0.76	56	9	10	6.4 ± 0.89	64	24

In F1 generation, naturally-conceived animals were generated from 14 pregnant females following natural conception, without embryo manipulation. Instead, vitrified-transferred animals were generated from vitrified-warmed embryos transferred in 13 foster mothers. F2 and F3 generations were produced selecting randomly one mature female and male from each parity of the previous generation. To reduce the inbreeding, mating between animals with common grandparents was avoided. Thereby, 10 F1 and 10 F2 parities generated F2 and F3 population, respectively.

### Growth performance, body weight and organ phenotype study

**Figure 2:**
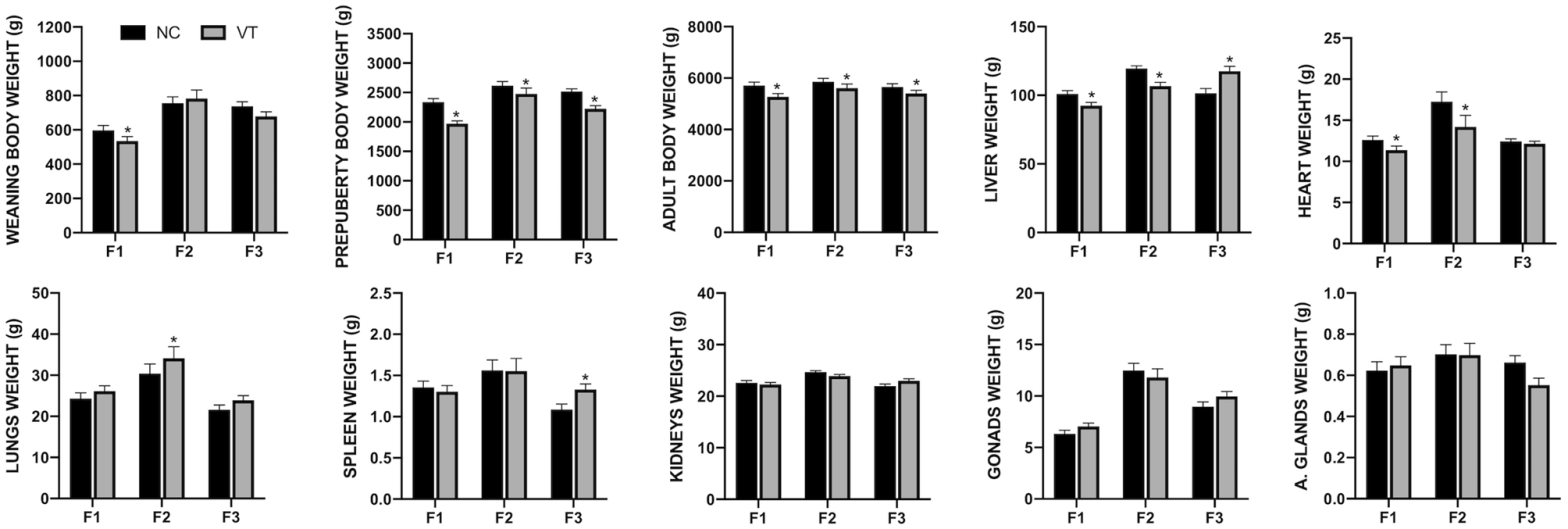
Differences in phenotypic traits between naturally-conceived (NC) and vitrified-transferred (VT) progenies during three generations (F1, F2, F3). Asterisks denote relevant differences between VT animals and their NC counterparts (Bayesian inference: |D_NC-VT_|> R and P_0_ > 0.8; Supplementary Table S2).

Comparing the growth between VT and NC animals, we noticed that VT progeny exhibited an apparent reduction in growth velocity (− 8.6 ± 1.34 g/day in F1, − 4.7 ± 2.31 g/day in F2 and − 6.6 ± 1.39 g/day in F3). This fact demonstrated not only a direct effect (F1) of the CTP but also an intergenerational (F2) and transgenerational (F3) effect of the technique. As expected, the higher effect of the CTP over the growth velocity was observed in F1, where VT animals already showed a reduced body weight at the time of weaning (− 62.2 ± 37.82 g, Fig. 2). However, in prepuberty, the reduction in the body weight was patent in VT animals of all three generations (− 370.1 ± 77.4 g in F1, − 139.3 ± 122.76 g in F2 and − 287.9 ± 70.67 g in F3) compared to the NC group (Fig. 2). This growth trend was maintained until adulthood, where VT animals showed a lower body weight (− 437.4 ± 153.43 g in F1, − 249.5 ± 209.20 g in F2 and − 247.9 ± 194.91 g in F3) in comparison to the NC group (Fig. 2). Therefore, the long-term effects of CTP had the highest impact on the F1 generation, whereas approximately the half of the effect was observed in F2 and F3.

Moreover, the VT group accumulated high probabilities (P_0_ ≥ 0.80) of phenotypic (weight) changes in some organs during the study, whose variation pattern depends on the organ and generation. However, statistical relevant differences were observed only in the liver and heart (Fig. 2). The liver showed the highest probability of change throughout generations (P_0_ = 0.99 in F1, P_0_ = 1.00 in F2, and P_0_ = 1.00 in F3), even after data were corrected for body weight. VT progeny exhibited lower liver weight (− 9.3 ± 3.56 g) in F1, which was aggravated (− 12.7 ± 3.54 g) in F2 animals. However, this trend was inverted in the F3 generation (16.1 ± 4.99 g). Attending to the heart phenotype, VT animals showed a reduced organ weight in the F1 (− 1.5 ± 0.73 g), also aggravated in F2 (− 3.1 ± 1.87 g). However, no differences in the heart weight were observed in F3 between VT and NC animals. As specific cases, increased weight was observed in F2 for the lungs (3.7 ± 3.75 g) and in F3 for the spleen (0.2 ± 0.10 g). Descriptive data of the phenotypic traits were annotated in Supplementary Table S1, and the statistical details were annotated in Supplementary Table S2.

### Sperm and fertility rate assessment

Even though no differences in the size of the gonads were observed in any generation, higher ejaculate volumes were produced by VT animals from the F1 and F2 generation. However, an increment in sperm production (TSE) was only observed in F2-VT animals, which also showed lower abnormal sperm percentage (ABN) than their NC counterparts. Attending to the motion parameters, F1-VT animals produced sperm with higher curvilinear velocity (VCL), straight-line velocity (VSL) and average path velocity (VAP). Meanwhile, F3-VT animals showed lower VCL. Results of seminal parameters were annotated in Table 2.

**Table 2:** Ejaculates/sperm parameters and motility assessment of males from the vitrified-transferred progeny (VT) compared to those naturally conceived (NC).

Generation	F1		F2		F3	
Experimental group	NC	VT	NC	VT	NC	VT
**N**	*76*	*173*	*72*	*62*	*120*	*135*
**Semen parameters**						
VOL (mL)	0.69 ± 0.050	0.89 ± 0.033*	0.60 ± 0.033	0.80 ± 0.036*	0.51 ± 0.0211	0.44 ± 0.0202
CON (10^6^)	238.5 ± 15.69	201.7 ± 10.40	181.5 ± 8.45	172.5 ± 9.11	305.6 ± 17.56	307.5 ± 16.79
TSE (10^6^ spz)	156.8 ± 15.70	179.1 ± 10.40	92.1 ± 6.92	137.0 ± 7.31*	145.8 ± 8.15	126.5 ± 7.57
MOT (%)	71.9 ± 1.93	76.9 ± 1.28	90.0 ± 0.67	88.5 ± 0.65	52.1 ± 2.61	53.7 ± 2.46
PRO (%)	40.5 ± 1.73	43.0 ± 1.16	55.9 ± 1.30	59.8 ± 1.42	27.5 ± 1.74	29.5 ± 1.64
VIA (%)	74.5 ± 1.13	74.1 ± 0.77	82.6 ± 0.69	83.0 ± 0.75	73.1 ± 1.22	73.2 ± 1.15
NAR (%)	90.3 ± 0.83	90.3 ± 0.55	88.4 ± 0.55	88.2 ± 0.61	92.9 ± 0.56	92.3 ± 0.53
ABN (%)	19.6 ± 0.98	20.7 ± 0.64	18.4 ± 0.60	14.9 ± 0.66*	22.8 ± 0.97	23.1 ± 0.92
**Motion parameters**						
VCL (μm s^−1^)	86.6 ± 2.34	99.8 ± 1.55*	99.8 ± 1.44	96.2 ± 1.53	104.5 ± 2.17	96.4 ± 2.05*
VSL (μm s^−1^)	34.0 ± 1.47	40.4 ± 0.98*	55.9 ± 1.18	55.0 ± 1.28	38.8 ± 1.22	38.3 ± 1.15
VAP (μm s^−1^)	50.0 ± 1.87	59.9 ± 1.23*	74.1 ± 1.31	72.9 ± 1.42	58.3 ± 1.25	55.8 ± 1.40
LIN (%)	40.3 ± 2.15	42.0 ± 1.42	58.7 ± 0.94	57.0 ± 0.98	37.9 ± 1.03	39.9 ± 0.97
STR (%)	68.9 ± 1.07	67.2 ± 0.71	77.1 ± 0.44	75.4 ± 0.46	66.1 ± 0.94	67.6 ± 0.88
WOB (%)	57.2 ± 1.32	59.3 ± 0.87	74.9 ± 0.99	76.0 ± 1.05	56.3 ± 0.95	58.1 ± 0.89
ALH (μm)	2.7 ± 0.55	2.8 ± 0.37	2.7 ± 0.42	2.7 ± 0.44	3.2 ± 0.06	3.0 ± 0.06
BCF (Hz)	10.8 ± 0.18	11.2 ± 0.12	9.9 ± 0.20	9.8 ± 0.22	12.2 ± 0.20	11.8 ± 0.19

Data are expressed as mean ± standard error of means.

*n* number of ejaculates, *VOL* ejaculate volume, *CON* spermatic concentration, *TSE* total sperm per ejaculate, *spz* spermatozoa, *MOT* percentage of sperm motility, *PRO* percentage of progressive motility, VIA percentage of viable sperm, *NAR* percentage of normal apical ridge, *ABN* percentage of abnormal forms, *VCL* curvilinear velocity, *VSL* straight-line velocity, *VAP* average path velocity, *LIN* linearity coefficient (VSL/VCL × 100), *STR* straightness coefficient, *WOB* wobble coefficient (VSL/VAP × 100), *ALH* amplitude of lateral head displacement, *BCF* beat cross-frequency.

*Asterisks denote relevant differences between VT traits and their NC counterparts (Bayesian inference: |D_NC-VT_|> R and P_0_ > 0.8; Supplementary Table S3).

Nevertheless, unequivocally, these variations were biologically irrelevant, as no differences were observed in the fertility rate (44.1 ± 1.41%) in the three generations. Since no experimental group nor generation effect was detected, data were considered together. Besides, we found that CTP causes a consistent increase in the number of liveborn in the three generations (6.4 ± 0.30 VT vs 4.9 ± 0.22 NC). No effect of generation was detected, so data were considered together for the experimental groups. Descriptive data of the seminal traits were annotated in Supplementary Table S1, and the statistical details were annotated in Supplementary Table S3.

### Comparative study of liver transcriptome

**Figure 3:**
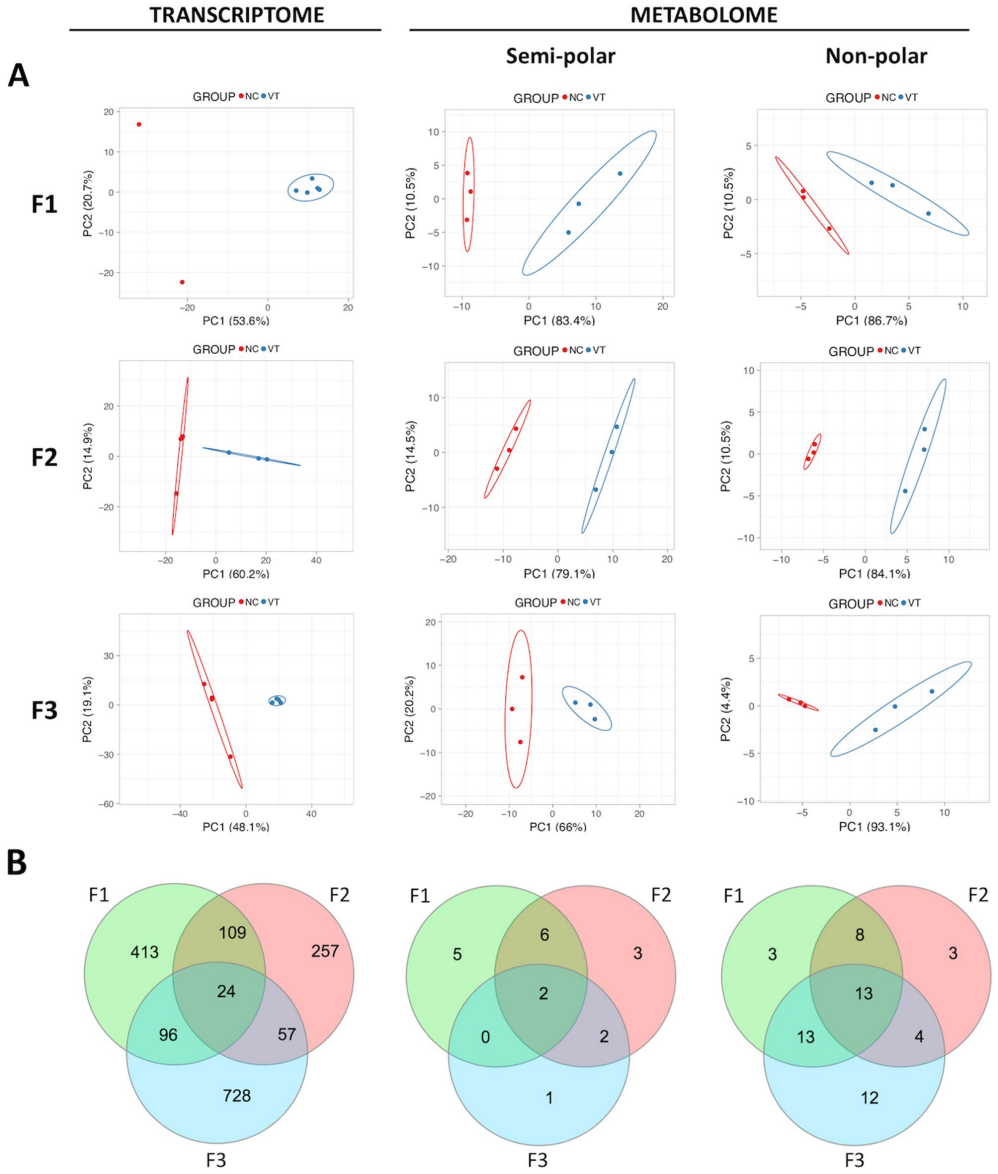
Molecular analysis of the liver samples collected from adult males derived from vitrified-transferred embryos (VT) and naturally-conceived (NC), which was compared in each generation (F1, F2, F3). (**A**) Principal Component Analysis (PCA) of the transcriptome, semi-polar metabolome and non-polar metabolome. The representation of sample variability between the experimental groups was performed taking into account only the differentially expressed transcripts or differentially accumulated metabolites. (**B**) Venn diagram summarising differentially expressed transcripts and targeted metabolites between NC and VT progenies in F1, F2, F3, and those commonly present between generations.

In each generation, the transcriptome profiling of adult liver tissue was compared between VT and NC animals. The mean number of raw reads was 65.1 ± 23.78 (± SD) millions, and in each sample, transcripts from 13,313 to 14,414 different genes (from a total of 24,964 annotated transcripts of Orycun2.0) were detected. Principal Component Analysis (PCA) clustered the samples according to their origin (VT or NC) in the three generations (Fig. 3A). In the F1 generation, the comparative transcriptomic analysis recorded a differential expression of 642 genes, of which we observed a higher number of downregulated (477 [74.3%]) genes in VT animals compared to NC group. From these differentially expressed transcripts (DETs), DAVID software recognised 518 (Supplementary Table S4). After the GO enrichment analysis, results showed that 21 biological processes (BP), 16 cellular components (CC) and 9 molecular functions (MF) were significantly affected by the CTP. Besides, KEGG analyses revealed 24 disturbed pathways. Functional annotation of F1 DETs was described in Supplementary Table S5. Most importantly, as showed by Venn diagram (Fig. 3B), of the total DETs annotated in the F1 generation, 133 and 120 DETs were inherited by the F2 and F3 generation, respectively (Supplementary Table S6). Functional annotation of these DETs were described in Supplementary Tables S7 and S8. On the other hand, comparing the VT and NC progeny in the F2 generation, 447 DETs were recorded, of which we observed a higher number of upregulated (261 [58.4%]) genes in VT samples compared to the NC group. From these DETs, DAVID recognised 342 (Supplementary Table S9), whose functional analysis revealed changes in 14 BP, 4 CC, 7 MF and 16 KEGG pathways (Supplementary Table S10). Finally, comparing the VT and NC progeny in the F3 generation, 905 DETs were recorded, of which we observed a higher number of downregulated (749 [82.8%]) genes in VT samples compared to the NC group. From these DETs, DAVID recognised 670 (Supplementary Table S11), whose functional analysis revealed changes in 29 BP, 10 CC, 13 MF and 37 KEGG pathways (Supplementary Table S12).

Generally, analysis of DETs was conducted comparing VT progenies with their coetaneous NC counterparts in each generation. However, the subset of DETs inherited by F2 and F3 generations were also analysed, observing that many disturbances registered in F1 were carried over to its descendants (F2), this effect being more diluted in F3, as expected. Then, as a consequence of the direct impact of the CTP, DETs recorded in F1 revealed negative regulation of growth due to deficient mineral absorption and changes in the arachidonic acid metabolic process attributed to a disturbed unsaturated fatty acids biosynthesis. Functional analysis of DETs listed in F2 and F3 revealed similar dysregulation in the fatty acid biosynthetic process and/or its metabolism, also unveiling hints of mineral absorption alterations. A thorough examination of the 24 common DETs in all three generations highlighted that some of these DETs have mineral ion binding functions, suggesting that dysregulations in the mineral homeostasis were a common feature among VT animals of all three generations. Besides, we detected DETs encoding a glutathione S-transferase and Malic enzyme 1, suggesting alterations in the redox metabolism. Curiously, these common DETs were not always switched in the same direction, suggesting that there are different underlying causes for the changes in molecular patterns in each generation. In contrast, fatty acid-binding protein 4 appeared downregulated in all three generations. Altogether, this information demonstrated that VT livers presented clear evidence of dysregulations in lipid metabolism. In addition, some DETs recorded in each generation denoted alterations in the carbohydrate metabolism, cellular structure and function, as well as immune system responses.

### Comparative study of the liver metabolome

In each generation, the metabolome profiling of adult liver tissue was compared between VT and NC animals. First of all, we carried out an untargeted metabolomic analysis to gain a general overview of the metabolic changes in all the comparisons under study. After subsequent retrieval of all detected ions, we identified 151, 190 and 159 differentially accumulated metabolites (DAMs) in F1, F2 and F3, respectively (p < 0.05). The variability among semi-polar and non-polar metabolites was investigated by building PCA diagrams, which clustered the samples according to their VT or NC origin in the three generations (Fig. 3A). Overall DAMs variation across generations indicated that VT livers showed an up-accumulation of semi-polar metabolites (average fold change of 0.22, 0.36 and 0.82 for F1, F2 and F3 generation, respectively), but a down-accumulation of non-polar ones except for the F2 generation (average fold change of − 0.36, 0.19 and − 0.67 for F1, F2 and F3 generation, respectively). This fact suggested a high concordance between non-polar DAMs and DETs, as a high proportion of DETs were downregulated in F1 and F3, but upregulated in F2.

To better investigate changes in known liver metabolites, we performed a targeted metabolomic analysis in which we quantified, relatively, 109 metabolites involved in primary (sugars, amino acids, organic acids, lipids, etc.) metabolism. The complete metabolite dataset was reported in Supplementary Table S13. Specifically, 50, 41 and 47 DAMs were detected in F1, F2 and F3 generations. Globally, we identified that most of these DAMs belonged to the non-polar fraction of the metabolome in all three generations (37 [74.0%], 28 [68.3%] and 42 [89.4%] in F1, F2 and F3, respectively). In concordance, as shown in Venn diagrams (Fig. 3B), a high proportion of non-polar DAMs recorded in F1 after CTP were also present in generations F2 and F3. In contrast, semi-polar DAMs appeared most discrete in F1 after CTP and, although some of them were also present in F2, were mostly restored in F3. Functional clustering of DAMs in each generation revealed direct, intergenerational and transgenerational effects of CTP over several metabolic pathways, including glycolysis, gluconeogenesis, citrate cycle, biosynthesis of amino acids, biosynthesis of unsaturated fatty acids and those stemming from the metabolism of arachidonic acid, cholesterol, glycerolipids, glycerophospholipids and sphingolipids. Some of these observations agreed with the transcriptomic study and, in the same manner, DAMs did not always switch in the same direction between generations. Focussing on the common DAMs in all three generations, only 2 (oxalosuccinate and tryptophan) belonged to the semi-polar fraction; meanwhile, 13 belonged to the non-polar fraction. Of the latter, 7 were triglycerides and 6 were related to the unsaturated fatty acids biosynthetic pathway. Among them, we found that arachidonic acid (ARA), and thereby several DAMs for which ARA acts as a precursor (eicosanoids: leukotrienes, prostaglandins and thromboxanes), also displayed dysregulated levels in VT samples.

## Discussion

Several studies have reported that early embryo manipulation can trigger developmental consequences both in human and animal models^4,7,12–15,24^. Nevertheless, no previous reports have examined the transgenerational effects of the embryo cryopreservation-transfer procedure (CTP). Our findings demonstrate, for the first time, the transgenerational inheritance of changes induced by embryo exposure to CTP over the offspring growth performance, adult body weight, phenotype of vital organs and their molecular physiology and metabolism. Deviations in the growth pattern and phenotype have been described by several authors after ART, evidencing that different procedures applied to different genotypes would lead to different outcomes, probably through specific epigenetic modifications^17,24,32–38^. Besides, several studies described ART related organ changes^29,30,33,36,38^. The transgenerational effects occur when alterations in the epigenetic marks, caused by embryo manipulation, persist into subsequent generations despite the extensive reprogramming that takes place both in gametes and in the early embryo^27,28^. This transgenerational epigenetic inheritance has been well documented in plants, nematodes and flies, but its occurrence in mammals, and particularly in humans, remains controversial^39,40^. However, a few disclosed that ART-induced effects could be heritable and persist transgenerationally^29,30,41,42^. In agreement, here we demonstrated that the evidence of transgenerational effects on body and organ weight variations is consistent, but in a tissue-specific manner. In F1, VT animals showed a lower weight in liver and heart, as previously described^26,33^. Concordantly, some authors also reported evidence of liver and heart weights changes after in vitro embryo culture, but only the hepatic disturbances showed a transgenerational inheritance^28,38^. Taken together, these data suggest that the liver could be a highly sensitive organ to ART. Indeed, heart affections could be caused by liver disturbances, given the strong interaction between the physiology of both organs^43^. In this context, placental dysfunction described after embryo cryopreservation^20,21^ could partly explain this liver disturbance, as decreased maternofoetal nutrition during gestation produces both reduced liver mass and perturbed liver function^44^. Intriguingly, weight decrease in the liver and heart was intensified in F2-VT animals. This generation came from gametes originated from primordial germ cells (PGCs) developed in F1 embryos exposed to CTP^45^. As PGCs require meticulous epigenetic dynamics for their proper development^46^, feasible epigenetic modifications induced in PGCs may be more marked, explaining to some extent the higher organ differences in F2-VT animals. Furthermore, we found an interaction between the experimental group and the generation for liver weight, which was lighter in F1- and F2-VT animals, but more prominent in the F3-VT animals. Notably, we observed organomegaly for lungs and spleen in F2-VT and F3-VT animals respectively, but not in F1. Mahsoudi et al*.*^30^ described similar effects after in vitro culture, reporting interactions between generations and treatment for organ weights, and the appearance of some varying phenotypes in F2 that were absent in F1 animals. These authors suggested that different underlying causes for the phenotypic changes emerge in each generation. Indeed, the variability and tissue specificity of the available data indicate that if master regulator genes are present, their cellular framework is elusive^15^. A plausible explanation might be that the epigenetic status in each generation could differ because when an epigenetic change caused by direct experience is transmitted to the offspring, that same experience becomes an indirect environmental trigger for the ontogenetic development of the new individual^47^. Also, in each generation, an extensive epigenetic reprogramming takes place upon fertilisation to remodel the previously acquired epigenetic marks and produce totipotent zygotic states^28,48^. Therefore, how the molecular mechanisms interact to orchestrate the developmental programme may explain the range of results obtained in each generation. Notably, concordantly with the two waves of epigenetic reprogramming that occurred from F1 to F3, some CTP-induced phenotypic differences disappeared or were ameliorated in F3 animals, suggesting a partial restoration of the CTP-induced epigenetic disorders. Therefore, if the advent of ART disrupts this crucial epigenetic rearrangement in F1, it could be that the same mechanism repaired some of the induced deviations two generations later.

Many investigations have also confirmed that ART induces molecular changes in the preimplantation embryo and beyond parturition, where it was associated with some of the ART-induced phenotypes^14,15^. Here, we assessed the transgenerational effects of CTP on the gene expression and metabolic profiles in liver tissue, an organ that supports growth and regulates metabolism from the foetal stage^49,50^. We found that transcriptomic and metabolomic PCA analysis data revealed separate clusters between both experimental groups in each generation. Consequently, 642, 447 and 905 DETs, and 151, 190 and 159 DAMs (untargeted data) were detected in F1, F2 and F3, respectively. Among the DETs recorded in F1, we detected a subset related to the “negative regulation of growth” biological process. Besides, 3 of these DETs were among the most downregulated transcripts (average fold change < − 4) and encode metallothioneins (MT), small proteins involved in zinc (Zn) trafficking and protective mechanisms against oxidative stress and toxic metals^51^. MT are essential for embryonic liver development, and MT deficiency impairs hepatocyte development and provokes liver deterioration at later stages^52^, which could explain the lower hepatic weight at adulthood in VT animals. Notably, MT expression is linked directly to zinc availability^51,53^, so downregulation of MT coding genes could suggests lower Zn abundance in VT livers. KEGG analysis reveals that lower Zn levels might be caused due to an impaired Zn absorption in the intestine (“mineral absorption” pathway), as the body zinc is replenished daily through diet^53^. Concordantly, a recent study described that serum Zn levels in children born via ART were significantly decreased^54^. As Zn is required for healthy growth, its deficiency contributes to growth retardation, and thereby Zn-deficient ART children were smaller than those conceived naturally^54^. Therefore, the lower growth performance exhibited by VT rabbits strengthens this study, supporting that Zn deficiencies could be related to the phenotype in question. Interestingly, dysregulations in MT encoding genes were also found in F2 and F3 VT animals.

In addition, downregulation of DETs encoding for Zn-binding proteins was found in the three generations. Transcriptional mechanisms are present to reduce gene expression of Zn-binding proteins when zinc is limiting, thus conserving Zn for more essential functions^55^. Related to this, some of the common DETs in the three generations have mineral ion binding functions, suggesting that mineral homeostasis dysregulation could be a transgenerational-inherited effect of the CTP. Furthermore, Zn status may affect fatty acid (FA) metabolism, as it acts as a cofactor in FA desaturases and elongases enzymes, which are essential for the biosynthesis of long-chain poly-unsaturated FA (LCPUFA) and its metabolic regulation^56,57^. In agreement, Wang et al*.* found a dysregulated profile of these compounds in the livers of ART mice^58^. In the present study, we detected several DAMs in the three generations that are involved in the LCPUFA biosynthetic pathway, which led to a transgenerationally disturbed LCPUFA profile. Besides, among DETs recorded across F1, F2 and F3 generation, several encoded for desaturases or enzymes involved in the LCPUFA metabolism. These findings suggest that LCPUFA biosynthetic and metabolic processes might be impaired by a synergic effect between transcriptional dysregulation in the involved enzymes collectively with their decreased activity due to Zn deficiencies. Several enriched GO and KEGG terms support LCPUFA disturbances, which were validated by the metabolomic data. Among DAMs commonly noted for the three generations, we observed some LCPUFA required for optimal growth and development^59,60^. In addition, it is worth highlighting that common DETs that participate in the “ARA metabolic process” term enrichment were also present in the “negative regulation of growth” term enrichment. It is well established that arachidonic acid (ARA) is decisive for optimal growth and health^60^. ARA and its derivate metabolites, collectively known as eicosanoids, play essential roles for the coordination of cellular differentiation, organogenesis, foetal growth, postnatal growth and development (for review see Hadley et al*.*^60^). In our study, low ARA levels were noted in F1- and F3-VT livers, whereas in F2-VT ARA content resulted increased. This finding is not contradictory, since it might reflect an impaired conversion of ARA to eicosanoids in F2-VT livers, as some of them were simultaneously down-accumulated. It is worth mentioning that in line with the phenotypic traits, molecular changes also reflected specific patterns in each generation. Of note, we encountered a downregulated fatty acid-binding protein in the three generations, which could be associated with the extended alteration in lipid and lipid-related pathways, since it acts as lipid chaperone, crucial proteins for the lipid metabolism and eicosanoid biosynthesis^61^. Despite further research being required, we hypothesise that deficiencies in the metabolism of Zn and LCPUFA could be related to the transgenerational phenotype exhibited by VT animals. Interestingly, Zn and ARA-derived eicosanoids are crucial components for the immune system responses, and their deficiencies increased susceptibility to infections^54,60^. In our study, functional annotation of DETs throughout generations, validated by the metabolomic data, proposed a general dysregulation in the immune system. In agreement with this hypothesis, it has been observed that ART offspring suffer more infections, their development being slower^62^. Authors concluded that individuals that must allocate more energy to immunity and tissue repair due to infection are likely to have less energy to allocate to growth and development. On the other hand, another interesting finding of our study was that both LCPUFAs and ARA are fundamental components in the mitochondrial and cellular membranes of the liver and almost all other organs, aiding in the synthesis of phosphatidylcholines^60,63,64^. In agreement, we detected a wide range of down-accumulated phosphatidylcholines in the VT livers along the three generations. Cell membranes are the basis of the organisation of the cell and changing its composition alters its function^60,63,64^, which could contribute to the physiological and metabolic differences between VT and NC animals. This event could explain the range of GO term and KEGG routes obtained for DETs and related to cellular structures and functions in each generation, some of which are conserved from F1 to F2 and F3.

Specifically, LCPUFAs and eicosanoids are endogenous ligands for PPARs, a superfamily of nuclear transcription factors responsible for upregulating genes of key mitochondrial enzymes^63^. KEGG analysis revealed disturbances in the “PPAR signalling pathway” for F1- and F2-VT animals, which appeared jointly with some DAMs implicated in oxidative phosphorylation (OXPHO). In agreement with our previous proteomic study, we suggest that animals born after a CTP exhibit dysregulation concerning the OXPHO process^26^. These results could explain that VT progeny are susceptible to mitochondrial dysfunctions, which seemed to be restored in F3. In agreement, DAMs related to OXPHO were founded in F1 and F2, but not in F3. Concordantly, Feuer et al*.*^36^ also reported alterations in the postnatal growth trajectory linked to broad changes in metabolic homeostasis, characterised by mitochondrial dysfunction and systemic oxidative stress. As oxidative changes are ubiquitously present in postnatal ART tissues^15^, it might explain the presence of common DETs encoding glutathione S-transferase and malic enzyme 1, which participate in a molecular system to maintain redox balance^65,66^. Besides, malic enzyme 1 links the catabolic pathways of glycolysis and citric acid cycle to the anabolic pathways of fatty acid and cholesterol biosynthesis through NADPH, thus having critical roles in mitochondrial energy metabolism and maintenance of homeostasis^66^. Interestingly, several DAMs participating in these metabolic pathways were found in all generations, suggesting that CTP triggers a metabolic reprogramming in the liver affecting a wide range of interconnected pathways, including those related with metabolism of carbohydrates and amino acids. As reviewed by Feuer’s group^14,15,35^, dysregulations in the metabolism of glucose, amino acids and long-chain FA have been encountered in IVF livers, which were associated with an altered mitochondrial function. Changes in some metabolites have been proposed as potential compensatory effects against those disturbed by ART, but altogether these dysregulations can disrupt optimal energy expenditure and might further contribute to the postnatal phenotypes associated with ART^15^. Altogether, our findings provide strong evidence of these metabolic peculiarities after CTP, which have been reported in IVF mice, and are beginning to be validated in IVF humans^10,67^.

Despite all the changes attributed to CTP, the VT progeny seemed healthy, as no striking difference was detected either during the management or during the dissection study in either generation, in line with our previously reported study^24^. In addition, the fertilising capacity is a measure of health status of the resultant progeny, also used after ART^30,68^. Related to this, ART has been linked with reduced sperm quality in studies performed in mice and humans^29,69^. Reassuringly, here we observed that although some deviations in seminal parameters were recorded, they were within the normal physiological range of variability^70^ and seemed irrelevant, because a similar fertility rate was observed between VT and NC progenies. These results indicated that CTP is not reproductive health-related. In addition, we found that the number of liveborn was transgenerationally increased in VT animals, reinforcing our hypothesis postulated years ago that suggested epigenetic mechanisms as the underlying cause^41^. These findings highlight that further research is needed to investigate whether these effects are attributable to a legacy inherited through mother or/and father. Both positive and adverse health effects have been observed after embryo cryopreservation^18^ based on a still misunderstood embryonic plasticity, which refers to the capacity of a genotype to produce different phenotypes in response to environmental changes through epigenetic mechanisms^4, 28,71^. Recent evidence supports the relevance of the ART stressors on early embryos, triggering a high range of self-reprogramming that can be condition- and strain-specific, sexually dimorphic and may not emerge until later into adulthood^15^. Besides, without being exclusive, we hypothesised that cryopreservation could act as a selection pressure (“cryo-selection”), since not all embryos survive this process, which could favour the inheritance of alleles responsible for this deviant phenotype in VT animals^24^. However, although the rabbit is considered an excellent reproductive model for human health^25^, caution is required when extrapolating results from rabbit studies to humans.

In conclusion, our results support and extend the list of studies reporting ART effects, but mainly contributed to the knowledge regarding long-term transgenerational effects. This study should serve as a discussion table to conceive new studies that evaluate related effects on females and evaluating another current ART, thus determining the most efficient and safest techniques to obtain offspring.

## Materials and methods

All chemicals, unless otherwise stated, were reagent-grade and purchased from Sigma-Aldrich Química SA (Alcobendas, Madrid, Spain).

### Experimental design

California breed rabbits were used for the experiment^72^. Two experimental groups were established: one from vitrified embryos transferred to foster mothers (VT progeny) and another generated by natural conception (NC progeny). At birth, animals constituted the F1 generation, in which both VT and NC animals were compared to address the direct effects of the embryo cryopreservation-transfer procedure (CTP). After that, the intergenerational effects were assessed in the F2 generation. Finally, the transgenerational effects were evaluated in the F3 generation, comparing the VT and NC progenies. This is because the direct effect of the CTP was present in the embryos which formed the F1 generation and over the germline developing within the embryo that ultimately formed the F2 animals. Therefore, the F3 generation is the first not directly exposed to the CTP^45^. Figure 1 illustrates the experimental design.

The NC- and VT-F1 animals belonged from Garcia-Dominguez et al.^26^. These animals constituted the founding generation from which F2 and F3 were here obtained. Briefly, a total of 27 females were artificially inseminated and ovulation was induced by an injection of 1 µg of buserelin acetate (Hoechst Marion Roussel, Madrid, Spain). In the VT group, from 13 females, 3-days embryos were recovered, vitrified-thawed and transferred oviductally to 13 foster mothers (10–14 embryos per female). A total of 158 vitrified-thawed embryos were transferred (95.3% survival rate after thawing). Meanwhile, for the NC population, contemporaneous animals were generated at birth from the remaining 14 females initially inseminated. The subsequent generations were generated respecting the experimental groups and following the common management of rabbit reproduction, without embryo vitrification or embryo transfer procedures. To establish the F2 generation, one mature female and one male were randomly selected from each parity produced in the F1 generation and, to reduce inbreeding, mating between animals with common grandparents was avoided. The F3 animals were generated equally from the F2 as described above. In rabbits, it has been demonstrated that an effective preservation of characteristics, such as growth rate and litter size, could be obtained with the offspring of 9 males from different reproductive groups (families), guaranteeing an inbreeding coefficient value of less than 1% per generation^73^. Considering F2 as a transient population towards F3 generation, only those necessary animals were kept until adulthood. On the contrary, as the effects of CTP could be more diluted or evasive in the F3 generation, a larger number of F3 animals were maintained until adulthood. Animals of both progenies in each generation were housed in the same conditions throughout the experiment. Weaning took place at week 4. Until 9 weeks of age animals were caged collectively (8 rabbits per cage), and subsequently males were housed individually (flat deck indoor cages; 75 × 50 × 40 cm). In order to reduce confounding factors, the phenotypic and molecular analyses were restricted to males, as they are thought to be less variable due to their constant hormone levels^74^. However, F2- and F3-VT animals were obtained by crossing VT males and VT females from the previous VT generation, as inheritable CTP effects could be transmitted through both genders.

### Embryo vitrification and transfer procedure

Embryos were vitrified and thawed according to the highly efficient protocol developed previously to cryopreserve rabbit embryos by vitrification^75^. This protocol allows the survival of > 80% of the thawed embryos, having generated thousands of descendants in our own laboratory since its implementation^25^. Briefly, vitrification was achieved in two steps. In the first step, embryos were placed for 2 min in a solution consisting of 10% (v/v) dimethyl sulfoxide (DMSO) and 10% (v/v) ethylene glycol (EG). In the second step, embryos were suspended for 1 min in a solution of 20% DMSO and 20% EG. Then, embryos suspended in vitrification medium were loaded into 0.125 mL plastic straws, which were sealed and plunged directly into liquid nitrogen to achieve vitrification. Embryos were warmed in 2 mL of 0.33 M sucrose solution at 25 °C. After 5 min, the embryos were washed and scored, and only undamaged embryos (presenting homogenous cellular mass, mucin coat and spherical zona pellucida) were catalogued as transferable. Then, embryos were transferred into the oviduct of synchronous foster mothers by laparoscopy, following the protocol described by Besenfelder and Brem^76^. Briefly, foster mothers were anaesthetised and placed in Trendelenburg's position. Then, embryos were loaded in a 17G epidural catheter, which was inserted through a 17G epidural needle into the inguinal region. Finally, while the process was monitored by single-port laparoscopy, the catheter was introduced in the oviduct through the infundibulum to release the embryos. Both embryo vitrification and transfer process used in this experiment were described in detail in our recent report^25^.

### Growth, body weight and organs weight study

**Figure 4:**
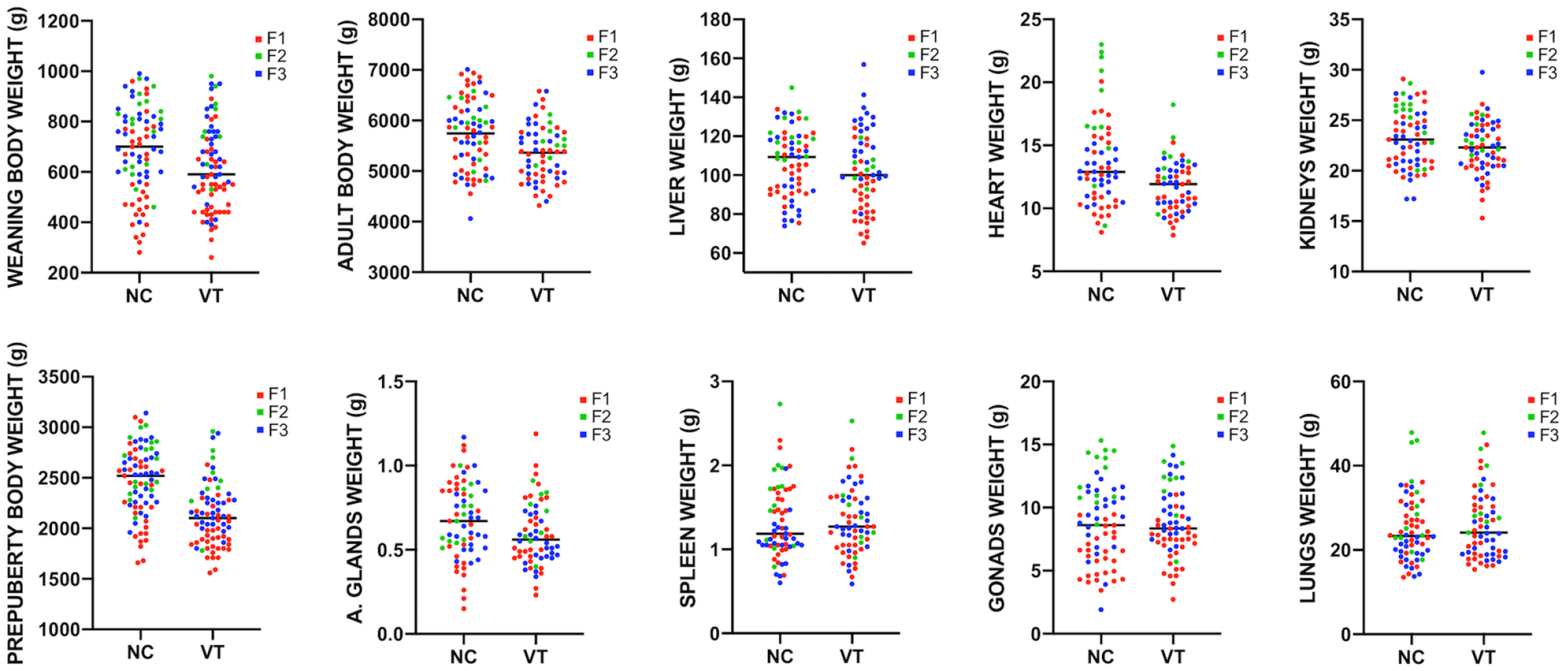
Scatterplots showing the phenotypic raw data distributions of naturally-conceived (NC) and vitrified-transferred (VT) progenies during three generations (F1, F2, F3).

Body weights from both progenies were compared in each generation at three relevant points of rabbit development: 4, 9 and 56 weeks of age, coinciding with the weaning, prepubertal and adulthood stages, respectively. Weights of both progenies (NC and VT) were compared in each generation. Furthermore, weights at 4th and 9th weeks comprise a period when the rabbit growth is exponential. Using these values, the average weight gain (AWG) was calculated to estimate the growth velocity. AWG was computed as [9th week weight – 4th week weight]/35 days. Finally, after the last weight was taken, adult males from both groups (65 F1, 22 F2 and 48 F3) were euthanised by barbiturate overdose (125 mg/kg) and the weights of liver, lungs, heart, kidneys, adrenal glands, spleen and gonads were recorded and compared. Scatterplots showing the phenotypic raw data distributions of both experimental groups before statistical analysis were plotted (Fig. 4) using GraphPad PRISM (8.3.0).

### Semen collection and sperm evaluation

The procedure was conducted as previously described^70^. Briefly, in each generation, the training period for male rabbits (23 F1, 18 F2, and 28 F3, distributed equitably between VT and NC groups) began with an artificial vagina at 18 weeks of age, collecting one ejaculate per week. At the 6th month of age, males were subjected to experimental evaluation. For 12 weeks, two ejaculates per male were collected weekly, with an interval of 30 min between collections. Ejaculates from the same male each day were pooled, and three 20 μL aliquots were taken. The first and second were diluted 1:20 with Tris-citrate-glucose extender (250 mM tris-hydroxymethylaminomethane, 83 mM citric acid, 50 mM glucose, pH 6.8–7.0, 300 mOsm kg^−1^). The first was assessed for individual sperm motility and motion parameters using the Integrated Semen Analysis System version 1.0.17 (ISAS; Projectes i Serveis R + D). The system was set to record images at 25 frames/s. Then, 10 µL of the sample was placed in a 10 -µm deep Makler counting chamber. Sperm motility was assessed at 37 °C at × 200 magnification using a negative phase-contrast microscope. For each sample, four microscopic fields were analysed, and a minimum of 200 sperm evaluated. The following sperm activity variables were assessed: sperm motility (%), progressive motility (%), curvilinear velocity (VCL, μm s^−1^), straight-line velocity (VSL, μm s^−1^), average path velocity (VAP, μm s^−1^), linearity coefficient (LIN; calculated as (VSL/VCL) × 100, %), straightness coefficient (STR), wobble coefficient (WOB; VSL/VAP × 100), amplitude of lateral head displacement (ALH, μm) and beat cross frequency (BCF, Hz). The second sample was assessed for the percentage of live spermatozoa (viability, VIA) using the LIVE/DEAD sperm viability kit (Molecular Probes), which basically consists of two DNA-binding fluorescent dyes: a membrane-permeant dye, SYBR-14, and a conventional dead-cell dye, propidium iodide. The third sample was also diluted 1:20 with 0.5% of glutaraldehyde solution in phosphate-buffered saline and observed by phase-contrast at × 400 magnification to calculate the concentration, in a Thoma-Zeiss counting cell chamber, and both the percentages of intact apical ridge and abnormal sperm were evaluated (based on morphological abnormalities of head, neck, mid-piece and tail). A total of 249, 134 and 255 ejaculates were evaluated in the F1, F2 and F3 generation, respectively.

### Fertility rate and number of liveborn

The procedure was conducted as previously described^70^. Briefly, a total of 1,260 inseminations (260 F1, 775 F2 and 225 F3, distributed equitably between VT and NC groups) using individual ejaculates adjusted to 40 × 10^6^ spermatozoa/mL were carried out. Each female was inseminated with 0.5 mL, which was performed within two hours of semen collection. At insemination time, females were injected intramuscularly with 1 μg of buserelin acetate (Hoechst Marion Roussel, Madrid, Spain) to induce ovulation. Only receptive does (red colour of vulvar lips) were inseminated, using a standard curved plastic pipette (Imporvet, Barcelona, Spain). The number of does that gave birth (fertility rate) by the number of inseminations was recorded. Likewise, the number of liveborn per parity was annotated.

### Statistical analyses

Descriptive statistics of the quantitative traits (litter size, body weight, growth, organ weights, sperm parameters and live born) were estimated with data from all generations (Supplementary Table S1). After that, differences due to vitrified embryo transfer procedure on these traits in each generation were estimated using Bayesian inference. This methodology is based on probabilities, providing great flexibility to construct all kinds of confidence intervals with a chosen probability. In all cases, the progeny origin was included as a treatment with two levels (NC and VT) and the organ weights were corrected by the adult body weight of each animal. Bounded flat priors were used for all unknowns and the marginal posterior distributions were estimated by Gibbs sampling. After some exploratory analyses, results were based on Markov chain Monte Carlo chains consisting of 60,000 iterations, with a burn-in period of 10,000, and saving only 1 of every 10 samples for inferences. Summary statistics from the marginal posterior distributions were calculated directly from the samples saved. Convergence was tested using the Geweke Z criterion and Monte Carlo sampling errors were computed using time-series procedures. In all cases, Monte Carlo SE were small and lack of convergence was not detected by the Geweke test. The parameters obtained from the marginal posterior distributions of the phenotypic differences between experimental groups were the mean of the difference (D_NC-VT_; computed as NC-VT), the probability of the difference being greater than 0 when D_NC-VT_ > 0 or lower than 0 when D_NC-VT_ < 0 (P_0_), and the highest posterior density region at 95% of probability (HPD_95%_). D_NC-VT_ estimated the mean of the differences between NC and VT traits, P_0_ estimated the probability of D_NC-VT_ ≠ 0, and HPD_95%_ estimated the accuracy. Statistical differences were considered if |D_NC-VT_| surpassed the relevant value (R; proposed as one-third of the SD of the trait) and P_0_ > 0.8 (80%). Statistics analysis were computed with the Rabbit program developed by the Institute for Animal Science and Technology (Valencia, Spain). A more detailed description of these features can be found in a previous review^77^.

Differences due to vitrified embryo transfer procedure on fertility rate were assessed using a probit link model with binomial error distribution, according to a mixed model including the experimental group (NC vs VT) as fixed effect. These statistical analyses were performed by the SPSS statistical software package, version 21.0 (SPSS Inc., Chicago, Illinois, USA).

### Transcriptomic analysis of the liver

After the euthanasia was performed, individual hepatic samples were collected by retrieving biopsies randomly from different liver sites and mixing them (one individual, one sample). Finally, 23 liver samples were collected from the F1 (5 VT and 4 NC), F2 (3 VT and 3 NC) and F3 (4 VT and 4 NC) generations. Immediately, samples were washed with phosphate-buffered saline (PBS) to remove blood remnants and stored in RNAlater (Ambion Inc., Huntingdon, UK) at − 20 °C until the transcriptomic analysis. Total RNA of liver was extracted using Ambion (mirVana) and Qiagen (AllPrep) columns. The RNA quantity and quality were determined on a bioanalyser (Agilent Technologies), keeping samples with RIN values > 8 and with > 3 μg of total RNA for sequencing. Then, samples were shipped to the Macrogen company (Seoul, South Korea). Afterwards, the mRNA purification was carried out using Sera-mag Magnetic Oligo (dT) Beads, followed by buffer fragmentation. Reverse transcription was followed by PCR amplification to prepare the samples to be sequenced, keeping the strand information, in an Illumina Hiseq-4000[D1] sequencer (Illumina, San Diego, USA). Resulting raw sequences are available at the NCBI Sequence Read Archive (BioProject ID: PRJNA483096). Raw read qualities were assessed using FastQC software^78^. Only samples with good quality scores were maintained for the final analysis. Reads were mapped against the reference genome for Oryctolagus cuniculus, version 2.0 from Ensembl using HISAT2^79^. Expression was counted using StringTie^80^. This counting was guided using the genome annotation, and a unified set of transcripts was created for the samples analysed. Then, a Fragments Per Kilobase of transcript per Million (FPKM) table with gene expression for each sample was generated and used to assess the expression profile of each sample by PCA (Supplementary Fig. S1). Then, a table with raw counts was generated. This table was used for the differential expression analyses using edgeR^81^ integrated into the MeV package^82^. Only DETs with a threshold of a false discovery rate (FDR) of ≤ 0.05 were considered for further analyses. For comparison between groups, further filtering of DETs was performed. In those samples that registered a coefficient of variation higher than 50% and a difference between mean and median higher than 1, the gene was maintained if half of the samples of the most expressed condition group had an expression two times higher than the mean of the other group. ClustVis^83^ software was used to perform the PCA of DETs. InteractiVenn^84^ software was used for Venn diagram construction. Functional annotation of DETs, enrichment analysis of their associated GO terms and KEGG pathways analysis were computed using bioinformatics software: DAVID Functional Annotation Tool 6.8^85^, considering a P-value < 0.05 (modified Fisher's exact test, EASE score).

### Semi-polar and non-polar metabolomic liver analysis

Targeted and untargeted LC–ESI–MS analyses of the hepatic semi-polar metabolome were performed as previously described^86–88^, while targeted and untargeted LC-APCI-MS analyses of the hepatic non-polar metabolome were carried out as reported before^89–91^. Untargeted metabolomics was performed using the SIEVE software (Thermofisher scientific). Briefly, after chromatogram alignment and retrieval of all the detected frames (e.g. ions), differentially accumulated metabolites (DAMs) were detected by a statistical analysis (one-way ANOVA plus Tukey’s pairwise comparison) using the SPSS software (SPSS Inc., Chicago, Illinois, USA). PCA of untargeted metabolomes was performed using the ClustVis^83^ software. Targeted metabolite identification was performed by comparing chromatographic and spectral properties with authentic standards (if available) and reference spectra, in house database, literature data, and on the basis of the m/z accurate masses, as reported in the Pubchem database (https://pubchem.ncbi.nlm.nih.gov/) for monoisotopic mass identification, or on the Metabolomics Fiehn Lab Mass Spectrometry Adduct Calculator (https://fiehnlab.ucdavis.edu/staff/kind/Metabolomics/MS-Adduct-Calculator/) in the case of adduction detection. Finally, DAMs were detected as described before. Metabolites were quantified relatively by normalisation on the internal standard (formononetin and DL-α-tocopherol acetate) amounts. For each experimental group, 3 independent biological replicates, consisting of 4 animals each, were analysed in each generation. For each biological replicate, at least one technical replicate was carried out.

### Ethical statements

The study was approved by Universitat Politècnica de València Ethical Committee (Code: 2015/VSC/PEA/00061). The study followed the Directive 2010/63/EU EEC guidelines. Experimental protocols were conducted under the supervision of the animal welfare committee in charge of this animal facility. An authorisation certificate issued by the Valencian governmental administration to experiment on animals is held by X. GD (code: 2815), F. MJ (code: 2273) and JS. V (code: 0690).

## Supplementary information

Supplementary information.
