# Supplementary material for: Long-term and transgenerational phenotypic, transcriptional and metabolic effects in rabbit males born following vitrified embryo transfer
Source: Sci Rep. 2020 Jul 9;10:11313. doi: 10.1038/s41598-020-68195-9 (PMC7347584; doi:10.1038/s41598-020-68195-9)
Supplement: Supplementary file 1 — Supplementary information. [file 41598_2020_68195_MOESM1_ESM.pdf]

SUPLEMENTARY MATERIAL

Long-term and transgenerational phenotypic, transcriptional and metabolic effects in rabbit males born following vitrified embryo transfer

Ximo Garcia-Dominguez[1], Francisco Marco-Jiménez[1,+], David S Peñaranda[1], Gianfranco Diretto[2], Víctor Garcia-Carpintero[3], Joaquín Cañizares[3], Jose S Vicente[1,+,*]

[1]Laboratory of Biotechnology of Reproduction, Institute for Animal Science and Technology (ICTA), Universitat Politècnica de València, 46022 Valencia, Spain.

[2]National Agency for New Technologies, Energy and Sustainable Economic Development (ENEA), Casaccia Research Centre, 00123 Rome, Italy.

[3]Institute for the Conservation and Breeding of Agricultural Biodiversity (COMAV-UPV), Universitat Politècnica de València, 46022 Valencia, Spain.

Supplementary Table S1. Descriptive analysis of the evaluated quantitative traits.

Traits		n	Mean	SEM	SD
**Litter size* (pups)**		67	5.8	0.30	2.4
**Body weight (g)**	Weaning (4 weeks)	160	650.3	13.88	179.6
	Prepuberty (9 weeks)	154	2302.5	29.72	368.9
	Adult (56 weeks)	135	5566.7	56.58	657.4
**Growth (g/day)**	Average weight gain	154	47.0	0.56	6.9
**Organ weight (g)**	Liver	126	104.4	1.67	18.8
	Heart	120	12.7	0.28	3.0
	Lungs	127	25.5	0.70	7.9
	Spleen	127	1.3	0.04	0.4
	Kidneys	128	22.8	0.25	2.8
	Gonads	128	8.7	0.26	3.0
	Adrenal Glands	127	0.6	0.02	0.2
**Sperm traits**	VOL (mL)	633	0.7	0.02	0.4
	CON (10^6)	630	242.1	6.37	159.9
	TSE (10^6 spz)	623	145.8	4.34	108.4
	MOT (%)	619	68.7	1.03	25.6
	PRO (%)	633	39.8	0.77	19.4
	VIA (%)	615	75.5	0.45	11.2
	NAR (%)	605	90.8	0.26	6.4
	ABN (%)	626	20.7	0.36	9.1
	VCL ($\mu m\ s^{-1}$)	636	98.1	0.84	21.1
	VSL ($\mu m\ s^{-1}$)	638	42.2	0.57	14.3
	VAP ($\mu m\ s^{-1}$)	636	60.4	0.67	16.8
	LIN (%)	631	43.8	0.63	15.9
	STR (%)	627	69.1	0.38	9.6
	WOB (%)	634	61.6	0.50	12.6
	ALH (µm)	625	2.9	0.02	0.6
	BCF (Hz)	620	11.2	0.08	2.0
	Live births# (pups)	383	5.5	0.18	3.2

n is the number of data in each trait; * Litter size is the number of pups per parity that constituted each generation; **VOL**: Ejaculate volume; **CON**: Spermatic concentration; **TSE**: Total sperm per ejaculate; **spz:** spermatozoa; **MOT**: Percentage of sperm motility; **PRO**: Percentage of progressive motility; **VIA**: Percentage of viable sperm; **NAR**: percentage of normal apical ridge; **ABN**: Percentage of abnormal forms; **VCL**: Curvilinear velocity; **VSL**: straight-line velocity; **VAP**: average path velocity; **LIN:** linearity coefficient (VSL/VCL × 100); **STR:** straightness coefficient; **WOB:** wobble coefficient (VSL/VAP × 100); **ALH**: amplitude of lateral head displacement; **BCF**: beat cross-frequency; # Live births is the number of live pups derived from the male fertility study in each generation.

Supplementary Table S2. Bayesian analyses of the phenotypic differences between naturally-conceived animals (NC group) and those derived from vitrified-transferred embryos (VT group) during three generations (F1, F2 and F3), computed as NC-VT.

PHENOTYPIC TRAITS[+]	D_{NC-VT}			R	P_0			$HPD_{95\%}$		
	**F1**	**F2**	**F3**		F1	F2	F3	F1	F2	F3
**Litter size** (pups)	**0.2**	**0.5**	**-0.3**	1	0.58	0.67	0.58	-1.7, 2.1	-1.9, 2.9	-3.2, 2.5
**Weaning body weight** (g)	**62.2***	**-26.7**	**59.4**	60	0.94	0.66	0.94	-16.6, 135.6	-152.9, 109.1	-17.9, 134.9
**Prepuberty body weight** (g)	**370.1***	**139.3***	**287.9***	123	1.00	0.85	1.00	210.1, 524.2	-119.5, 405.2	138.1, 422.5
**Average weight gain** (g/day)	**8.6***	**4.7***	**6.6***	2	1.00	0.97	1.00	5.9, 11.3	0.18, 10.3	3.7, 9.4
**Adult body weight** (g)	**437.4***	**249.5***	**247.9***	219	0.99	0.87	0.91	65.5, 801.4	-229.7, 704.3	-129.5, 610.0
**Liver** (g)	**9.3***	**12.7***	**-16.1***	6	0.99	1.00	1.00	2.2, 16.5	4.3, 20.6	-26.7, -6.0
**Heart** (g)	**1.5***	**3.1***	**0.3**	1	0.98	0.93	0.73	0.1, 2.6	-1.3, 7.6	-0.6, 1.2
**Lungs** (g)	**-1.1**	**-3.7***	**-2.3**	3	0.73	0.81	0.90	-4.6, 2.3	-12.9, 4.3	-5.5, 1.3
**Spleen** (g)	**0.1**	**0.0**	**-0.2***	0.1	0.65	0.50	0.99	-0.2, 0.3	-0.4, 0.5	-0.4, -0.1
**Kidneys** (g)	**0.4**	**0.8**	**-1.0**	1	0.71	0.95	0.96	-1.0, 1.7	-0.2, 1.8	-2.2, 0.1
**Gonads** (g)	**-0.7**	**0.7**	**-1.0**	1	0.93	0.71	0.93	-1.8, 0.2	-1.8, 3.1	-2.4, 0.4
**A. Glands** (g)	**-0.0**	**0.0**	**0.1**	0.1	0.58	0.52	0.98	-0.1, 0.1	-0.2, 0.18	0.0, 0.2

[+]Litter size (of births that originate each generation); Weaning (4 weeks of age); Prepuberty (9 weeks of age); Adulthood (56 weeks of age).

D_{NC-VT} = Mean of the difference NC-VT (median of the marginal posterior distribution of the difference between the NC and the VT groups).

R= Relevant value (proposed as one-third of the SD of the trait), rounded to the first significant number.

P_0= Probability of the difference (D_{NC-VT}) being greater than 0 when $D_{NC-VT} > 0$ or lower than 0 when $D_{NC-VT} < 0$.

$HPD_{95\%}$= The highest posterior density region at 95% of probability.

*Statistical differences were assumed if $|D_{NC-VT}|$ surpass R value and its P_0>0.80.

Supplementary Table S3. Bayesian analyses of the sperm/fertility traits differences between naturally-conceived males (NC group) and those derived from vitrified-transferred embryos (VT group) during three generations (F1, F2 and F3), computed as NC-VT.

	D_{NC-VT}			R	P_0			$HPD_{95\%}$		
	**F1**	**F2**	**F3**		F1	F2	F3	F1	F2	F3
**SEMEN PARAMETERS**										
VOL (mL)	**-0.2***	**-0.2***	**0.1**	0.1	1.00	1.00	0.99	-0.3, -0.1	-0.3, -0.1	0.0, 0.1
CON (10^6)	**37.0**	**8.9**	**-1.8**	53	0.97	0.76	0.53	-0.9, 72.6	-15.6, 33.7	-51.9, 44.6
TSE (10^6 spz)	**-22.1**	**-44.8***	**19.3**	36	0.89	1.00	0.96	-60.0, 13.8	-64.3, -24.9	-3.0, 40.2
MOT (%)	**-5.0**	**1.5**	**-1.7**	9	0.99	0.94	0.68	-9.3, -0.3	-0.3, 3.5	-9.1, 4.9
PRO (%)	**-2.5**	**-3.9**	**-2.0**	6	0.89	0.98	0.80	-6.5, 1.7	-7.7, -0.2	-6.7, 2.7
VIA (%)	**0.4**	**-0.4**	**-0.1**	4	0.62	0.65	0.53	-2.3, 3.0	-2.4, 1.7	-3.4, 3.1
ABN (%)	**-1.1**	**3.4***	**-0.3**	3	0.83	1.00	0.58	-3.6, 1.1	1.7, 5.3	-3.1, 2.2
NAR (%)	**0.0**	**0.1**	**0.7**	2	0.52	0.57	0.80	-1.9, 2.0	-1.6, 1.6	-0.8, 2.3
**MOTION PARAMETERS**										
VCL (µm s^{-1})	**-13.3***	**3.5**	**8.1***	7	1.00	0.95	1.00	-19.0, -7.8	-0.6, 7.8	2.2, 14.0
VSL (µm s^{-1})	**-5.5***	**0.7**	**0.6**	5	1.00	0.65	0.63	-8.9, -2.1	-2.7, 4.1	-2.8, 3.9
VAP (µm s^{-1})	**-10.0***	**1.2**	**2.4**	6	1.00	0.72	0.88	-14.4, -5.4	-2.7, 4.9	-1.5, 6.3
LIN (%)	**-1.7**	**1.7**	**-1.9**	5	0.74	0.90	0.92	-6.7, 3.2	-0.8, 4.6	-4.7, 0.9
STR (%)	**1.8**	**1.7**	**-1.5**	3	0.92	1.00	0.88	-0.9, 4.3	0.5, 3.0	-4.2, 0.9
WOB (%)	**-2.1**	**-0.9**	**-1.8**	4	0.90	0.73	0.91	-5.2, 1.1	-3.8, 1.9	-4.4, 0.7
ALH (µm)	**-0.1**	**0.0**	**0.2**	0.2	0.98	0.73	0.99	-0.3, -0.0	-0.1, 0.2	0.0, 0.4
BCF (Hz)	**-0.4**	**0.0**	**0.4**	1	0.96	0.56	0.90	-0.8, 0.1	-0.5, 0.6	-0.2, 0.9
**Litter size**										
Live births (pups)	**-1.1***	**-1.1***	**-2.8***	1	0.94	0.99	1.00	-2.6, 0.3	-2.1, -0.1	-5.0, -0.8

D_{NC-VT} = Mean of the difference NC-VT (median of the marginal posterior distribution of the difference between the NC and the VT groups).
R= Relevant value (proposed as one-third of the SD of the trait), rounded to the first significant number.
P_0= Probability of the difference (D_{NC-VT}) being greater than 0 when $D_{NC-VT} > 0$ or lower than 0 when $D_{NC-VT} < 0$.
$HPD_{95\%}$ = The highest posterior density region at 95% of probability.
*Statistical differences were assumed if $|D_{NC-VT}|$ surpass R value and its P_0>0.80.

Supplementary Table S4. Differentially expressed transcripts in liver tissue between animals born from vitrified- transferred embryos and those conceived naturally.

Gene accession	Gene name	Fold change
ENSOCUG00000025868	gamma-aminobutyric acid type A receptor delta subunit(GABRD)	-7,00
ENSOCUG00000015329	matrix metallopeptidase 7(MMP7)	-5,52
ENSOCUG00000021126	Metallothionein-2A(LOC100343299)	-5,27
ENSOCUG00000003467	prostate stem cell antigen(PSCA)	-5,13
ENSOCUG00000004004	family with sequence similarity 135 member B(FAM135B)	-4,78
ENSOCUG00000011488	Fanconi anemia complementation group I(FANCI)	-4,34
ENSOCUG00000027388	UDP-GlcNAc:betaGal beta-1,3-N-acetylglucosaminyltransferase 8(B3GNT8)	-4,24
ENSOCUG00000010513	centrosomal protein 55(CEP55)	-4,23
ENSOCUG00000005159	anillin actin binding protein(ANLN)	-4,15
ENSOCUG00000008303	matrix metallopeptidase 12(MMP12)	-4,15
ENSOCUG00000002934	zinc finger protein 280C(ZNF280C)	-4,10
ENSOCUG00000001891	glycerol-3-phosphate acyltransferase 2, mitochondrial(GPAT2)	-4,02
ENSOCUG00000017659	alanyl aminopeptidase, membrane(ANPEP)	-4,00
ENSOCUG00000006444	CD109 molecule(CD109)	-3,99
ENSOCUG00000000374	ADAM metallopeptidase with thrombospondin type 1 motif 15(ADAMTS15)	-3,96
ENSOCUG00000005115	fibroblast activation protein alpha(FAP)	-3,91
ENSOCUG00000002542	solute carrier family 22 member 2(SLC22A2)	-3,84
ENSOCUG00000013111	procollagen C-endopeptidase enhancer 2(PCOLCE2)	-3,82
ENSOCUG00000001726	Wnt family member 11(WNT11)	-3,81
ENSOCUG00000012881	collagen type I alpha 1 chain(COL1A1)	-3,77
ENSOCUG00000024161	potassium voltage-gated channel subfamily J member 15(KCNJ15)	-3,70
ENSOCUG00000013577	family with sequence similarity 171 member A2(FAM171A2)	-3,70
ENSOCUG00000021209	metallothionein-2D(LOC100343557)	-3,69
ENSOCUG00000002945	mesenchyme homeobox 1(MEOX1)	-3,67
ENSOCUG00000015320	collagen type XII alpha 1 chain(COL12A1)	-3,63
ENSOCUG00000021508	chromosome 16 open reading frame, human C1orf106(C16H1orf106)	-3,62
ENSOCUG00000017320	ring finger protein 224(RNF224)	-3,59
ENSOCUG00000000856	E2F transcription factor 1(E2F1)	-3,59
ENSOCUG00000001171	NUF2, NDC80 kinetochore complex component(NUF2)	-3,57
ENSOCUG00000000360	cadherin 6(CDH6)	-3,51
ENSOCUG00000001719	diaphanous related formin 3(DIAPH3)	-3,51
ENSOCUG00000002407	microfibrillar associated protein 5(MFAP5)	-3,48
ENSOCUG00000022280	transmembrane protein 45B(TMEM45B)	-3,47
ENSOCUG00000016157	exonuclease 1(LOC100338764)	-3,46
ENSOCUG00000001376	sodium channel protein type 11 subunit alpha(LOC100349709)	-3,46
ENSOCUG00000029235	metallothionein-1A(LOC100343802)	-3,44
ENSOCUG00000025499	uncharacterized protein CXorf21-like(LOC100345885)	-3,43
ENSOCUG00000010144	tetratricopeptide repeat domain 22(TTC22)	-3,39
ENSOCUG00000001652	cyclin B2(CCNB2)	-3,38
ENSOCUG00000001962	4-hydroxyphenylpyruvate dioxygenase like(HPDL)	-3,37
ENSOCUG00000013140	calmodulin binding transcription activator 1(CAMTA1)	-3,33
ENSOCUG00000004616	myotubularin related protein 11(MTMR11)	-3,32

ENSOCUG00000017111	kinesin family member 4A(KIF4A)	-3,31
ENSOCUG00000013056	zinc finger protein 804B(ZNF804B)	-3,30
ENSOCUG00000002924	galectin 3(LGALS3)	-3,29
ENSOCUG00000000120	ADCYAP receptor type I(ADCYAP1R1)	-3,28
ENSOCUG00000003649	transmembrane and immunoglobulin domain containing 1(TMIGD1)	-3,27
ENSOCUG00000000699	centromere protein E(CENPE)	-3,23
ENSOCUG00000015492	glucagon like peptide 2 receptor(GLP2R)	-3,21
ENSOCUG00000005788	solute carrier family 22 member 7(SLC22A7)	-3,20
ENSOCUG00000022624	solute carrier family 26 member 9(SLC26A9)	-3,18
ENSOCUG00000010814	malic enzyme 1(ME1)	-3,16
ENSOCUG00000006258	WD repeat domain 72(WDR72)	-3,15
ENSOCUG00000017347	leptin receptor(LEPR)	-3,15
ENSOCUG00000029254	family with sequence similarity 159 member A(FAM159A)	-3,14
ENSOCUG00000011025	contactin 4(CNTN4)	-3,13
ENSOCUG00000021051	ripply transcriptional repressor 3(RIPPLY3)	-3,12
ENSOCUG00000023285	trophinin associated protein(TROAP)	-3,12
ENSOCUG00000013412	C-C motif chemokine 7(LOC103351517)	-3,09
ENSOCUG00000007151	cilia and flagella associated protein 70(CFAP70)	-3,07
ENSOCUG00000005507	G protein subunit alpha 14(GNA14)	-3,06
ENSOCUG00000017689	keratin 20(KRT20)	-3,06
ENSOCUG00000008329	ADAM metallopeptidase with thrombospondin type 1 motif 19(ADAMTS19)	-3,06
ENSOCUG00000008179	rhomboid like 3(RHBDL3)	-3,03
ENSOCUG00000015410	NDC80, kinetochore complex component(NDC80)	-3,03
ENSOCUG00000000215	period circadian clock 2(PER2)	-3,03
ENSOCUG00000006186	carboxypeptidase X, M14 family member 1(CPXM1)	-3,01
ENSOCUG00000009880	dual specificity phosphatase 14(DUSP14)	-3,00
ENSOCUG00000006613	spermatogenesis associated 1(SPATA1)	-2,99
ENSOCUG00000022883	glycerophosphodiester phosphodiesterase domain containing 3(GDPD3)	-2,99
ENSOCUG00000025494	secretogranin III(SCG3)	-2,98
ENSOCUG00000022659	EF-hand and coiled-coil domain containing 1(EFCC1)	-2,98
ENSOCUG00000006673	alpha 1,4-galactosyltransferase(A4GALT)	-2,97
ENSOCUG00000016942	aldehyde dehydrogenase 1 family member A2(ALDH1A2)	-2,95
ENSOCUG00000023005	zinc finger protein 367(ZNF367)	-2,93
ENSOCUG00000013801	SHC binding and spindle associated 1(SHCBP1)	-2,93
ENSOCUG00000026551	lysophosphatidic acid receptor 5(LPAR5)	-2,90
ENSOCUG00000004480	cysteine and glycine rich protein 3(CSRP3)	-2,89
ENSOCUG00000016718	mesenteric estrogen dependent adipogenesis(MEDAG)	-2,87
ENSOCUG00000026610	transmembrane protein 100(TMEM100)	-2,86
ENSOCUG00000010272	acid phosphatase, prostate(ACPP)	-2,86
ENSOCUG00000012902	glutathione S-transferase Yc(LOC100353428)	-2,85
ENSOCUG00000011042	collagen type VIII alpha 2 chain(COL8A2)	-2,85
ENSOCUG00000024465	collagen type VIII alpha 1 chain(COL8A1)	-2,80
ENSOCUG00000017859	RNA binding motif protein 46(RBM46)	-2,80
ENSOCUG00000017387	claspin(CLSPN)	-2,78
ENSOCUG00000011340	cell division cycle 20(CDC20)	-2,76
ENSOCUG00000015483	fatty acid binding protein 4(FABP4)	-2,75
ENSOCUG00000005747	collagen type XVI alpha 1 chain(COL16A1)	-2,74
ENSOCUG00000006962	aldehyde dehydrogenase 1 family member L2(ALDH1L2)	-2,73

ENSOCUG00000011027	chitinase 1(CHIT1)	-2,70
ENSOCUG00000011350	spondin 1(SPON1)	-2,66
ENSOCUG00000015838	collagen type IX alpha 2 chain(COL9A2)	-2,66
ENSOCUG00000004393	synaptotagmin-15(LOC100351528)	-2,63
ENSOCUG00000001627	solute carrier family 6 member 11(SLC6A11)	-2,62
ENSOCUG00000009332	mucolipin 3(MCOLN3)	-2,62
ENSOCUG00000015214	PSMC3 interacting protein(PSMC3IP)	-2,60
ENSOCUG00000007172	acyl-CoA desaturase(LOC100357419)	-2,59
ENSOCUG00000010941	carboxypeptidase X, M14 family member 2(CPXM2)	-2,58
ENSOCUG00000017197	claudin 6(CLDN6)	-2,57
ENSOCUG00000012176	ubiquitin conjugating enzyme E2 C(UBE2C)	-2,56
ENSOCUG00000017882	anoctamin 3(ANO3)	-2,56
ENSOCUG00000004340	cyclin B1(CCNB1)	-2,56
ENSOCUG00000026482	UDP-glucuronosyltransferase 2B16-like(LOC100340513)	-2,55
ENSOCUG00000000236	repulsive guidance molecule family member a(RGMA)	-2,54
ENSOCUG00000024196	F-box protein 17(FBXO17)	-2,54
ENSOCUG00000001337	prostate androgen-regulated mucin-like protein 1(PARM1)	-2,52
ENSOCUG00000029301	zinc finger and SCAN domain containing 23(ZSCAN23)	-2,52
ENSOCUG00000002173	phosphorylase, glycogen, muscle(PYGM)	-2,50
ENSOCUG00000006527	BUB1 mitotic checkpoint serine/threonine kinase B(BUB1B)	-2,50
ENSOCUG00000012264	collagen type I alpha 2 chain(COL1A2)	-2,49
ENSOCUG00000017043	cell division cycle associated 8(CDCA8)	-2,49
ENSOCUG00000003724	multiple EGF like domains 10(MEGF10)	-2,48
ENSOCUG00000009932	establishment of sister chromatid cohesion N-acetyltransferase 2(ESCO2)	-2,47
ENSOCUG00000014730	frizzled class receptor 2(FZD2)	-2,47
ENSOCUG00000005709	phosphoglucomutase 2 like 1(PGM2L1)	-2,46
ENSOCUG00000025992	butyrophilin subfamily 1 member A1-like(LOC100344369)	-2,46
ENSOCUG00000025655	G protein-coupled receptor 161(GPR161)	-2,45
ENSOCUG00000003822	family with sequence similarity 83 member B(FAM83B)	-2,44
ENSOCUG00000012683	ADAM metallopeptidase domain 33(ADAM33)	-2,44
ENSOCUG00000012071	glycerol-3-phosphate acyltransferase 3(GPAT3)	-2,44
ENSOCUG00000000094	prostacyclin synthase(LOC103346149)	-2,43
ENSOCUG00000002178	nurim (nuclear envelope membrane protein)(NRM)	-2,42
ENSOCUG00000003876	FH2 domain containing 1(FHDC1)	-2,41
ENSOCUG00000017273	patatin-like phospholipase domain-containing protein 3(LOC100344884)	-2,39
ENSOCUG00000012763	contactin-6(LOC100348097)	-2,37
ENSOCUG00000002878	SLX4 interacting protein(SLX4IP)	-2,36
ENSOCUG00000005592	lumican(LUM)	-2,35
ENSOCUG00000000580	cadherin 16(CDH16)	-2,35
ENSOCUG00000015162	microfibrillar associated protein 4(MFAP4)	-2,34
ENSOCUG00000000547	shugoshin 2(SGO2)	-2,33
ENSOCUG00000004511	heat shock protein family B (small) member 8(HSPB8)	-2,32
ENSOCUG00000002703	forkhead box M1(FOXM1)	-2,32
ENSOCUG00000010198	tropomyosin 2 (beta)(TPM2)	-2,30
ENSOCUG00000016111	ankyrin repeat and EF-hand domain containing 1(ANKEF1)	-2,27
ENSOCUG00000007516	6-phosphofructo-2-kinase/fructose-2,6-biphosphatase 3(PFKFB3)	-2,27
ENSOCUG00000005830	HIG1 hypoxia inducible domain family member 1A(HIGD1A)	-2,27
ENSOCUG00000005324	microtubule associated protein 1A(MAP1A)	-2,25

ENSOCUG00000002337	proline rich 11(PRR11)	-2,25
ENSOCUG00000003165	signal peptide, CUB and EGF-like domain-containing protein 1(LOC100344797)	-2,24
ENSOCUG00000011956	cadherin related family member 2(CDHR2)	-2,22
ENSOCUG00000012232	kin of IRRE like (Drosophila)(KIRREL)	-2,21
ENSOCUG00000016193	osteomodulin(OMD)	-2,21
ENSOCUG00000003030	PARP1 binding protein(PARPBP)	-2,20
ENSOCUG00000014571	SH3 and cysteine rich domain(STAC)	-2,19
ENSOCUG00000029066	transient receptor potential cation channel subfamily M member 2(LOC103347481)	-2,19
ENSOCUG00000005174	dermatopontin(DPT)	-2,19
ENSOCUG00000003689	nidogen 2(NID2)	-2,17
ENSOCUG00000015893	proprotein convertase subtilisin/kexin type 2(PCSK2)	-2,17
ENSOCUG00000014432	ectonucleotide pyrophosphatase/phosphodiesterase 3(ENPP3)	-2,17
ENSOCUG00000014988	collagen type III alpha 1 chain(COL3A1)	-2,16
ENSOCUG00000012275	testis expressed 9(TEX9)	-2,15
ENSOCUG00000009195	C-type lectin domain family 4 member E(CLEC4E)	-2,14
ENSOCUG00000002858	cyclin dependent kinase 1(CDK1)	-2,13
ENSOCUG00000004275	semaphorin 5A(SEMA5A)	-2,12
ENSOCUG00000001375	acyl-CoA desaturase(LOC100346046)	-2,11
ENSOCUG00000012809	solute carrier family 25 member 24(SLC25A24)	-2,11
ENSOCUG00000010936	chromosome unknown open reading frame, human C16orf89(LOC100353142)	-2,11
ENSOCUG00000003903	centromere protein U(CENPU)	-2,10
ENSOCUG00000000092	serpin family F member 1(SERPINF1)	-2,10
ENSOCUG00000009218	phosphodiesterase 10A(PDE10A)	-2,09
ENSOCUG00000029623	ATPase plasma membrane Ca2+ transporting 3(ATP2B3)	-2,07
ENSOCUG00000017153	transcription factor 19(TCF19)	-2,06
ENSOCUG00000001825	family with sequence similarity 171 member B(FAM171B)	-2,05
ENSOCUG00000011970	transgelin(TAGLN)	-2,04
ENSOCUG00000002707	MID1 interacting protein 1(MID1IP1)	-2,04
ENSOCUG00000013465	TBC1 domain family member 19(TBC1D19)	-2,04
ENSOCUG00000007735	FRAS1 related extracellular matrix protein 2(FREM2)	-2,03
ENSOCUG00000004485	E2F transcription factor 8(E2F8)	-2,02
ENSOCUG00000008953	synaptopodin 2(SYNPO2)	-2,02
ENSOCUG00000003254	cyclin dependent kinase like 2(CDKL2)	-2,02
ENSOCUG00000014056	dedicator of cytokinesis 5(DOCK5)	-2,02
ENSOCUG00000015836	myelin protein zero like 2(MPZL2)	-2,00
ENSOCUG00000022348	zinc finger and BTB domain containing 7C(ZBTB7C)	-2,00
ENSOCUG00000016302	alpha fetoprotein(AFP)	-2,00
ENSOCUG00000017492	centromere protein F(CENPF)	-1,99
ENSOCUG00000000409	collagen type VI alpha 2 chain(COL6A2)	-1,98
ENSOCUG00000000109	ADAM metallopeptidase with thrombospondin type 1 motif 9(ADAMTS9)	-1,97
ENSOCUG00000015790	kinesin family member 20A(KIF20A)	-1,97
ENSOCUG00000006973	adhesion G protein-coupled receptor E2(ADGRE2)	-1,97
ENSOCUG00000013450	myosin heavy chain 11(MYH11)	-1,97
ENSOCUG00000005503	spindle apparatus coiled-coil protein 1(SPDL1)	-1,96
ENSOCUG00000000847	protein tyrosine phosphatase, receptor type D(PTPRD)	-1,96
ENSOCUG00000000313	ovostatin homolog 2(LOC100348825)	-1,96
ENSOCUG00000013062	acyl-CoA synthetase long-chain family member 6(ACSL6)	-1,95
ENSOCUG00000016236	collagen type XV alpha 1 chain(COL15A1)	-1,94

ENSOCUG00000011244	extra spindle pole bodies like 1, separase(ESPL1)	-1,93
ENSOCUG00000015756	kinetochore scaffold 1(KNL1)	-1,91
ENSOCUG00000013757	solute carrier family 13 member 2(SLC13A2)	-1,90
ENSOCUG00000010184	trehalase(TREH)	-1,90
ENSOCUG00000017620	serum amyloid protein A(LOC100009259)	-1,89
ENSOCUG00000017867	Fraser extracellular matrix complex subunit 1(FRAS1)	-1,88
ENSOCUG00000012148	inositol 1,4,5-trisphosphate receptor type 1(ITPR1)	-1,88
ENSOCUG00000013414	RasGEF domain family member 1C(RASGEF1C)	-1,88
ENSOCUG00000025273	liver carboxylesterase 2(LOC100343300)	-1,87
ENSOCUG00000003277	nucleolar and spindle associated protein 1(NUSAP1)	-1,86
ENSOCUG00000005560	G protein subunit alpha transducin 2(GNAT2)	-1,84
ENSOCUG00000025241	liver carboxylesterase 2-like(LOC100357214)	-1,84
ENSOCUG00000016303	sulfotransferase family, cytosolic, 1C, member 2(SULT1C2)	-1,83
ENSOCUG00000024055	melanoma cell adhesion molecule(MCAM)	-1,83
ENSOCUG00000008571	myomesin 1(MYOM1)	-1,83
ENSOCUG00000008236	lipoprotein lipase(LPL)	-1,82
ENSOCUG00000025132	60S ribosomal protein L23a(LOC108177184)	-1,82
ENSOCUG00000005521	G protein-coupled bile acid receptor 1(GPBAR1)	-1,81
ENSOCUG00000006131	SPARC like 1(SPARCL1)	-1,81
ENSOCUG00000029071	zinc finger protein 81(ZNF81)	-1,80
ENSOCUG00000006714	matrix metallopeptidase 2(MMP2)	-1,80
ENSOCUG00000001785	syntaxin 19(STX19)	-1,78
ENSOCUG00000017585	family with sequence similarity 105 member A(FAM105A)	-1,78
ENSOCUG00000005885	immunoglobulin superfamily member 10(IGSF10)	-1,78
ENSOCUG00000005774	zinc finger protein 521(ZNF521)	-1,77
ENSOCUG00000002468	kinesin family member 20B(KIF20B)	-1,77
ENSOCUG00000002814	topoisomerase (DNA) II alpha(TOP2A)	-1,76
ENSOCUG00000005540	calpain 6(CAPN6)	-1,75
ENSOCUG00000012372	lipin 3(LPIN3)	-1,75
ENSOCUG00000016909	RAD51 recombinase(RAD51)	-1,75
ENSOCUG00000021287	heat shock protein family B (small) member 6(HSPB6)	-1,75
ENSOCUG00000023917	early B-cell factor 4(EBF4)	-1,74
ENSOCUG00000007545	EPH receptor B6(EPHB6)	-1,74
ENSOCUG00000005820	ribonucleotide reductase regulatory subunit M2(RRM2)	-1,73
ENSOCUG00000010046	thymidine kinase 1(TK1)	-1,73
ENSOCUG00000003203	TPX2, microtubule nucleation factor(TPX2)	-1,72
ENSOCUG00000001754	formin like 3(FMNL3)	-1,70
ENSOCUG00000013175	fibulin 5(FBLN5)	-1,70
ENSOCUG00000012031	pleckstrin 2(PLEK2)	-1,70
ENSOCUG00000014077	latent transforming growth factor beta binding protein 2(LTBP2)	-1,69
ENSOCUG00000000161	SH3 domain binding protein 2(SH3BP2)	-1,69
ENSOCUG00000017717	grainyhead like transcription factor 1(GRHL1)	-1,68
ENSOCUG00000016964	matrix Gla protein(MGP)	-1,68
ENSOCUG00000002874	protein tyrosine kinase 7 (inactive)(PTK7)	-1,68
ENSOCUG00000015111	glutamate ionotropic receptor NMDA type subunit 2B(GRIN2B)	-1,68
ENSOCUG00000015681	Rac GTPase activating protein 1(RACGAP1)	-1,67
ENSOCUG00000014083	leucine zipper tumor suppressor 1(LZTS1)	-1,67
ENSOCUG00000009017	osteoglycin(OGN)	-1,66

ENSOCUG00000023919	transgelin(LOC100009332)	-1,64
ENSOCUG00000001035	ADAM metallopeptidase with thrombospondin type 1 motif 2(ADAMTS2)	-1,64
ENSOCUG00000022434	cell division cycle associated 3(CDCA3)	-1,63
ENSOCUG00000009143	cyclin E1(CCNE1)	-1,63
ENSOCUG00000001863	vesicle amine transport 1 like(VAT1L)	-1,63
ENSOCUG00000010827	BOC cell adhesion associated, oncogene regulated(BOC)	-1,63
ENSOCUG00000010708	kinesin family member C1(KIFC1)	-1,63
ENSOCUG00000014801	acyl-CoA desaturase(LOC100346561)	-1,63
ENSOCUG00000024282	pleckstrin homology domain containing B2(PLEKHB2)	-1,62
ENSOCUG00000001389	leucine rich repeat and Ig domain containing 4(LINGO4)	-1,62
ENSOCUG00000022014	notch 3(NOTCH3)	-1,61
ENSOCUG00000006357	ADAM metallopeptidase with thrombospondin type 1 motif 12(ADAMTS12)	-1,61
ENSOCUG00000023213	pro-neuregulin-4, membrane-bound isoform-like(LOC108176558)	-1,61
ENSOCUG00000008491	testin LIM domain protein(TES)	-1,60
ENSOCUG00000010232	anthrax toxin receptor 1(ANTXR1)	-1,60
ENSOCUG00000021663	CUGBP, Elav-like family member 6(CELF6)	-1,60
ENSOCUG00000017377	matrilin 2(MATN2)	-1,60
ENSOCUG00000008818	creatine kinase B(CKB)	-1,60
ENSOCUG00000021411	tsukushi, small leucine rich proteoglycan(TSKU)	-1,60
ENSOCUG00000001801	forkhead box P2(FOXP2)	-1,59
ENSOCUG00000006490	leucine-rich repeat-containing protein 37A2(LOC103346478)	-1,58
ENSOCUG00000015020	collagen type V alpha 2 chain(COL5A2)	-1,57
ENSOCUG00000004137	protease, serine 23(PRSS23)	-1,55
ENSOCUG00000013868	MMS22 like, DNA repair protein(MMS22L)	-1,55
ENSOCUG00000002424	serum amyloid A-3(SAA3)	-1,54
ENSOCUG00000015443	adenylate cyclase 3(ADCY3)	-1,54
ENSOCUG00000023778	CD300a molecule(CD300A)	-1,53
ENSOCUG00000017136	solute carrier family 27 member 6(SLC27A6)	-1,53
ENSOCUG00000016347	phosphoethanolamine/phosphocholine phosphatase(PHOSPHO1)	-1,53
ENSOCUG00000001921	KIAA0825 ortholog(KIAA0825)	-1,52
ENSOCUG00000024273	sulfatase 2(SULF2)	-1,52
ENSOCUG00000027359	tetraspanin 11(TSPAN11)	-1,52
ENSOCUG00000013478	HtrA serine peptidase 1(HTRA1)	-1,51
ENSOCUG00000003866	fibrillin 1(FBN1)	-1,51
ENSOCUG00000017019	cadherin 11(CDH11)	-1,51
ENSOCUG00000008498	uridine phosphorylase 2(UPP2)	-1,49
ENSOCUG00000008841	glutamate-ammonia ligase(GLUL)	-1,49
ENSOCUG00000027031	zinc finger protein 496(ZNF496)	-1,49
ENSOCUG00000015371	serine peptidase inhibitor, Kunitz type 1(SPINT1)	-1,49
ENSOCUG00000009543	potassium calcium-activated channel subfamily M alpha 1(KCNMA1)	-1,48
ENSOCUG00000010299	cysteine and glycine rich protein 1(CSRP1)	-1,48
ENSOCUG00000015039	fibronectin type III domain containing 1(FNDC1)	-1,47
ENSOCUG00000015853	glutaredoxin(GLRX)	-1,46
ENSOCUG00000012866	TEF, PAR bZIP transcription factor(TEF)	-1,45
ENSOCUG00000003663	actin, gamma 2, smooth muscle, enteric(ACTG2)	-1,44
ENSOCUG00000008855	cerebral endothelial cell adhesion molecule(CERCAM)	-1,44
ENSOCUG00000008012	stathmin(LOC100358241)	-1,43
ENSOCUG00000008476	polo like kinase 1(PLK1)	-1,42

ENSOCUG00000024939	interferon induced protein with tetratricopeptide repeats 2(IFIT2)	-1,42
ENSOCUG00000022047	MPV17 mitochondrial inner membrane protein like(MPV17L)	-1,42
ENSOCUG00000017494	mannose receptor C type 2(MRC2)	-1,42
ENSOCUG00000026406	butyrophilin subfamily 1 member A1-like(LOC108175832)	-1,41
ENSOCUG00000027184	carbonyl reductase 1(CBR1)	-1,41
ENSOCUG00000016815	islet cell autoantigen 1(ICA1)	-1,40
ENSOCUG00000007759	ubiquitin specific peptidase 2(USP2)	-1,39
ENSOCUG00000005896	inositol polyphosphate-4-phosphatase type II B(INPP4B)	-1,39
ENSOCUG00000005317	calcium release activated channel regulator 2A(CRACR2A)	-1,39
ENSOCUG00000001912	plexin B1(PLXNB1)	-1,38
ENSOCUG00000011626	calcium/calmodulin dependent protein kinase ID(CAMK1D)	-1,38
ENSOCUG00000004590	collagen type XXVII alpha 1 chain(COL27A1)	-1,37
ENSOCUG00000003858	glycine N-methyltransferase(GNMT)	-1,36
ENSOCUG00000026099	peripheral myelin protein 22(PMP22)	-1,36
ENSOCUG00000000637	myosin light chain 9(MYL9)	-1,35
ENSOCUG00000011126	heparanase(HPSE)	-1,35
ENSOCUG00000008478	syntaxin binding protein 4(STXBP4)	-1,35
ENSOCUG00000005900	fibulin 2(FBLN2)	-1,35
ENSOCUG00000006917	ER membrane associated RNA degradation(ERMARD)	-1,35
ENSOCUG00000004505	coiled-coil domain containing 77(CCDC77)	-1,34
ENSOCUG00000014596	lysyl oxidase like 2(LOXL2)	-1,34
ENSOCUG00000016538	minichromosome maintenance complex component 6(MCM6)	-1,33
ENSOCUG00000012971	GLIS family zinc finger 2(GLIS2)	-1,33
ENSOCUG00000010637	minichromosome maintenance complex component 5(MCM5)	-1,33
ENSOCUG00000026731	helicase, lymphoid-specific(HELLS)	-1,33
ENSOCUG00000015372	DEAQ-box RNA dependent ATPase 1(DQX1)	-1,33
ENSOCUG00000004841	dickkopf WNT signaling pathway inhibitor 3(DKK3)	-1,33
ENSOCUG00000002376	HAUS augmin like complex subunit 7(HAUS7)	-1,33
ENSOCUG00000026480	aldehyde oxidase 2(AOX2)	-1,32
ENSOCUG00000015509	non-SMC condensin II complex subunit G2(NCAPG2)	-1,32
ENSOCUG00000010244	matrix metallopeptidase 14(MMP14)	-1,31
ENSOCUG00000013696	BicC family RNA binding protein 1(BICC1)	-1,31
ENSOCUG00000015924	minichromosome maintenance complex component 2(MCM2)	-1,30
ENSOCUG00000016164	cytochrome P450 1B1(LOC100358590)	-1,30
ENSOCUG00000005032	family with sequence similarity 169 member B(FAM169B)	-1,29
ENSOCUG00000006350	solute carrier family 12 member 4(SLC12A4)	-1,29
ENSOCUG00000007526	cystathionine gamma-lyase(CTH)	-1,28
ENSOCUG00000000560	hes related family bHLH transcription factor with YRPW motif-like(HEYL)	-1,27
ENSOCUG00000003212	tumor suppressor candidate 3(TUSC3)	-1,27
ENSOCUG00000006493	stathmin 1(STMN1)	-1,27
ENSOCUG00000006927	Rho GTPase activating protein 42(ARHGAP42)	-1,26
ENSOCUG00000014326	prostaglandin-E(2) 9-reductase-like(LOC100352716)	-1,26
ENSOCUG00000007967	four and a half LIM domains 2(FHL2)	-1,26
ENSOCUG00000024509	butyrophilin subfamily 1 member A1(LOC100343656)	-1,26
ENSOCUG00000016717	INTS3 and NABP interacting protein(INIP)	-1,24
ENSOCUG00000014029	SET domain containing 4(SETD4)	-1,24
ENSOCUG00000013643	PDZ and LIM domain 1(PDLIM1)	-1,24
ENSOCUG00000003091	adenylate cyclase 7(ADCY7)	-1,23

ENSOCUG00000015051	protein phosphatase 1 regulatory subunit 3C(PPP1R3C)	-1,21
ENSOCUG00000005474	sarcoglycan beta(SGCB)	-1,21
ENSOCUG00000001673	triokinase and FMN cyclase(TKFC)	-1,21
ENSOCUG00000006969	decorin(DCN)	-1,21
ENSOCUG00000009260	phospholipase C eta 1(PLCH1)	-1,21
ENSOCUG00000002336	bone marrow stromal cell antigen 1(BST1)	-1,21
ENSOCUG00000013609	lipopolysaccharide binding protein(LBP)	-1,20
ENSOCUG00000014498	solute carrier family 16 member 12(SLC16A12)	-1,19
ENSOCUG00000015234	collagen type XIV alpha 1 chain(COL14A1)	-1,17
ENSOCUG00000000324	proline-serine-threonine phosphatase interacting protein 2(PSTPIP2)	1,39
ENSOCUG00000006009	acyl-coenzyme A thioesterase 1(LOC100344509)	1,43
ENSOCUG00000029130	cytochrome P450 2C1(CYP2C1)	1,46
ENSOCUG00000007555	peptidoglycan recognition protein 2(PGLYRP2)	1,46
ENSOCUG00000007699	kynurenine 3-monooxygenase(KMO)	1,47
ENSOCUG00000022168	serine dehydratase(SDS)	1,51
ENSOCUG00000015313	cytochrome c oxidase protein 20 homolog(LOC100349428)	1,52
ENSOCUG00000014197	macrophage scavenger receptor 1(MSR1)	1,55
ENSOCUG00000016959	absent in melanoma 1-like(AIM1L)	1,56
ENSOCUG00000003246	sulfatase 1(SULF1)	1,58
ENSOCUG00000026526	protein phosphatase, Mg2+/Mn2+ dependent 1E(PPM1E)	1,59
ENSOCUG00000007130	solute carrier family 25 member 25(SLC25A25)	1,60
ENSOCUG00000009725	acyl-CoA wax alcohol acyltransferase 1(AWAT1)	1,61
ENSOCUG00000027233	uncharacterized LOC100346308(LOC100346308)	1,64
ENSOCUG00000006937	coiled-coil domain containing 57(CCDC57)	1,67
ENSOCUG00000004163	solute carrier family 25 member 33(SLC25A33)	1,67
ENSOCUG00000011634	sphingomyelin phosphodiesterase 3(SMPD3)	1,70
ENSOCUG00000001028	major facilitator superfamily domain containing 2A(MFSD2A)	1,75
ENSOCUG00000016068	serpin family E member 1(SERPINE1)	1,75
ENSOCUG00000001185	G protein-coupled receptor 149(GPR149)	1,81
ENSOCUG00000024272	nexilin F-actin binding protein(NEXN)	1,82
ENSOCUG00000011201	CYP4B1-like isozyme short form(CYP4B1)	1,84
ENSOCUG00000007999	dual specificity phosphatase 1(DUSP1)	1,84
ENSOCUG00000021506	Fos proto-oncogene, AP-1 transcription factor subunit(FOS)	1,87
ENSOCUG00000024078	protein GREB1(LOC100345224)	1,89
ENSOCUG00000025236	leukocyte protein(LOC100009166)	1,91
ENSOCUG00000024474	L-gulonolactone oxidase(LOC100341843)	1,91
ENSOCUG00000002702	radical S-adenosyl methionine domain containing 1(RSAD1)	1,92
ENSOCUG00000023743	heat shock 70 kDa protein 1B(LOC100354435)	1,92
ENSOCUG00000002000	sarcoglycan delta(SGCD)	1,93
ENSOCUG00000004662	Tctex1 domain containing 1(TCTEX1D1)	2,02
ENSOCUG00000014749	ATP/GTP binding protein like 3(AGBL3)	2,06
ENSOCUG00000017424	activating transcription factor 3(ATF3)	2,09
ENSOCUG00000005465	nocturnin(NOCT)	2,10
ENSOCUG00000013296	growth arrest and DNA damage inducible gamma(GADD45G)	2,12
ENSOCUG00000029339	acyl-CoA wax alcohol acyltransferase 2(AWAT2)	2,12
ENSOCUG00000004099	ganglioside induced differentiation associated protein 1 like 1(GDAP1L1)	2,15
ENSOCUG00000004492	potassium voltage-gated channel subfamily Q member 1(KCNQ1)	2,18
ENSOCUG00000009863	protein kinase C theta(PRKCQ)	2,21

ENSOCUG00000002656	trafficking protein particle complex 3 like(TRAPPC3L)	2,26
ENSOCUG00000004269	transient receptor potential cation channel subfamily V member 4(TRPV4)	2,26
ENSOCUG00000006499	mitochondria localized glutamic acid rich protein(MGARP)	2,29
ENSOCUG00000012452	adhesion G protein-coupled receptor F3(ADGRF3)	2,32
ENSOCUG00000012831	insulin like growth factor binding protein 1(IGFBP1)	2,39
ENSOCUG00000008253	tissue factor pathway inhibitor(TFPI)	2,46
ENSOCUG00000025112	heat shock 70 kDa protein 1B(LOC100354037)	2,48
ENSOCUG00000002457	canopy FGF signaling regulator 1(CNPY1)	2,49
ENSOCUG00000015664	tryptophan hydroxylase 2(TPH2)	2,57
ENSOCUG00000005480	TATA-box binding protein associated factor 4b(TAF4B)	2,68
ENSOCUG00000023871	uncharacterized LOC100347087(LOC100347087)	2,78
ENSOCUG00000017102	serpin family A member 7(SERPINA7)	2,81
ENSOCUG00000023547	tubulin alpha-3 chain(LOC100350967)	2,85
ENSOCUG00000007038	urotensin 2B(UTS2B)	2,92
ENSOCUG00000000784	nuclear receptor subfamily 4 group A member 2(NR4A2)	2,99
ENSOCUG00000027771	zymogen granule membrane protein 16-like(LOC100359023)	3,02
ENSOCUG00000023796	lactotransferrin(LTF)	3,03
ENSOCUG00000006597	calmegin(CLGN)	3,14
ENSOCUG00000008182	coiled-coil domain containing 136(CCDC136)	3,24
ENSOCUG00000012939	ectonucleotide pyrophosphatase/phosphodiesterase 5 (putative)(ENPP5)	3,27
ENSOCUG00000002174	testis expressed 12(TEX12)	3,39
ENSOCUG00000029634	arachidonate lipoxygenase 3(ALOXE3)	3,77
ENSOCUG00000022232	zymogen granule membrane protein 16(LOC100345057)	4,89
ENSOCUG00000027212	zymogen granule membrane protein 16-like(LOC100339377)	6,91
ENSOCUG00000017680	family with sequence similarity 212 member B(FAM212B)	< -0,01
ENSOCUG00000008748	5'-nucleotidase domain containing 4(NT5DC4)	< -0,01
ENSOCUG00000010366	transmembrane protease, serine 13(TMPRSS13)	< -0,01
ENSOCUG00000016046	potassium voltage-gated channel subfamily A member 6(KCNA6)	< -0,01
ENSOCUG00000001255	RRAD and GEM like GTPase 2(REM2)	< -0,02
ENSOCUG00000002319	PCNA-associated factor-like(LOC100356881)	< -0,02
ENSOCUG00000008557	adenylate cyclase 2(ADCY2)	< -0,02
ENSOCUG00000002283	family with sequence similarity 84 member A(FAM84A)	< -0,03
ENSOCUG00000003999	calcium/calmodulin dependent protein kinase IG(CAMK1G)	< -0,03
ENSOCUG00000005596	gremlin 1, DAN family BMP antagonist(GREM1)	< -0,03
ENSOCUG00000010335	DS cell adhesion molecule like 1(DSCAML1)	< -0,03
ENSOCUG00000015215	homeobox A1(HOXA1)	< -0,03
ENSOCUG00000016650	DEP domain containing 1B(DEPDC1B)	< -0,03
ENSOCUG00000001041	maternal embryonic leucine zipper kinase(MELK)	< -0,04
ENSOCUG00000006091	odd-skipped related transciption factor 1(OSR1)	< -0,04
ENSOCUG00000006437	RUN domain containing 3A(RUNDC3A)	< -0,04
ENSOCUG00000007225	cellular retinoic acid binding protein 2(CRABP2)	< -0,04
ENSOCUG00000002624	Nik related kinase(NRK)	< -0,05
ENSOCUG00000006383	ghrelin and obestatin prepropeptide(GHRL)	< -0,05
ENSOCUG00000006801	minichromosome maintenance 10 replication initiation factor(MCM10)	< -0,05
ENSOCUG00000004047	spindle and kinetochore associated complex subunit 3(SKA3)	< -0,07
ENSOCUG00000006341	calbindin 1(CALB1)	< -0,07
ENSOCUG00000007263	cell surface glycoprotein CD200 receptor 2(LOC100348666)	< -0,08
ENSOCUG00000014335	tripartite motif family like 1(TRIML1)	< -0,08

ENSOCUG00000014421	myelin protein zero(MPZ)	< -0,09
ENSOCUG00000002956	copine 4(CPNE4)	< -0,10
ENSOCUG00000007994	HECT, C2 and WW domain containing E3 ubiquitin protein ligase 1(HECW1)	< -0,11
ENSOCUG00000008418	beta-1,4-N-acetyl-galactosaminyltransferase 3(B4GALNT3)	< -0,11
ENSOCUG00000013934	coiled-coil domain containing 189(CCDC189)	< -0,11
ENSOCUG00000012527	solute carrier family 26 member 7(SLC26A7)	< -0,12
ENSOCUG00000014857	collagen type V alpha 3 chain(COL5A3)	< -0,12
ENSOCUG00000000266	Wnt family member 2B(WNT2B)	< -0,13
ENSOCUG00000001961	RAD51 associated protein 1(RAD51AP1)	< -0,13
ENSOCUG00000008531	hydroxysteroid 17-beta dehydrogenase 3(HSD17B3)	< -0,13
ENSOCUG00000009528	intestinal-type alkaline phosphatase-like(LOC100352107)	< -0,13
ENSOCUG00000027644	translocator protein 2(TSPO2)	< -0,13
ENSOCUG00000017309	5-hydroxytryptamine receptor 4(HTR4)	< -0,15
ENSOCUG00000010677	insulin receptor related receptor(INSRR)	< -0,16
ENSOCUG00000011298	family with sequence similarity 83 member D(FAM83D)	< -0,16
ENSOCUG00000004089	matrix metallopeptidase 28(MMP28)	< -0,17
ENSOCUG00000016277	epiregulin(EREG)	< -0,19
ENSOCUG00000007146	1-aminocyclopropane-1-carboxylate synthase homolog (inactive) like(ACCSL)	< -0,22
ENSOCUG00000029599	killer cell lectin like receptor B1(KLRB1)	< -0,24
ENSOCUG00000006792	non-SMC condensin I complex subunit G(NCAPG)	< -0,32
ENSOCUG00000016390	WNT1 inducible signaling pathway protein 1(WISP1)	< -0,40
ENSOCUG00000029014	chromosome 13 open reading frame, human C1orf105(C13H1orf105)	< -0,41
ENSOCUG00000003120	opioid receptor kappa 1(OPRK1)	< -0,47
ENSOCUG00000014832	STEAP2 metalloreductase(STEAP2)	< -0,56
ENSOCUG00000026203	complement C1q like 1(C1QL1)	< -1,17
ENSOCUG00000002462	solute carrier family 24 member 2(SLC24A2)	< -2,13
ENSOCUG00000026293	leucine rich repeats and transmembrane domains 1(LRTM1)	< -3,82
ENSOCUG00000000087	TLC domain containing 2(TLCD2)	> 0,02
ENSOCUG00000010077	potassium channel tetramerization domain containing 19(KCTD19)	> 0,02
ENSOCUG00000007327	contactin associated protein 1(CNTNAP1)	> 0,03
ENSOCUG00000009777	GRB2 associated regulator of MAPK1 subtype 2(GAREM2)	> 0,03
ENSOCUG00000016833	chromosome 13 open reading frame, human C1orf228(C13H1orf228)	> 0,03
ENSOCUG00000022998	tubulin alpha-3 chain(LOC100349209)	> 0,03
ENSOCUG00000004632	carbonic anhydrase 12(CA12)	> 0,04
ENSOCUG00000006025	proopiomelanocortin(POMC)	> 0,04
ENSOCUG00000013544	ATPase H+ transporting V0 subunit a4(ATP6V0A4)	> 0,04
ENSOCUG00000003926	transmembrane protein 89(TMEM89)	> 0,05
ENSOCUG00000004187	paired box 8(PAX8)	> 0,05
ENSOCUG00000007102	tubulin polymerization promoting protein family member 2(TPPP2)	> 0,05
ENSOCUG00000009532	AF4/FMR2 family member 3(AFF3)	> 0,05
ENSOCUG00000011656	actin like 7A(ACTL7A)	> 0,05
ENSOCUG00000024091	solute carrier family 22 member 8(SLC22A8)	> 0,05
ENSOCUG00000003274	Opa interacting protein 5(OIP5)	> 0,06
ENSOCUG00000015293	porcupine homolog (Drosophila)(PORCN)	> 0,06
ENSOCUG00000016063	CKLF like MARVEL transmembrane domain containing 2(CMTM2)	> 0,06
ENSOCUG00000029056	C-type lectin domain family 17 member A(CLEC17A)	> 0,06
ENSOCUG00000000197	hexokinase 2(HK2)	> 0,07
ENSOCUG00000001207	estrogen related receptor beta(ESRRB)	> 0,07

ENSOCUG00000022774	cation channel sperm associated auxiliary subunit gamma(CATSPERG)	> 0,07
ENSOCUG00000001822	kinesin family member 17(KIF17)	> 0,08
ENSOCUG00000010922	glyceraldehyde-3-phosphate dehydrogenase, spermatogenic(GAPDHS)	> 0,08
ENSOCUG00000013226	BLK proto-oncogene, Src family tyrosine kinase(BLK)	> 0,08
ENSOCUG00000005547	chromosome 19 open reading frame, human C17orf74(C19H17orf74)	> 0,09
ENSOCUG00000013299	heat shock protein family B (small) member 9(HSPB9)	> 0,09
ENSOCUG00000016389	solute carrier family 17 member 6(SLC17A6)	> 0,10
ENSOCUG00000016652	RIB43A domain with coiled-coils 2(RIBC2)	> 0,10
ENSOCUG00000023766	disintegrin and metalloproteinase domain-containing protein 18(LOC100358989)	> 0,10
ENSOCUG00000005270	phosphoglycerate kinase 2(PGK2)	> 0,11
ENSOCUG00000027853	phosphoglycerate mutase 2(PGAM2)	> 0,11
ENSOCUG00000029412	zymogen granule membrane protein 16-like(LOC100352055)	> 0,11
ENSOCUG00000008107	maestro heat like repeat family member 7(MROH7)	> 0,12
ENSOCUG00000015787	beaded filament structural protein 1(BFSP1)	> 0,12
ENSOCUG00000010163	acyl-CoA synthetase bubblegum family member 2(ACSBG2)	> 0,13
ENSOCUG00000022427	myomegalin(LOC103350070)	> 0,14
ENSOCUG00000003737	acrosin binding protein(ACRBP)	> 0,15
ENSOCUG00000027844	solute carrier family 24 member 1(SLC24A1)	> 0,16
ENSOCUG00000000119	solute carrier family 51 alpha subunit(SLC51A)	> 0,17
ENSOCUG00000021920	neuraminidase 4(NEU4)	> 0,18
ENSOCUG00000017222	outer dense fiber of sperm tails 1(ODF1)	> 0,19
ENSOCUG00000015667	SH3 domain containing ring finger 2(SH3RF2)	> 0,20
ENSOCUG00000004280	chromosome 4 open reading frame, human C12orf50(C4H12orf50)	> 0,24
ENSOCUG00000022253	putative spermatogenesis-associated protein 31D3(LOC100341232)	> 0,24
ENSOCUG00000005090	spermatogenic leucine zipper 1(SPZ1)	> 0,26
ENSOCUG00000029604	calcium binding protein, spermatid associated 1(CABS1)	> 0,26
ENSOCUG00000027755	putative spermatogenesis-associated protein 31D3(LOC100355671)	> 0,29
ENSOCUG00000017123	cilia and flagella associated protein 53(CFAP53)	> 0,30
ENSOCUG00000006562	LanC like 3(LANCL3)	> 0,32
ENSOCUG00000001385	ornithine decarboxylase antizyme 3(OAZ3)	> 0,35
ENSOCUG00000010340	arachidonate 15-lipoxygenase, type B(ALOX15B)	> 0,41
ENSOCUG00000015803	tubulointerstitial nephritis antigen(TINAG)	> 0,45
ENSOCUG00000012837	germ cell associated 1(GSG1)	> 0,46
ENSOCUG00000021104	transition protein 2(TNP2)	> 0,47
ENSOCUG00000005537	radial spoke head 9 homolog(RSPH9)	> 0,52
ENSOCUG00000015841	solute carrier family 36 member 3(SLC36A3)	> 0,72
ENSOCUG00000026150	CD19 molecule(CD19)	> 1,04
ENSOCUG00000025309	glycerol kinase 2(GK2)	> 1,60
ENSOCUG00000026777	zymogen granule membrane protein 16(LOC100346433)	> 104,
ENSOCUG00000029690	zymogen granule membrane protein 16(LOC100350057)	> 15,6
ENSOCUG00000027492	zymogen granule membrane protein 16(LOC100346271)	> 18,8
ENSOCUG00000025809	reticulon 4 receptor-like 2(RTN4RL2)	> 2,37
ENSOCUG00000025107	membrane associated ring-CH-type finger 4(MARCH4)	> 4,13
ENSOCUG00000026477	tubulin alpha-3 chain(LOC100350027)	> 7,13

Supplementary Table S5. Functional analysis of differential expressed transcripts in liver tissue between animals born from vitrified-transferred embryos and those conceived naturally.

Category*	Term	Count	p-value
BP	collagen fibril organization	9	0,000
BP	mitotic cytokinesis	7	0,000
BP	extracellular fibril organization	4	0,000
BP	negative regulation of growth	4	0,004
BP	elastic fiber assembly	3	0,007
BP	DNA replication initiation	4	0,007
BP	regulation of cytoskeleton organization	3	0,011
BP	cellular response to zinc ion	3	0,022
BP	DNA unwinding involved in DNA replication	3	0,022
BP	glomerular filtration	3	0,022
BP	protein localization to kinetochore	3	0,022
BP	microtubule-based movement	6	0,023
BP	fatty acid biosynthetic process	4	0,027
BP	cell adhesion	9	0,028
BP	response to hormone	3	0,029
BP	positive regulation of vascular endothelial growth factor production	4	0,031
BP	glycolytic process	4	0,035
BP	positive regulation of macrophage derived foam cell differentiation	3	0,037
BP	cellular response to amino acid stimulus	5	0,042
BP	arachidonic acid metabolic process	3	0,045
BP	skin development	4	0,049
BP	endodermal cell differentiation	4	0,054
BP	mitotic metaphase plate congression	4	0,060
BP	positive regulation of branching involved in ureteric bud morphogenesis	3	0,063
BP	heparan sulfate proteoglycan metabolic process	2	0,068
BP	glial cell-derived neurotrophic factor receptor signaling pathway	2	0,068
BP	esophagus smooth muscle contraction	2	0,068
BP	positive regulation of apoptotic process	8	0,073
BP	collagen catabolic process	3	0,073
BP	artery morphogenesis	3	0,073
BP	acute-phase response	3	0,083
BP	canonical Wnt signaling pathway	5	0,084
BP	cell-matrix adhesion	5	0,084
BP	endothelial cell migration	3	0,094
CC	extracellular matrix	19	0,000
CC	proteinaceous extracellular matrix	20	0,000
CC	extracellular space	47	0,000
CC	zymogen granule membrane	7	0,000
CC	collagen trimer	7	0,000
CC	extracellular exosome	87	0,000
CC	basement membrane	7	0,002
CC	MCM complex	4	0,002

CC	midbody	9	0,005
CC	condensed nuclear chromosome outer kinetochore	3	0,006
CC	spindle microtubule	5	0,010
CC	microfibril	3	0,014
CC	cell surface	17	0,014
CC	microtubule	8	0,039
CC	chromocenter	3	0,046
CC	cytoplasm	75	0,050
CC	fibrillar collagen trimer	2	0,063
CC	Ndc80 complex	2	0,063
CC	elastic fiber	2	0,063
CC	collagen type V trimer	2	0,063
CC	chromatin	5	0,071
MF	calcium ion binding	30	0,000
MF	extracellular matrix structural constituent	6	0,001
MF	metalloendopeptidase activity	11	0,002
MF	oxidoreductase activity, acting on paired donors, with oxidation of a pair of donors resulting in the reduction of molecular oxygen to two molecules of water	3	0,003
MF	carbohydrate binding	8	0,005
MF	scavenger receptor activity	5	0,021
MF	double-stranded DNA binding	4	0,022
MF	adenylate cyclase activity	3	0,029
MF	long-chain fatty acid-CoA ligase activity	3	0,043
MF	structural constituent of muscle	3	0,058
MF	long-chain-alcohol O-fatty-acyltransferase activity	2	0,060
MF	microtubule motor activity	5	0,066
MF	zinc ion binding	34	0,072
MF	arylsulfatase activity	2	0,089
MF	N-acetylglucosamine-6-sulfatase activity	2	0,089
KEGG	Protein digestion and absorption	12	0,000
KEGG	Cell cycle	13	0,000
KEGG	PPAR signaling pathway	9	0,000
KEGG	Platelet activation	10	0,005
KEGG	Oocyte meiosis	9	0,005
KEGG	Gap junction	8	0,005
KEGG	Circadian entrainment	8	0,006
KEGG	Starch and sucrose metabolism	5	0,009
KEGG	Estrogen signaling pathway	8	0,009
KEGG	Glycerolipid metabolism	7	0,010
KEGG	p53 signaling pathway	7	0,010
KEGG	Gastric acid secretion	7	0,010
KEGG	Aldosterone synthesis and secretion	7	0,012
KEGG	Metabolic pathways	45	0,016
KEGG	Salivary secretion	7	0,019
KEGG	Mineral absorption	5	0,020
KEGG	Progesterone-mediated oocyte maturation	7	0,022
KEGG	ECM-receptor interaction	7	0,022

KEGG	Pancreatic secretion	8	0,026
KEGG	Melanogenesis	7	0,032
KEGG	Bile secretion	6	0,032
KEGG	Vascular smooth muscle contraction	8	0,033
KEGG	Oxytocin signaling pathway	9	0,034
KEGG	Biosynthesis of unsaturated fatty acids	4	0,045
KEGG	Fatty acid metabolism	5	0,051
KEGG	Glutamatergic synapse	7	0,052
KEGG	Amoebiasis	7	0,056
KEGG	cAMP signaling pathway	10	0,058
KEGG	GnRH signaling pathway	6	0,059
KEGG	Drug metabolism - other enzymes	5	0,083
KEGG	Retrograde endocannabinoid signaling	6	0,087
KEGG	Dilated cardiomyopathy	6	0,087
KEGG	Adipocytokine signaling pathway	5	0,087
KEGG	Thyroid hormone synthesis	5	0,094
KEGG	Cholinergic synapse	6	0,096

*Functional analysis was referred to the GO term annotation according to the biological process (BP), cellular component (CC) and molecular function (MF) classification, and the KEGG pathways in which they are involved.

Supplementary Table S6. Differentially expressed transcripts of vitrified-transferred animals compared to those naturally-conceived in F1, which are inherited by the vitrified-transferred progeny in the F2 and F3 generations.

DETs inherited by F2	DETs inherited by F3
ENSOCUG00000000109	ENSOCUG00000000092
ENSOCUG00000000197	ENSOCUG00000000215
ENSOCUG00000000215	ENSOCUG00000000266
ENSOCUG00000000313	ENSOCUG00000000580
ENSOCUG00000000580	ENSOCUG00000001185
ENSOCUG00000000856	ENSOCUG00000001754
ENSOCUG00000001171	ENSOCUG00000002462
ENSOCUG00000001375	ENSOCUG00000002542
ENSOCUG00000001376	ENSOCUG00000002624
ENSOCUG00000001627	ENSOCUG00000002702
ENSOCUG00000001652	ENSOCUG00000002956
ENSOCUG00000001673	ENSOCUG00000003030
ENSOCUG00000001863	ENSOCUG00000003091
ENSOCUG00000002000	ENSOCUG00000003246
ENSOCUG00000002703	ENSOCUG00000003724
ENSOCUG00000002707	ENSOCUG00000003858
ENSOCUG00000002814	ENSOCUG00000004004
ENSOCUG00000002858	ENSOCUG00000004047
ENSOCUG00000002878	ENSOCUG00000004275
ENSOCUG00000003203	ENSOCUG00000004343
ENSOCUG00000003217	ENSOCUG00000004480
ENSOCUG00000003246	ENSOCUG00000004632
ENSOCUG00000003467	ENSOCUG00000005159
ENSOCUG00000003649	ENSOCUG00000005521
ENSOCUG00000003876	ENSOCUG00000005830
ENSOCUG00000004393	ENSOCUG00000005885
ENSOCUG00000004485	ENSOCUG00000006350
ENSOCUG00000004492	ENSOCUG00000006597
ENSOCUG00000004632	ENSOCUG00000007151
ENSOCUG00000005032	ENSOCUG00000007327
ENSOCUG00000005159	ENSOCUG00000007759
ENSOCUG00000005465	ENSOCUG00000008236
ENSOCUG00000005540	ENSOCUG00000008303
ENSOCUG00000005820	ENSOCUG00000008329
ENSOCUG00000005900	ENSOCUG00000008571
ENSOCUG00000006009	ENSOCUG00000009195

ENSOCUG00000006357	ENSOCUG00000009332
ENSOCUG00000006490	ENSOCUG00000009528
ENSOCUG00000006801	ENSOCUG00000009532
ENSOCUG00000006962	ENSOCUG00000009543
ENSOCUG00000007172	ENSOCUG00000009725
ENSOCUG00000007516	ENSOCUG00000009932
ENSOCUG00000007555	ENSOCUG00000009993
ENSOCUG00000007759	ENSOCUG00000010144
ENSOCUG00000008179	ENSOCUG00000010184
ENSOCUG00000008329	ENSOCUG00000010637
ENSOCUG00000008478	ENSOCUG00000010814
ENSOCUG00000008841	ENSOCUG00000010941
ENSOCUG00000009260	ENSOCUG00000011025
ENSOCUG00000009725	ENSOCUG00000011195
ENSOCUG00000009880	ENSOCUG00000011350
ENSOCUG00000009993	ENSOCUG00000011488
ENSOCUG00000010299	ENSOCUG00000011956
ENSOCUG00000010708	ENSOCUG00000012148
ENSOCUG00000010814	ENSOCUG00000012372
ENSOCUG00000010975	ENSOCUG00000012902
ENSOCUG00000011025	ENSOCUG00000012939
ENSOCUG00000011201	ENSOCUG00000013140
ENSOCUG00000011298	ENSOCUG00000013226
ENSOCUG00000011340	ENSOCUG00000013412
ENSOCUG00000011488	ENSOCUG00000013609
ENSOCUG00000011634	ENSOCUG00000013757
ENSOCUG00000011656	ENSOCUG00000013934
ENSOCUG00000012071	ENSOCUG00000014077
ENSOCUG00000012148	ENSOCUG00000014204
ENSOCUG00000012452	ENSOCUG00000014498
ENSOCUG00000012831	ENSOCUG00000014857
ENSOCUG00000012902	ENSOCUG00000015111
ENSOCUG00000013074	ENSOCUG00000015313
ENSOCUG00000013111	ENSOCUG00000015329
ENSOCUG00000013160	ENSOCUG00000015483
ENSOCUG00000013414	ENSOCUG00000015664
ENSOCUG00000013478	ENSOCUG00000015787
ENSOCUG00000013544	ENSOCUG00000015803
ENSOCUG00000013643	ENSOCUG00000015893
ENSOCUG00000014077	ENSOCUG00000016193

ENSOCUG00000014801	ENSOCUG00000016347
ENSOCUG00000015483	ENSOCUG00000017347
ENSOCUG00000015664	ENSOCUG00000017620
ENSOCUG00000015667	ENSOCUG00000017803
ENSOCUG00000015681	ENSOCUG00000020947
ENSOCUG00000015803	ENSOCUG00000021508
ENSOCUG00000015924	ENSOCUG00000022280
ENSOCUG00000016303	ENSOCUG00000022308
ENSOCUG00000016347	ENSOCUG00000022392
ENSOCUG00000016496	ENSOCUG00000022543
ENSOCUG00000016718	ENSOCUG00000022883
ENSOCUG00000016815	ENSOCUG00000023285
ENSOCUG00000016909	ENSOCUG00000023455
ENSOCUG00000017043	ENSOCUG00000023547
ENSOCUG00000017102	ENSOCUG00000023778
ENSOCUG00000017136	ENSOCUG00000024091
ENSOCUG00000017197	ENSOCUG00000024196
ENSOCUG00000017516	ENSOCUG00000024492
ENSOCUG00000017689	ENSOCUG00000025107
ENSOCUG00000021126	ENSOCUG00000025241
ENSOCUG00000021209	ENSOCUG00000025501
ENSOCUG00000021411	ENSOCUG00000025698
ENSOCUG00000021423	ENSOCUG00000025809
ENSOCUG00000022047	ENSOCUG00000025901
ENSOCUG00000022434	ENSOCUG00000025992
ENSOCUG00000022543	ENSOCUG00000026203
ENSOCUG00000022659	ENSOCUG00000026303
ENSOCUG00000023005	ENSOCUG00000026482
ENSOCUG00000023455	ENSOCUG00000026551
ENSOCUG00000023796	ENSOCUG00000027492
ENSOCUG00000024019	ENSOCUG00000027755
ENSOCUG00000024465	ENSOCUG00000027771
ENSOCUG00000024506	ENSOCUG00000027844
ENSOCUG00000024939	ENSOCUG00000029029
ENSOCUG00000025107	ENSOCUG00000029066
ENSOCUG00000025132	ENSOCUG00000029235
ENSOCUG00000025236	ENSOCUG00000029412
ENSOCUG00000025273	ENSOCUG00000029465
ENSOCUG00000025494	ENSOCUG00000029599
ENSOCUG00000025657	ENSOCUG00000029634

ENSOCUG00000025868	ENSOCUG00000029690
ENSOCUG00000026783	ENSOCUT00000006764
ENSOCUG00000027184	ENSOCUT00000012795
ENSOCUG00000027359	ENSOCUT00000025404
ENSOCUG00000029056	
ENSOCUG00000029130	
ENSOCUG00000029254	
ENSOCUG00000029301	
ENSOCUG00000029307	
ENSOCUG00000029599	
ENSOCUG00000029623	
ENSOCUT00000002051	
ENSOCUT00000022869	
ENSOCUT00000024066	
ENSOCUT00000025404	
ENSOCUT00000027408	
ENSOCUT00000030408	

Supplementary Table S7. Functional analysis of differential expressed transcripts inherited by F2 from F1 vitrified-transferred animals, compared to those conceived naturally.

Category*	Term	Count	p-value
BP	fatty acid biosynthetic process	3	0,009
BP	cellular response to DNA damage stimulus	4	0,018
BP	chorionic trophoblast cell differentiation	2	0,038
BP	cell adhesion	4	0,046
BP	cellular response to zinc ion	2	0,053
BP	DNA unwinding involved in DNA replication	2	0,053
BP	cell proliferation	4	0,059
BP	hematopoietic progenitor cell differentiation	3	0,067
BP	mRNA stabilization	2	0,067
BP	negative regulation of growth	2	0,074
CC	extracellular matrix	4	0,020
CC	perinuclear region of cytoplasm	6	0,066
CC	nuclear chromosome	2	0,073
CC	proteinaceous extracellular matrix	4	0,076
CC	extracellular exosome	21	0,078
MF	oxidoreductase activity, acting on paired donors, with oxidation of a pair of donors resulting in the reduction of molecular oxygen to two molecules of water	3	0,000
MF	zinc ion binding	12	0,045
MF	iron ion binding	4	0,076
KEGG	PPAR signaling pathway	5	0,001
KEGG	Biosynthesis of unsaturated fatty acids	4	0,001
KEGG	Cell cycle	5	0,005
KEGG	AMPK signaling pathway	4	0,028
KEGG	Metabolic pathways	13	0,030
KEGG	Fatty acid metabolism	3	0,035
KEGG	p53 signaling pathway	3	0,057
KEGG	Nitrogen metabolism	2	0,082

*Functional analysis was referred to the GO term annotation according to the biological process (BP), cellular component (CC) and molecular function (MF) classification, and the KEGG pathways in which they are involved.

Supplementary Table S8. Functional analysis of differential expressed transcripts inherited by F3 from F1 vitrified-transferred animals, compared to those conceived naturally.

Category*	Term	Count	p-value
BP	acute-phase response	2	0,087
CC	extracellular matrix	7	0,000
CC	zymogen granule membrane	4	0,000
CC	extracellular exosome	20	0,033
CC	proteinaceous extracellular matrix	4	0,052
CC	anchored component of membrane	2	0,087
MF	carbohydrate binding	4	0,008
MF	glycine binding	2	0,053
KEGG	Circadian entrainment	4	0,007
KEGG	Gap junction	3	0,059
KEGG	Salivary secretion	3	0,059
KEGG	Glutamatergic synapse	3	0,087

*Functional analysis was referred to the GO term annotation according to the biological process (BP), cellular component (CC) and molecular function (MF) classification, and the KEGG pathways in which they are involved.

Supplementary Table S9. Differentially expressed transcripts in liver tissue between vitrified- transferred progeny and that naturally-conceived in F2.

Gene accession	Gene name	Fold Change
ENSOCUG00000012831	insulin like growth factor binding protein 1(IGFBP1)	-6,59
ENSOCUG00000017905	phosphoenolpyruvate carboxykinase 1(PCK1)	-5,47
ENSOCUG00000008474	CD2 molecule(CD2)	-5,19
ENSOCUG00000017694	keratin 23(KRT23)	-4,75
ENSOCUG00000029731	HLA class II histocompatibility antigen, DRB1-4 beta chain(LOC100350168)	-4,56
ENSOCUG00000001327	histamine receptor H4(HRH4)	-4,25
ENSOCUG00000012452	adhesion G protein-coupled receptor F3(ADGRF3)	-4,00
ENSOCUG00000027242	uncharacterized LOC100341342(LOC100341342)	-3,92
ENSOCUG00000012097	Ras and Rab interactor like(RINL)	-3,90
ENSOCUG00000015664	tryptophan hydroxylase 2(TPH2)	-3,68
ENSOCUG00000008210	C-C motif chemokine receptor 9(CCR9)	-3,58
ENSOCUG00000004897	fms related tyrosine kinase 3(FLT3)	-3,57
ENSOCUG00000015667	SH3 domain containing ring finger 2(SH3RF2)	-3,54
ENSOCUG00000029056	C-type lectin domain family 17 member A(CLEC17A)	-3,53
ENSOCUG00000029546	lipase family member J(LIPJ)	-3,52
ENSOCUG00000013618	phosphorylase kinase catalytic subunit gamma 1(PHKG1)	-3,49
ENSOCUG00000012752	signal regulatory protein beta 2(SIRPB2)	-3,47
ENSOCUG00000026984	tigger transposable element derived 3(TIGD3)	-3,46
ENSOCUG00000029347	neural retina leucine zipper(NRL)	-3,43
ENSOCUG00000013315	Cbp/p300 interacting transactivator with Glu/Asp rich carboxy-terminal domain 1(CITED1)	-3,37
ENSOCUG00000025132	60S ribosomal protein L23a(LOC108177184)	-3,30
ENSOCUG00000007555	peptidoglycan recognition protein 2(PGLYRP2)	-3,29
ENSOCUG00000008285	neuronal growth regulator 1(NEGR1)	-3,24
ENSOCUG00000024754	G protein subunit alpha transducin 3(GNAT3)	-3,16
ENSOCUG00000006954	perforin 1(PRF1)	-3,12
ENSOCUG00000017113	ankyrin repeat and SOCS box containing 4(ASB4)	-3,08
ENSOCUG00000026162	inhibitor of carbonic anhydrase(LOC100345698)	-3,02
ENSOCUG00000003715	lymphocyte transmembrane adaptor 1(LAX1)	-3,01
ENSOCUG00000008378	dipeptidyl peptidase like 10(DPP10)	-2,99
ENSOCUG00000011825	zinc finger DHHC-type containing 1(ZDHHC1)	-2,99
ENSOCUG00000027044	TNF receptor superfamily member 25(TNFRSF25)	-2,98
ENSOCUG00000011428	coiled-coil domain containing 30(CCDC30)	-2,95
ENSOCUG00000017102	serpin family A member 7(SERPINA7)	-2,94
ENSOCUG00000003403	high affinity immunoglobulin gamma Fc receptor I(LOC100358696)	-2,88
ENSOCUG00000028182	carbonyl reductase [NADPH] 1(LOC100345459)	-2,85
ENSOCUG00000017136	solute carrier family 27 member 6(SLC27A6)	-2,84
ENSOCUG00000000580	cadherin 16(CDH16)	-2,83
ENSOCUG00000005855	aspartoacylase(ASPA)	-2,80
ENSOCUG00000008419	solute carrier family 25 member 30(SLC25A30)	-2,73
ENSOCUG00000029599	killer cell lectin like receptor B1(KLRB1)	-2,70
ENSOCUG00000005940	aryl hydrocarbon receptor nuclear translocator like(ARNTL)	-2,66
ENSOCUG00000026619	myosin VIIB(MYO7B)	-2,65

ENSOCUG00000021209	metallothionein-2D(LOC100343557)	-2,61
ENSOCUG00000007196	chromosome unknown open reading frame, human C1orf127(LOC108176210)	-2,54
ENSOCUG00000013552	potassium calcium-activated channel subfamily N member 3(KCNN3)	-2,54
ENSOCUG00000002000	sarcoglycan delta(SGCD)	-2,51
ENSOCUG00000024883	permeability factor 2(LOC100354804)	-2,49
ENSOCUG00000014365	amyloid P component, serum(APCS)	-2,48
ENSOCUG00000010231	cholinergic receptor nicotinic epsilon subunit(CHRNE)	-2,46
ENSOCUG00000012464	CD6 molecule(CD6)	-2,43
ENSOCUG00000003246	sulfatase 1(SULF1)	-2,36
ENSOCUG00000017821	GATA binding protein 3(GATA3)	-2,36
ENSOCUG00000016751	receptor activity modifying protein 1(RAMP1)	-2,33
ENSOCUG00000015778	solute carrier family 25 member 47(SLC25A47)	-2,32
ENSOCUG00000025273	liver carboxylesterase 2(LOC100343300)	-2,32
ENSOCUG00000015483	fatty acid binding protein 4(FABP4)	-2,29
ENSOCUG00000022630	major intrinsic protein of lens fiber(MIP)	-2,28
ENSOCUG00000004936	neuron navigator 3(NAV3)	-2,27
ENSOCUG00000025663	carnitine O-palmitoyltransferase 1, liver isoform(LOC100350311)	-2,26
ENSOCUG00000005985	clusterin(CLU)	-2,25
ENSOCUG00000006009	acyl-coenzyme A thioesterase 1(LOC100344509)	-2,20
ENSOCUG00000027980	60S ribosomal protein L23a(LOC108176709)	-2,19
ENSOCUG00000005819	tyrosine-protein kinase ZAP-70(LOC100342021)	-2,17
ENSOCUG00000025236	leukocyte protein(LOC100009166)	-2,17
ENSOCUG00000014227	SEC14 like lipid binding 3(SEC14L3)	-2,15
ENSOCUG00000022646	S100 calcium binding protein A12(S100A12)	-2,12
ENSOCUG00000029573	carbonyl reductase [NADPH] 1(LOC100344692)	-2,10
ENSOCUG00000007434	peptidoglycan recognition protein 1(PGLYRP1)	-2,06
ENSOCUG00000021476	S-acyl fatty acid synthase thioesterase, medium chain(LOC100349940)	-2,04
ENSOCUG00000000901	seizure related 6 homolog like 2(SEZ6L2)	-2,04
ENSOCUG00000025590	linker for activation of T-cells(LAT)	-2,04
ENSOCUG00000009725	acyl-CoA wax alcohol acyltransferase 1(AWAT1)	-2,02
ENSOCUG00000004492	potassium voltage-gated channel subfamily Q member 1(KCNQ1)	-2,00
ENSOCUG00000029130	cytochrome P450 2C1(CYP2C1)	-1,95
ENSOCUG00000000313	ovostatin homolog 2(LOC100348825)	-1,93
ENSOCUG00000021126	Metallothionein-2A(LOC100343299)	-1,93
ENSOCUG00000004345	CD79b molecule(CD79B)	-1,92
ENSOCUG00000008793	FK506 binding protein 1B(FKBP1B)	-1,87
ENSOCUG00000008329	ADAM metallopeptidase with thrombospondin type 1 motif 19(ADAMTS19)	-1,84
ENSOCUG00000016772	DNA damage inducible transcript 4(DDIT4)	-1,82
ENSOCUG00000024174	potassium voltage-gated channel subfamily B member 1(KCNB1)	-1,81
ENSOCUG00000024529	solute carrier family 7 member 13(LOC100348219)	-1,81
ENSOCUG00000004307	ephrin A1(EFNA1)	-1,80
ENSOCUG00000015352	energy homeostasis associated(ENHO)	-1,76
ENSOCUG00000017009	junction adhesion molecule like(JAML)	-1,75
ENSOCUG00000011634	sphingomyelin phosphodiesterase 3(SMPD3)	-1,73
ENSOCUG00000005924	solute carrier family 2 member 1(SLC2A1)	-1,71
ENSOCUG00000011201	CYP4B1-like isozyme short form(CYP4B1)	-1,68
ENSOCUG00000009566	S100 calcium binding protein A8(S100A8)	-1,68
ENSOCUG00000024108	potassium voltage-gated channel subfamily C member 3(LOC100338015)	-1,64

ENSOCUG00000014352	glycine amidinotransferase(GATM)	-1,60
ENSOCUG00000005177	flavin containing monooxygenase 2(FMO2)	1,64
ENSOCUG00000001594	solute carrier family 16 member 6(SLC16A6)	1,64
ENSOCUG00000017033	transferrin receptor(TFRC)	1,65
ENSOCUG00000003477	protein phosphatase 1 regulatory subunit 3B(PPP1R3B)	1,65
ENSOCUG00000017852	aquaporin 3 (Gill blood group)(AQP3)	1,67
ENSOCUG00000002965	zinc finger protein 652(ZNF652)	1,67
ENSOCUG00000010715	tubulin gamma complex associated protein 5(TUBGCP5)	1,68
ENSOCUG00000026333	period circadian clock 1(PER1)	1,68
ENSOCUG00000005579	integrin subunit beta 3(ITGB3)	1,68
ENSOCUG00000029430	carbonyl reductase [NADPH] 1(LOC100345202)	1,68
ENSOCUG00000008551	5'-aminolevulinate synthase 1(ALAS1)	1,68
ENSOCUG00000015053	mitogen-activated protein kinase kinase kinase MLT(LOC100351826)	1,70
ENSOCUG00000013478	HtrA serine peptidase 1(HTRA1)	1,70
ENSOCUG00000017924	membrane metalloendopeptidase(MME)	1,71
ENSOCUG00000007955	TEA domain transcription factor 1(TEAD1)	1,72
ENSOCUG00000017626	integrin subunit alpha V(ITGAV)	1,72
ENSOCUG00000014907	WNT inhibitory factor 1(WIF1)	1,73
ENSOCUG00000009279	ATP binding cassette subfamily A member 1(ABCA1)	1,74
ENSOCUG00000000957	thyroid hormone responsive(THRSP)	1,75
ENSOCUG00000011526	aminoadipate aminotransferase(AADAT)	1,76
ENSOCUG00000017745	DNA polymerase epsilon, catalytic subunit(POLE)	1,76
ENSOCUG00000015924	minichromosome maintenance complex component 2(MCM2)	1,77
ENSOCUG00000002707	MID1 interacting protein 1(MID1IP1)	1,77
ENSOCUG00000029364	inositol hexakisphosphate kinase 3(IP6K3)	1,77
ENSOCUG00000016303	sulfotransferase family, cytosolic, 1C, member 2(SULT1C2)	1,78
ENSOCUG00000013643	PDZ and LIM domain 1(PDLIM1)	1,81
ENSOCUG00000000030	solute carrier family 17 member 5(SLC17A5)	1,81
ENSOCUG00000010299	cysteine and glycine rich protein 1(CSRP1)	1,81
ENSOCUG00000005032	family with sequence similarity 169 member B(FAM169B)	1,81
ENSOCUG00000027187	proteoglycan 4(PRG4)	1,83
ENSOCUG00000003696	pecanex homolog 1 (Drosophila)(PCNX1)	1,83
ENSOCUG00000012344	KH RNA binding domain containing, signal transduction associated 2(KHDRBS2)	1,84
ENSOCUG00000007890	SCO-spondin(SSPO)	1,84
ENSOCUG00000012735	coenzyme Q10B(COQ10B)	1,86
ENSOCUG00000015939	lactate dehydrogenase A(LDHA)	1,88
ENSOCUG00000017197	claudin 6(CLDN6)	1,89
ENSOCUG00000027184	carbonyl reductase 1(CBR1)	1,90
ENSOCUG00000000483	calpain 9(CAPN9)	1,90
ENSOCUG00000004393	synaptotagmin-15(LOC100351528)	1,90
ENSOCUG00000013550	upstream transcription factor family member 3(USF3)	1,90
ENSOCUG00000011308	microtubule associated serine/threonine kinase like(MASTL)	1,90
ENSOCUG00000014944	aldolase, fructose-bisphosphate B(ALDOB)	1,90
ENSOCUG00000014983	PPFIA binding protein 2(PPFIBP2)	1,91
ENSOCUG00000007109	diaphanous related formin 2(DIAPH2)	1,93
ENSOCUG00000010708	kinesin family member C1(KIFC1)	1,93
ENSOCUG00000007887	ATP binding cassette subfamily C member 5(ABCC5)	1,93
ENSOCUG00000004543	apoptosis inducing factor, mitochondria associated 2(AIFM2)	1,94

ENSOCUG00000010916	sacsin molecular chaperone(SACS)	1,94
ENSOCUG00000005927	acyl-CoA synthetase medium-chain family member 5(ACSM5)	1,95
ENSOCUG00000027823	amine sulfotransferase-like(LOC100338493)	1,96
ENSOCUG00000006490	leucine-rich repeat-containing protein 37A2(LOC103346478)	1,96
ENSOCUG00000005415	peroxisomal membrane protein 4(PXMP4)	1,97
ENSOCUG00000024939	interferon induced protein with tetratricopeptide repeats 2(IFIT2)	1,97
ENSOCUG00000011340	cell division cycle 20(CDC20)	1,97
ENSOCUG00000007432	acetyl-CoA carboxylase beta(ACACB)	1,98
ENSOCUG00000021923	cut like homeobox 2(CUX2)	1,98
ENSOCUG00000001335	myosin IA(MYO1A)	1,98
ENSOCUG00000006357	ADAM metallopeptidase with thrombospondin type 1 motif 12(ADAMTS12)	1,99
ENSOCUG00000007844	SOS Ras/Rac guanine nucleotide exchange factor 1(SOS1)	1,99
ENSOCUG00000008478	syntaxin binding protein 4(STXBP4)	2,00
ENSOCUG00000004231	BTB domain and CNC homolog 2(BACH2)	2,01
ENSOCUG00000005912	1,4-alpha-glucan branching enzyme 1(GBE1)	2,01
ENSOCUG00000027006	UDP-glucuronosyltransferase 2B16-like(LOC100340258)	2,03
ENSOCUG00000024065	nuclear prelamin A recognition factor(NARF)	2,04
ENSOCUG00000017689	keratin 20(KRT20)	2,04
ENSOCUG00000017427	3-hydroxy-3-methylglutaryl-CoA reductase(HMGCR)	2,04
ENSOCUG00000002814	topoisomerase (DNA) II alpha(TOP2A)	2,04
ENSOCUG00000006386	ATPase phospholipid transporting 10A (putative)(ATP10A)	2,05
ENSOCUG00000007382	inhibin beta E subunit(INHBE)	2,06
ENSOCUG00000003203	TPX2, microtubule nucleation factor(TPX2)	2,06
ENSOCUG00000016909	RAD51 recombinase(RAD51)	2,07
ENSOCUG00000013212	ATP citrate lyase(ACLY)	2,08
ENSOCUG00000003071	lipin 1(LPIN1)	2,08
ENSOCUG00000008872	alanyl-tRNA synthetase(AARS)	2,09
ENSOCUG00000014077	latent transforming growth factor beta binding protein 2(LTBP2)	2,09
ENSOCUG00000022715	zinc finger homeobox 2(ZFHX2)	2,12
ENSOCUG00000022885	cytochrome c oxidase subunit 6C(LOC100352363)	2,12
ENSOCUG00000002297	uncoupling protein 1(UCP1)	2,12
ENSOCUG00000014801	acyl-CoA desaturase(LOC100346561)	2,13
ENSOCUG00000000109	ADAM metallopeptidase with thrombospondin type 1 motif 9(ADAMTS9)	2,14
ENSOCUG00000009367	SET domain containing lysine methyltransferase 7(SETD7)	2,14
ENSOCUG00000017118	adiponectin receptor 2(ADIPOR2)	2,14
ENSOCUG00000007182	growth arrest specific 2 like 3(GAS2L3)	2,15
ENSOCUG00000005820	ribonucleotide reductase regulatory subunit M2(RRM2)	2,17
ENSOCUG00000006877	transcription factor CP2 like 1(TFCP2L1)	2,21
ENSOCUG00000001375	acyl-CoA desaturase(LOC100346046)	2,21
ENSOCUG00000013772	tubulin delta 1(TUBD1)	2,23
ENSOCUG00000013111	procollagen C-endopeptidase enhancer 2(PCOLCE2)	2,26
ENSOCUG00000007516	6-phosphofructo-2-kinase/fructose-2,6-biphosphatase 3(PFKFB3)	2,29
ENSOCUG00000013474	calcium voltage-gated channel subunit alpha1 D(CACNA1D)	2,29
ENSOCUG00000012071	glycerol-3-phosphate acyltransferase 3(GPAT3)	2,30
ENSOCUG00000012148	inositol 1,4,5-trisphosphate receptor type 1(ITPR1)	2,30
ENSOCUG00000016347	phosphoethanolamine/phosphocholine phosphatase(PHOSPHO1)	2,31
ENSOCUG00000007172	acyl-CoA desaturase(LOC100357419)	2,32
ENSOCUG00000001171	NUF2, NDC80 kinetochore complex component(NUF2)	2,32

ENSOCUG00000007690	zinc finger CCCH-type containing 12D(ZC3H12D)	2,32
ENSOCUG00000012902	glutathione S-transferase Yc(LOC100353428)	2,33
ENSOCUG00000013740	phosphoglucomutase 2(PGM2)	2,34
ENSOCUG00000024465	collagen type VIII alpha 1 chain(COL8A1)	2,35
ENSOCUG00000011953	centromere protein I(CENPI)	2,35
ENSOCUG00000026935	GULP, engulfment adaptor PTB domain containing 1(GULP1)	2,35
ENSOCUG00000007701	KN motif and ankyrin repeat domains 4(KANK4)	2,36
ENSOCUG00000013414	RasGEF domain family member 1C(RASGEF1C)	2,37
ENSOCUG00000005208	methylenetetrahydrofolate dehydrogenase (NADP+ dependent) 2, methenyltetrahydrofolate cyclohydrolase(MTHFD2)	2,37
ENSOCUG00000015780	transcobalamin 1(TCN1)	2,38
ENSOCUG00000003467	prostate stem cell antigen(PSCA)	2,38
ENSOCUG00000017133	glycerophosphodiester phosphodiesterase domain containing 2(GDPD2)	2,40
ENSOCUG00000013807	sperm associated antigen 5(SPAG5)	2,42
ENSOCUG00000008316	striatin interacting protein 2(STRIP2)	2,42
ENSOCUG00000008179	rhomboid like 3(RHBDL3)	2,42
ENSOCUG00000007893	glycerol-3-phosphate acyltransferase, mitochondrial(GPAM)	2,43
ENSOCUG00000005956	aldehyde dehydrogenase 18 family member A1(ALDH18A1)	2,44
ENSOCUG00000015681	Rac GTPase activating protein 1(RACGAP1)	2,44
ENSOCUG00000027359	tetraspanin 11(TSPAN11)	2,45
ENSOCUG00000012480	bestrophin 4(BEST4)	2,50
ENSOCUG00000005900	fibulin 2(FBLN2)	2,54
ENSOCUG00000010012	adenylate kinase 4(AK4)	2,57
ENSOCUG00000003768	leukemia inhibitory factor(LIF)	2,59
ENSOCUG00000011000	growth arrest specific 7(GAS7)	2,61
ENSOCUG00000022434	cell division cycle associated 3(CDCA3)	2,62
ENSOCUG00000006777	family with sequence similarity 102 member B(FAM102B)	2,62
ENSOCUG00000014453	acetyl-CoA carboxylase alpha(ACACA)	2,68
ENSOCUG00000009379	MIS18 kinetochore protein A(MIS18A)	2,70
ENSOCUG00000011957	aurora kinase B(AURKB)	2,71
ENSOCUG00000013069	ELOVL fatty acid elongase 6(ELOVL6)	2,72
ENSOCUG00000014969	pyruvate kinase, liver and RBC(PKLR)	2,72
ENSOCUG00000008841	glutamate-ammonia ligase(GLUL)	2,72
ENSOCUG00000004106	glycine decarboxylase(GLDC)	2,74
ENSOCUG00000010776	Fanconi anemia complementation group B(FANCB)	2,77
ENSOCUG00000000215	period circadian clock 2(PER2)	2,77
ENSOCUG00000022945	asparagine synthetase (glutamine-hydrolyzing)(ASNS)	2,80
ENSOCUG00000022659	EF-hand and coiled-coil domain containing 1(EFCC1)	2,80
ENSOCUG00000013819	ribosomal protein S6 kinase like 1(RPS6KL1)	2,87
ENSOCUG00000023520	fibronectin leucine rich transmembrane protein 1(FLRT1)	2,91
ENSOCUG00000029751	transmembrane 9 superfamily member 2(LOC100353059)	2,92
ENSOCUG00000002703	forkhead box M1(FOXM1)	2,96
ENSOCUG00000007124	TTK protein kinase(TTK)	3,03
ENSOCUG00000029228	leucine rich repeat containing 19(LRRC19)	3,04
ENSOCUG00000009736	neurotrophic receptor tyrosine kinase 2(NTRK2)	3,04
ENSOCUG00000007213	high mobility group box 4(HMGB4)	3,05
ENSOCUG00000006288	ninein like(NINL)	3,07
ENSOCUG00000004232	zinc finger and BTB domain containing 20(ZBTB20)	3,08

ENSOCUG00000003048	tubulin polymerization promoting protein(TPPP)	3,09
ENSOCUG00000013321	retinoic acid early transcript 1E(RAET1E)	3,14
ENSOCUG00000006801	minichromosome maintenance 10 replication initiation factor(MCM10)	3,16
ENSOCUG00000000856	E2F transcription factor 1(E2F1)	3,23
ENSOCUG00000011488	Fanconi anemia complementation group I(FANCI)	3,23
ENSOCUG00000003876	FH2 domain containing 1(FHDC1)	3,23
ENSOCUG00000007759	ubiquitin specific peptidase 2(USP2)	3,23
ENSOCUG00000002858	cyclin dependent kinase 1(CDK1)	3,24
ENSOCUG00000015803	tubulointerstitial nephritis antigen(TINAG)	3,30
ENSOCUG00000016718	mesenteric estrogen dependent adipogenesis(MEDAG)	3,32
ENSOCUG00000013508	interleukin 17 receptor B(IL17RB)	3,32
ENSOCUG00000029623	ATPase plasma membrane Ca2+ transporting 3(ATP2B3)	3,37
ENSOCUG00000026022	derlin 3(DERL3)	3,40
ENSOCUG00000022047	MPV17 mitochondrial inner membrane protein like(MPV17L)	3,40
ENSOCUG00000025868	gamma-aminobutyric acid type A receptor delta subunit(GABRD)	3,41
ENSOCUG00000005465	nocturnin(NOCT)	3,45
ENSOCUG00000029254	family with sequence similarity 159 member A(FAM159A)	3,45
ENSOCUG00000011025	contactin 4(CNTN4)	3,48
ENSOCUG00000016815	islet cell autoantigen 1(ICA1)	3,53
ENSOCUG00000004932	E2F transcription factor 7(E2F7)	3,61
ENSOCUG00000001673	triokinase and FMN cyclase(TKFC)	3,68
ENSOCUG00000009260	phospholipase C eta 1(PLCH1)	3,73
ENSOCUG00000001652	cyclin B2(CCNB2)	3,74
ENSOCUG00000011124	fibroblast growth factor 18(FGF18)	3,78
ENSOCUG00000009071	kinesin family member 24(KIF24)	3,79
ENSOCUG00000011656	actin like 7A(ACTL7A)	3,94
ENSOCUG00000001129	solute carrier family 26 member 3(SLC26A3)	3,98
ENSOCUG00000004485	E2F transcription factor 8(E2F8)	4,17
ENSOCUG00000003649	transmembrane and immunoglobulin domain containing 1(TMIGD1)	4,21
ENSOCUG00000006962	aldehyde dehydrogenase 1 family member L2(ALDH1L2)	4,25
ENSOCUG00000021411	tsukushi, small leucine rich proteoglycan(TSKU)	4,28
ENSOCUG00000007427	FAT atypical cadherin 3(FAT3)	4,39
ENSOCUG00000013792	kinetochore associated 1(KNTC1)	4,40
ENSOCUG00000001627	solute carrier family 6 member 11(SLC6A11)	4,45
ENSOCUG00000000144	calcium voltage-gated channel auxiliary subunit beta 4(CACNB4)	4,50
ENSOCUG00000025494	secretogranin III(SCG3)	4,81
ENSOCUG00000009557	polypeptide N-acetylgalactosaminyltransferase 8(GALNT8)	4,82
ENSOCUG00000009880	dual specificity phosphatase 14(DUSP14)	4,95
ENSOCUG00000027371	kinesin family member 2C(KIF2C)	4,98
ENSOCUG00000010814	malic enzyme 1(ME1)	5,02
ENSOCUG00000017054	testis expressed 33(TEX33)	5,19
ENSOCUG00000017043	cell division cycle associated 8(CDCA8)	5,19
ENSOCUG00000013544	ATPase H+ transporting V0 subunit a4(ATP6V0A4)	6,00
ENSOCUG00000001376	sodium channel protein type 11 subunit alpha(LOC100349709)	7,15
ENSOCUG00000006498	desmoglein 1(DSG1)	< -0,01
ENSOCUG00000015925	TNF receptor superfamily member 8(TNFRSF8)	< -0,02
ENSOCUG00000016573	carboxypeptidase, vitellogenic like(CPVL)	< -0,02
ENSOCUG00000029301	zinc finger and SCAN domain containing 23(ZSCAN23)	< -0,02

ENSOCUG00000004632	carbonic anhydrase 12(CA12)	< -0,03
ENSOCUG00000005540	calpain 6(CAPN6)	< -0,03
ENSOCUG00000010540	cyclic nucleotide gated channel beta 1(CNGB1)	< -0,03
ENSOCUG00000012845	SERTA domain containing 4(SERTAD4)	< -0,03
ENSOCUG00000016508	solute carrier family 12 member 1(SLC12A1)	< -0,03
ENSOCUG00000023218	left-right determination factor 2(LOC100101568)	< -0,03
ENSOCUG00000012514	X-ray radiation resistance associated 1(XRRA1)	< -0,04
ENSOCUG00000001190	GDNF family receptor alpha 2(GFRA2)	< -0,05
ENSOCUG00000002522	prostaglandin F receptor(PTGFR)	< -0,05
ENSOCUG00000003021	ceramide kinase like(CERKL)	< -0,05
ENSOCUG00000026570	zinc finger protein 296(ZNF296)	< -0,06
ENSOCUG00000014051	interleukin 17 receptor D(IL17RD)	< -0,07
ENSOCUG00000000771	C-C motif chemokine 3-like 1(LOC100348776)	< -0,08
ENSOCUG00000023796	lactotransferrin(LTF)	< -0,08
ENSOCUG00000013086	cytotoxic and regulatory T-cell molecule(CRTAM)	< -0,09
ENSOCUG00000004825	dopamine receptor D1(DRD1)	< -0,10
ENSOCUG00000023342	reticulon 2(RTN2)	< -0,10
ENSOCUG00000005968	purinergic receptor P2Y8(P2RY8)	< -0,11
ENSOCUG00000022151	doublecortin domain containing 2B(DCDC2B)	< -0,11
ENSOCUG00000006864	T-cell surface glycoprotein CD3 epsilon chain(LOC100339529)	< -0,12
ENSOCUG00000015699	acid sensing ion channel subunit 1(ASIC1)	< -0,13
ENSOCUG00000014788	tudor domain containing 9(TDRD9)	< -0,17
ENSOCUG00000009994	transmembrane 6 superfamily member 1(TM6SF1)	< -0,19
ENSOCUG00000015805	NADPH oxidase 5(NOX5)	< -0,19
ENSOCUG00000025107	membrane associated ring-CH-type finger 4(MARCH4)	< -0,19
ENSOCUG00000021259	coiled-coil domain containing 74B(CCDC74B)	< -0,20
ENSOCUG00000001717	src kinase associated phosphoprotein 1(SKAP1)	< -0,21
ENSOCUG00000027509	C-X3-C motif chemokine receptor 1(CX3CR1)	< -0,21
ENSOCUG00000001863	vesicle amine transport 1 like(VAT1L)	< -0,22
ENSOCUG00000002937	ankyrin repeat and death domain containing 1B(ANKDD1B)	< -0,25
ENSOCUG00000027321	proline-serine-threonine phosphatase interacting protein 1(PSTPIP1)	< -0,31
ENSOCUG00000022335	defensin NP-3a(LOC100009134)	< -0,60
ENSOCUG00000016243	metallothionein 3(MT3)	< -0,97
ENSOCUG00000004688	polycystic kidney and hepatic disease 1 (autosomal recessive)-like 1(PKHD1L1)	> 0,00
ENSOCUG00000011128	GLI family zinc finger 1(GLI1)	> 0,02
ENSOCUG00000000953	RecQ mediated genome instability 2(RMI2)	> 0,04
ENSOCUG00000005159	anillin actin binding protein(ANLN)	> 0,04
ENSOCUG00000006290	leucine-rich repeat and fibronectin type-III domain-containing protein 5(LOC100349270)	> 0,04
ENSOCUG00000001617	myotubularin related protein 8(MTMR8)	> 0,05
ENSOCUG00000004633	prostaglandin E receptor 3(PTGER3)	> 0,07
ENSOCUG00000011298	family with sequence similarity 83 member D(FAM83D)	> 0,07
ENSOCUG00000004978	cartilage acidic protein 1(CRTAC1)	> 0,08
ENSOCUG00000022194	family with sequence similarity 72 member A(FAM72A)	> 0,08
ENSOCUG00000029469	phosphatidylinositol 3,4,5-trisphosphate 3-phosphatase TPTE2(LOC100346342)	> 0,08
ENSOCUG00000015149	thrombospondin type laminin G domain and EAR repeats(TSPEAR)	> 0,09
ENSOCUG00000015368	family with sequence similarity 83 member A(FAM83A)	> 0,09
ENSOCUG00000016102	allantoicase(ALLC)	> 0,09

ENSOCUG00000001278	inturned planar cell polarity protein(INTU)	> 0,11
ENSOCUG00000015283	chromosome 9 open reading frame, human C18orf54(C9H18orf54)	> 0,11
ENSOCUG00000006484	kinesin family member 27(KIF27)	> 0,12
ENSOCUG00000002926	DLG associated protein 5(DLGAP5)	> 0,13
ENSOCUG00000000197	hexokinase 2(HK2)	> 0,15
ENSOCUG00000010181	Rho guanine nucleotide exchange factor 39(ARHGEF39)	> 0,16
ENSOCUG00000010249	ubiquitin conjugating enzyme E2 T(UBE2T)	> 0,17
ENSOCUG00000029613	zymogen granule membrane protein 16-like(LOC100358477)	> 0,22
ENSOCUG00000002878	SLX4 interacting protein(SLX4IP)	> 0,23
ENSOCUG00000009066	sterile alpha motif domain containing 12(SAMD12)	> 0,25
ENSOCUG00000009652	family with sequence similarity 229 member B(FAM229B)	> 0,28
ENSOCUG00000023005	zinc finger protein 367(ZNF367)	> 0,46
ENSOCUG00000010723	solute carrier family 51 beta subunit(SLC51B)	> 0,53
ENSOCUG00000010762	ChaC glutathione specific gamma-glutamylcyclotransferase 1(CHAC1)	> 0,58

Supplementary Table S10. Functional analysis of the differentially expressed transcripts in liver tissue between vitrified- transferred progeny and that naturally-conceived in F2.

Category*	Term	Count	p-value
BP	fatty acid biosynthetic process	5	0,00099
BP	chorionic trophoblast cell differentiation	3	0,0056
BP	biosynthetic process	4	0,01
BP	phagocytosis, engulfment	3	0,015
BP	triglyceride biosynthetic process	3	0,015
BP	trophoblast giant cell differentiation	3	0,023
BP	positive regulation of inflammatory response	4	0,027
BP	positive regulation of osteoblast proliferation	3	0,028
BP	positive regulation of tumor necrosis factor production	4	0,03
BP	astrocyte development	3	0,039
BP	positive regulation of DNA endoreduplication	2	0,048
BP	negative regulation of natural killer cell differentiation involved in immune response	2	0,048
BP	growth of symbiont in host	2	0,048
BP	immune response	8	0,05
BP	negative regulation of interferon-gamma production	3	0,057
BP	circadian regulation of gene expression	4	0,064
BP	acetyl-CoA metabolic process	2	0,071
BP	negative regulation of transcription involved in G1/S transition of mitotic cell cycle	2	0,071
BP	aortic valve morphogenesis	2	0,071
BP	regulation of glucose import	2	0,071
BP	positive regulation of interleukin-5 secretion	2	0,094
BP	regulation of hair cycle	2	0,094
BP	hepatocyte differentiation	2	0,094
BP	peptidoglycan catabolic process	2	0,094
BP	mitotic spindle midzone assembly	2	0,094
BP	detection of bacterium	2	0,094
BP	signaling	2	0,094
BP	positive regulation of interleukin-13 secretion	2	0,094
BP	cellular response to DNA damage stimulus	5	0,098
CC	cell-cell junction	7	0,018
CC	T cell receptor complex	3	0,019
CC	extracellular matrix	6	0,026
CC	extracellular exosome	54	0,043
CC	chromosome passenger complex	2	0,065
CC	external side of plasma membrane	7	0,093
CC	mitochondrial membrane	3	0,098
MF	oxidoreductase activity, acting on paired donors, with oxidation of a pair of donors resulting in the reduction of molecular oxygen to two molecules of water	3	0,0014
MF	drug binding	4	0,029
MF	ATP binding	33	0,041
MF	N-acetylmuramoyl-L-alanine amidase activity	2	0,043
MF	acetyl-CoA carboxylase activity	2	0,043

MF	peptidoglycan receptor activity	2	0,043
MF	hydrolase activity, acting on ester bonds	3	0,046
MF	calcium ion binding	16	0,066
MF	E-box binding	3	0,082
MF	glycerol-3-phosphate O-acyltransferase activity	2	0,084
MF	biotin carboxylase activity	2	0,084
MF	microtubule motor activity	4	0,095
KEGG	Metabolic pathways	45	0,00016
KEGG	PPAR signaling pathway	8	0,00058
KEGG	Pyruvate metabolism	6	0,0022
KEGG	Glucagon signaling pathway	8	0,0033
KEGG	AMPK signaling pathway	9	0,0035
KEGG	Biosynthesis of unsaturated fatty acids	5	0,0037
KEGG	Fatty acid metabolism	6	0,0052
KEGG	Glycolysis / Gluconeogenesis	6	0,012
KEGG	Biosynthesis of antibiotics	11	0,018
KEGG	Insulin signaling pathway	8	0,018
KEGG	Hematopoietic cell lineage	6	0,02
KEGG	Fanconi anemia pathway	5	0,02
KEGG	Fatty acid biosynthesis	3	0,023
KEGG	Metabolism of xenobiotics by cytochrome P450	6	0,036
KEGG	Adipocytokine signaling pathway	5	0,046
KEGG	Arrhythmogenic right ventricular cardiomyopathy (ARVC)	5	0,05
KEGG	Mineral absorption	4	0,052
KEGG	Insulin resistance	6	0,063
KEGG	Chemical carcinogenesis	6	0,063
KEGG	Carbon metabolism	6	0,1

*Functional analysis was referred to the GO term annotation according to the biological process (BP), cellular component (CC) and molecular function (MF) classification, and the KEGG pathways in which they are involved.

Supplementary Table S11. Differentially expressed transcripts in liver tissue between vitrified- transferred progeny and that naturally-conceived in F3.

Gene accession	Gene name	Fold change
ENSOCUG00000014290	dual oxidase maturation factor 2(LOC100355642)	-5,06
ENSOCUG00000001277	N-terminal EF-hand calcium binding protein 1(NECAB1)	-4,70
ENSOCUG00000009306	La ribonucleoprotein domain family member 6(LARP6)	-4,48
ENSOCUG00000007726	ST6 beta-galactoside alpha-2,6-sialyltransferase 2(ST6GAL2)	-4,29
ENSOCUG00000000144	calcium voltage-gated channel auxiliary subunit beta 4(CACNB4)	-4,26
ENSOCUG00000000658	bone morphogenetic protein 3(BMP3)	-4,23
ENSOCUG00000009399	carbonic anhydrase 4(CA4)	-4,22
ENSOCUG00000009494	casein beta(CSN2)	-4,19
ENSOCUG00000017347	leptin receptor(LEPR)	-4,08
ENSOCUG00000000706	formin homology 2 domain containing 3(FHOD3)	-4,07
ENSOCUG00000015552	plexin A4(PLXNA4)	-4,06
ENSOCUG00000015764	cyclin dependent kinase like 3(CDKL3)	-3,97
ENSOCUG00000013140	calmodulin binding transcription activator 1(CAMTA1)	-3,93
ENSOCUG00000000370	ADAM metallopeptidase with thrombospondin type 1 motif 8(ADAMTS8)	-3,87
ENSOCUG00000001033	opioid receptor delta 1(OPRD1)	-3,86
ENSOCUG00000012661	protein tyrosine phosphatase, receptor type U(PTPRU)	-3,83
ENSOCUG00000016712	delta/notch like EGF repeat containing(DNER)	-3,79
ENSOCUG00000012685	paired like homeodomain 2(PITX2)	-3,77
ENSOCUG00000006833	dynein axonemal heavy chain 12(DNAH12)	-3,76
ENSOCUG00000023975	butyrophilin subfamily 1 member A1-like(LOC100351205)	-3,71
ENSOCUG00000000948	gamma-aminobutyric acid type A receptor rho1 subunit(GABRR1)	-3,70
ENSOCUG00000017171	fibroblast growth factor 21(FGF21)	-3,65
ENSOCUG00000008297	proteoglycan 2, pro eosinophil major basic protein(PRG2)	-3,59
ENSOCUG00000014727	actin like 6B(ACTL6B)	-3,56
ENSOCUG00000024982	butyrophilin-like protein 1(LOC100009510)	-3,53
ENSOCUG00000009285	chromosome 1 open reading frame, human C11orf53(C1H11orf53)	-3,52
ENSOCUG00000015664	tryptophan hydroxylase 2(TPH2)	-3,51
ENSOCUG00000027714	solute carrier family 23 member 1(LOC100342535)	-3,50
ENSOCUG00000014660	connector enhancer of kinase suppressor of Ras 2(CNKSR2)	-3,49
ENSOCUG00000023263	NDUFA4, mitochondrial complex associated like 2(NDUFA4L2)	-3,42
ENSOCUG00000004632	carbonic anhydrase 12(CA12)	-3,40
ENSOCUG00000005134	kinocilin(KNCN)	-3,34
ENSOCUG00000008047	von Willebrand factor A domain containing 3A(VWA3A)	-3,34
ENSOCUG00000010733	cystic fibrosis transmembrane conductance regulator(CFTR)	-3,28
ENSOCUG00000000612	CD101 molecule(CD101)	-3,22
ENSOCUG00000014767	phosphoprotein membrane anchor with glycosphingolipid microdomains 1(PAG1)	-3,21
ENSOCUG00000009532	AF4/FMR2 family member 3(AFF3)	-3,20
ENSOCUG00000001788	RAS guanyl releasing protein 1(RASGRP1)	-3,18
ENSOCUG00000003735	hydroxycarboxylic acid receptor 1(HCAR1)	-3,16
ENSOCUG00000006107	GRAM domain containing 2(GRAMD2)	-3,16
ENSOCUG00000010199	oncostatin M(OSM)	-3,15
ENSOCUG00000008771	interleukin 1 alpha(IL1A)	-3,13

ENSOCUG00000007124	TTK protein kinase(TTK)	-3,11
ENSOCUG00000003013	ADP ribosylation factor like GTPase 11(ARL11)	-3,11
ENSOCUG00000017852	aquaporin 3 (Gill blood group)(AQP3)	-3,06
ENSOCUG00000026551	lysophosphatidic acid receptor 5(LPAR5)	-3,05
ENSOCUG00000029634	arachidonate lipoxygenase 3(ALOXE3)	-3,01
ENSOCUG00000000092	serpin family F member 1(SERPINF1)	-2,97
ENSOCUG00000015893	proprotein convertase subtilisin/kexin type 2(PCSK2)	-2,95
ENSOCUG00000025837	NFAT activating protein with ITAM motif 1(NFAM1)	-2,93
ENSOCUG00000015699	acid sensing ion channel subunit 1(ASIC1)	-2,91
ENSOCUG00000011279	membrane associated ring-CH-type finger 1(MARCH1)	-2,90
ENSOCUG00000008082	GTPase activating Rap/RanGAP domain like 3(GARNL3)	-2,90
ENSOCUG00000025842	chromosome 19 open reading frame, human C17orf97(C19H17orf97)	-2,88
ENSOCUG00000012963	suppressor of cancer cell invasion(SCAI)	-2,87
ENSOCUG00000000877	mast cell expressed membrane protein 1(MCEMP1)	-2,85
ENSOCUG00000005959	arachidonate 12-lipoxygenase, 12R type(ALOX12B)	-2,85
ENSOCUG00000016029	IQ motif containing D(IQCD)	-2,83
ENSOCUG00000025779	C-C motif chemokine receptor 6(CCR6)	-2,82
ENSOCUG00000024244	C-C motif chemokine receptor 4(CCR4)	-2,80
ENSOCUG00000001355	IKAROS family zinc finger 3(IKZF3)	-2,78
ENSOCUG00000001103	caspase 1(CASP1)	-2,77
ENSOCUG00000008303	matrix metallopeptidase 12(MMP12)	-2,77
ENSOCUG00000021508	chromosome 16 open reading frame, human C1orf106(C16H1orf106)	-2,75
ENSOCUG00000026482	UDP-glucuronosyltransferase 2B16-like(LOC100340513)	-2,75
ENSOCUG00000007438	complement C3a receptor 1(C3AR1)	-2,74
ENSOCUG00000013226	BLK proto-oncogene, Src family tyrosine kinase(BLK)	-2,74
ENSOCUG00000015787	beaded filament structural protein 1(BFSP1)	-2,73
ENSOCUG00000006204	ubiquitin associated and SH3 domain containing A(UBASH3A)	-2,73
ENSOCUG00000005159	anillin actin binding protein(ANLN)	-2,72
ENSOCUG00000010247	ATPase H+ transporting V0 subunit d2(ATP6V0D2)	-2,72
ENSOCUG00000014255	dual oxidase 2(DUOX2)	-2,71
ENSOCUG00000003578	anterior gradient 2, protein disulphide isomerase family member(AGR2)	-2,65
ENSOCUG00000028024	immunity related GTPase M(IRGM)	-2,65
ENSOCUG00000013081	angiopoietin like 7(ANGPTL7)	-2,65
ENSOCUG00000016948	solute carrier organic anion transporter family member 4C1(SLCO4C1)	-2,64
ENSOCUG00000007151	cilia and flagella associated protein 70(CFAP70)	-2,63
ENSOCUG00000027925	leukocyte immunoglobulin-like receptor subfamily A member 2(LOC100347041)	-2,63
ENSOCUG00000025839	retinol dehydrogenase 5(RDH5)	-2,61
ENSOCUG00000008131	tripartite motif containing 62(TRIM62)	-2,59
ENSOCUG00000004961	CUGBP, Elav-like family member 2(CELF2)	-2,57
ENSOCUG00000002622	keratin 75(KRT75)	-2,56
ENSOCUG00000027244	uncharacterized LOC100347962(LOC100347962)	-2,56
ENSOCUG00000003970	transgelin 3(TAGLN3)	-2,55
ENSOCUG00000002486	collagen type VI alpha 6 chain(COL6A6)	-2,55
ENSOCUG00000016613	ring finger protein 222(RNF222)	-2,54
ENSOCUG00000009113	phosphodiesterase 1C(PDE1C)	-2,53
ENSOCUG00000004874	BTB/POZ domain-containing protein KCTD12(LOC100347604)	-2,50
ENSOCUG00000013273	membrane spanning 4-domains A15(MS4A15)	-2,50
ENSOCUG00000005107	HOP homeobox(HOPX)	-2,48

ENSOCUG00000003156	chromosome 1 open reading frame, human C11orf16(C1H11orf16)	-2,47
ENSOCUG00000006958	transmembrane protein 173(TMEM173)	-2,46
ENSOCUG00000009528	intestinal-type alkaline phosphatase-like(LOC100352107)	-2,46
ENSOCUG00000022422	colorectal cancer associated 2(COLCA2)	-2,45
ENSOCUG00000022151	doublecortin domain containing 2B(DCDC2B)	-2,44
ENSOCUG00000021559	alkaline ceramidase 3(ACER3)	-2,42
ENSOCUG00000007566	actin, alpha 1, skeletal muscle(ACTA1)	-2,41
ENSOCUG00000016789	DENN domain containing 2C(DENND2C)	-2,41
ENSOCUG00000016508	solute carrier family 12 member 1(SLC12A1)	-2,40
ENSOCUG00000024433	Fc fragment of IgE receptor II(FCER2)	-2,40
ENSOCUG00000009077	membrane spanning 4-domains A2(MS4A2)	-2,38
ENSOCUG00000000962	origin recognition complex subunit 1(ORC1)	-2,38
ENSOCUG00000003665	C-C motif chemokine receptor 5(CCR5)	-2,36
ENSOCUG00000017547	calcium voltage-gated channel subunit alpha1 E(CACNA1E)	-2,36
ENSOCUG00000017805	caprin family member 2(CAPRIN2)	-2,36
ENSOCUG00000025413	cytohesin 4(CYTH4)	-2,35
ENSOCUG00000008543	tenascin N(TNN)	-2,34
ENSOCUG00000007053	steroid 21-hydroxylase(LOC100342636)	-2,33
ENSOCUG00000027044	TNF receptor superfamily member 25(TNFRSF25)	-2,33
ENSOCUG00000021441	natural killer cell granule protein 7(NKG7)	-2,31
ENSOCUG00000010618	ankyrin 2(ANK2)	-2,31
ENSOCUG00000015057	macrophage expressed 1(MPEG1)	-2,31
ENSOCUG00000010753	G protein-coupled receptor 84(GPR84)	-2,30
ENSOCUG00000011023	nebulin related anchoring protein(NRAP)	-2,30
ENSOCUG00000023473	guanylate-binding protein 5(LOC100346209)	-2,30
ENSOCUG00000010544	carnitine O-octanoyltransferase(CROT)	-2,29
ENSOCUG00000008553	prune homolog 2(PRUNE2)	-2,28
ENSOCUG00000008666	Ras related GTP binding B(RRAGB)	-2,28
ENSOCUG00000000804	secernin 1(SCRN1)	-2,27
ENSOCUG00000001824	toll like receptor 1(TLR1)	-2,27
ENSOCUG00000017422	integrin subunit alpha X(ITGAX)	-2,27
ENSOCUG00000013948	Rho GTPase activating protein 20(ARHGAP20)	-2,24
ENSOCUG00000009313	chromosome 4 open reading frame, human C20orf196(C4H20orf196)	-2,24
ENSOCUG00000002805	adenosine monophosphate deaminase 3(AMPD3)	-2,24
ENSOCUG00000009395	contactin 1(CNTN1)	-2,24
ENSOCUG00000013757	solute carrier family 13 member 2(SLC13A2)	-2,23
ENSOCUG00000029253	guanylate-binding protein 4(LOC100346726)	-2,22
ENSOCUG00000002966	transcriptional repressor GATA binding 1(TRPS1)	-2,22
ENSOCUG00000015673	zona pellucida glycoprotein 1(ZP1)	-2,22
ENSOCUG00000010984	HLA class I histocompatibility antigen, A-36 alpha chain(LOC100355380)	-2,20
ENSOCUG00000022093	aggrecan(ACAN)	-2,20
ENSOCUG00000003215	sprouty RTK signaling antagonist 4(SPRY4)	-2,20
ENSOCUG00000007196	chromosome unknown open reading frame, human C1orf127(LOC108176210)	-2,20
ENSOCUG00000002956	copine 4(CPNE4)	-2,18
ENSOCUG00000008294	uroplakin 1B(UPK1B)	-2,18
ENSOCUG00000001617	myotubularin related protein 8(MTMR8)	-2,17
ENSOCUG00000017568	pyrimidinergic receptor P2Y6(P2RY6)	-2,16
ENSOCUG00000022761	interleukin 3 receptor subunit alpha(IL3RA)	-2,16

ENSOCUG00000007669	myosin VA(MYO5A)	-2,15
ENSOCUG00000023342	reticulon 2(RTN2)	-2,14
ENSOCUG00000014857	collagen type V alpha 3 chain(COL5A3)	-2,13
ENSOCUG00000029066	transient receptor potential cation channel subfamily M member 2(LOC103347481)	-2,13
ENSOCUG00000004317	C-X-C motif chemokine receptor 6(CXCR6)	-2,13
ENSOCUG00000015956	lymphoid restricted membrane protein(LRMP)	-2,12
ENSOCUG00000012244	perilipin 2(PLIN2)	-2,12
ENSOCUG00000006003	endoplasmic reticulum protein 27(ERP27)	-2,11
ENSOCUG00000017559	KIAA1147 ortholog(KIAA1147)	-2,11
ENSOCUG00000021037	interferon-induced GTP-binding protein Mx2(LOC100009451)	-2,10
ENSOCUG00000011452	coagulation factor XIII A chain(LOC103352261)	-2,09
ENSOCUG00000025033	KIAA0895 like(KIAA0895L)	-2,08
ENSOCUG00000012014	solute carrier family 15 member 1(SLC15A1)	-2,08
ENSOCUG00000004314	transient receptor potential cation channel subfamily M member 1(TRPM1)	-2,07
ENSOCUG00000014109	cell division cycle associated 2(CDCA2)	-2,07
ENSOCUG00000027844	solute carrier family 24 member 1(SLC24A1)	-2,07
ENSOCUG00000008677	neuritin 1(NRN1)	-2,05
ENSOCUG00000005845	amyloid beta precursor protein binding family B member 1 interacting protein(APBB1IP)	-2,02
ENSOCUG00000007153	copine 6(CPNE6)	-2,00
ENSOCUG00000008086	leucine rich repeat containing 15(LRRC15)	-1,99
ENSOCUG00000006002	amyloid beta precursor like protein 1(APLP1)	-1,98
ENSOCUG00000014189	annexin A8(ANXA8)	-1,96
ENSOCUG00000009317	minichromosome maintenance 8 homologous recombination repair factor(MCM8)	-1,95
ENSOCUG00000012337	collagen type XXI alpha 1 chain(COL21A1)	-1,95
ENSOCUG00000004750	grainyhead like transcription factor 2(GRHL2)	-1,95
ENSOCUG00000027509	C-X3-C motif chemokine receptor 1(CX3CR1)	-1,94
ENSOCUG00000006243	WDFY family member 4(WDFY4)	-1,94
ENSOCUG00000006248	interleukin 15 receptor subunit alpha(IL15RA)	-1,94
ENSOCUG00000001241	major facilitator superfamily domain containing 4A(MFSD4A)	-1,94
ENSOCUG00000005775	MYB proto-oncogene like 2(MYBL2)	-1,94
ENSOCUG00000012157	radical S-adenosyl methionine domain containing 2(RSAD2)	-1,93
ENSOCUG00000012433	malic enzyme 2(ME2)	-1,93
ENSOCUG00000002661	protein FAM26F(LOC100353536)	-1,93
ENSOCUG00000000624	solute carrier family 26 member 6(SLC26A6)	-1,93
ENSOCUG00000000001	alcohol dehydrogenase 6(LOC100343992)	-1,92
ENSOCUG00000000580	cadherin 16(CDH16)	-1,91
ENSOCUG00000026640	sestrin 3(SESN3)	-1,91
ENSOCUG00000015222	ankyrin repeat domain 45(ANKRD45)	-1,91
ENSOCUG00000005830	HIG1 hypoxia inducible domain family member 1A(HIGD1A)	-1,91
ENSOCUG00000025013	solute carrier family 45 member 3(SLC45A3)	-1,90
ENSOCUG00000023957	mucin 1, cell surface associated(MUC1)	-1,90
ENSOCUG00000022280	transmembrane protein 45B(TMEM45B)	-1,89
ENSOCUG00000017198	tubby bipartite transcription factor(TUB)	-1,89
ENSOCUG00000013618	phosphorylase kinase catalytic subunit gamma 1(PHKG1)	-1,88
ENSOCUG00000016143	polypeptide N-acetylgalactosaminyltransferase 6(GALNT6)	-1,88
ENSOCUG00000014077	latent transforming growth factor beta binding protein 2(LTBP2)	-1,88
ENSOCUG00000008175	phosphodiesterase 7A(PDE7A)	-1,88

ENSOCUG00000009360	macrophage stimulating 1 receptor(MST1R)	-1,87
ENSOCUG00000005766	inositol 1,4,5-trisphosphate receptor type 3(LOC100008939)	-1,87
ENSOCUG00000013609	lipopolysaccharide binding protein(LBP)	-1,86
ENSOCUG00000000383	phospholipase A2 group IVE(PLA2G4E)	-1,86
ENSOCUG00000026233	HRAS-like suppressor 2(LOC100344012)	-1,85
ENSOCUG00000006001	PLAG1 zinc finger(PLAG1)	-1,85
ENSOCUG00000001029	cadherin 17(CDH17)	-1,84
ENSOCUG00000016405	EvC ciliary complex subunit 2(EVC2)	-1,84
ENSOCUG00000025809	reticulon 4 receptor-like 2(RTN4RL2)	-1,82
ENSOCUG00000000418	purinergic receptor P2Y13(P2RY13)	-1,81
ENSOCUG00000003362	laeverin(LVRN)	-1,81
ENSOCUG00000027626	CD247 molecule(CD247)	-1,81
ENSOCUG00000000386	RCAN family member 3(RCAN3)	-1,80
ENSOCUG00000010687	potassium voltage-gated channel subfamily A regulatory beta subunit 2(KCNAB2)	-1,80
ENSOCUG00000005455	NLR family pyrin domain containing 3(NLRP3)	-1,79
ENSOCUG00000012783	diacylglycerol lipase alpha(DAGLA)	-1,78
ENSOCUG00000011985	calcium voltage-gated channel subunit alpha1 A(CACNA1A)	-1,78
ENSOCUG00000000266	Wnt family member 2B(WNT2B)	-1,77
ENSOCUG00000017943	lymphocyte cytosolic protein 1(LCP1)	-1,77
ENSOCUG00000009012	Fc receptor like 5(FCRL5)	-1,77
ENSOCUG00000007382	inhibin beta E subunit(INHBE)	-1,76
ENSOCUG00000013808	G protein-coupled receptor 68(GPR68)	-1,76
ENSOCUG00000001554	tetratricopeptide repeat, ankyrin repeat and coiled-coil containing 2(TANC2)	-1,75
ENSOCUG00000006188	peripherin(PRPH)	-1,75
ENSOCUG00000003091	adenylate cyclase 7(ADCY7)	-1,75
ENSOCUG00000004172	V-set and immunoglobulin domain containing 4(VSIG4)	-1,75
ENSOCUG00000001239	sidekick cell adhesion molecule 1(SDK1)	-1,74
ENSOCUG00000007896	mast cell immunoglobulin like receptor 1(MILR1)	-1,74
ENSOCUG00000010184	trehalase(TREH)	-1,74
ENSOCUG00000013684	transmembrane protein 54(TMEM54)	-1,72
ENSOCUG00000003246	sulfatase 1(SULF1)	-1,71
ENSOCUG00000005839	immunoglobulin like domain containing receptor 2(ILDR2)	-1,71
ENSOCUG00000001150	GTPase, IMAP family member 8(GIMAP8)	-1,70
ENSOCUG00000022477	arachidonate 15-lipoxygenase(ALOX15)	-1,70
ENSOCUG00000009869	laminin subunit alpha 3(LAMA3)	-1,70
ENSOCUG00000016877	protocadherin 20(PCDH20)	-1,70
ENSOCUG00000029313	B-cell scaffold protein with ankyrin repeats 1(BANK1)	-1,69
ENSOCUG00000005931	paired immunoglobin like type 2 receptor alpha(PILRA)	-1,69
ENSOCUG00000024196	F-box protein 17(FBXO17)	-1,68
ENSOCUG00000010144	tetratricopeptide repeat domain 22(TTC22)	-1,68
ENSOCUG00000002090	Ras association domain family member 2(RASSF2)	-1,68
ENSOCUG00000002085	solute carrier family 25 member 36(SLC25A36)	-1,67
ENSOCUG00000002781	methylenetetrahydrofolate dehydrogenase (NADP+ dependent) 1-like(MTHFD1L)	-1,66
ENSOCUG00000016771	prostaglandin-endoperoxide synthase 2(PTGS2)	-1,66
ENSOCUG00000008776	F-box protein 43(FBXO43)	-1,66
ENSOCUG00000021564	toll-like receptor 12(LOC100338778)	-1,66
ENSOCUG00000009557	polypeptide N-acetylgalactosaminyltransferase 8(GALNT8)	-1,66
ENSOCUG00000001616	proprotein convertase subtilisin/kexin type 5(PCSK5)	-1,66

ENSOCUG00000026162	inhibitor of carbonic anhydrase(LOC100345698)	-1,66
ENSOCUG00000016556	G protein-coupled receptor class C group 5 member A(GPRC5A)	-1,65
ENSOCUG00000014051	interleukin 17 receptor D(IL17RD)	-1,64
ENSOCUG00000016985	membrane bound O-acyltransferase domain containing 2(MBOAT2)	-1,64
ENSOCUG00000006725	lamin tail domain containing 1(LMNTD1)	-1,63
ENSOCUG00000002052	LDL receptor related protein 4(LRP4)	-1,63
ENSOCUG00000009332	mucolipin 3(MCOLN3)	-1,63
ENSOCUG00000009932	establishment of sister chromatid cohesion N-acetyltransferase 2(ESCO2)	-1,62
ENSOCUG00000000558	myosin IF(MYO1F)	-1,62
ENSOCUG00000007857	leucine rich repeat kinase 2(LRRK2)	-1,61
ENSOCUG00000016179	thymocyte selection associated family member 2(THEMIS2)	-1,61
ENSOCUG00000012271	tumor necrosis factor superfamily member 13b(TNFSF13B)	-1,61
ENSOCUG00000001910	adhesion G protein-coupled receptor L3(ADGRL3)	-1,61
ENSOCUG00000016193	osteomodulin(OMD)	-1,61
ENSOCUG00000003763	protein tyrosine phosphatase, non-receptor type 22(PTPN22)	-1,60
ENSOCUG00000012654	phosphatidylinositol-3,4,5-trisphosphate dependent Rac exchange factor 1(PREX1)	-1,60
ENSOCUG00000013596	solute carrier family 4 member 7(SLC4A7)	-1,59
ENSOCUG00000015363	neurofascin(NFASC)	-1,58
ENSOCUG00000005208	methylenetetrahydrofolate dehydrogenase (NADP+ dependent) 2, methenyltetrahydrofolate cyclohydrolase(MTHFD2)	-1,58
ENSOCUG00000025702	cytochrome P450 4A5-like(LOC100341018)	-1,58
ENSOCUG00000003403	high affinity immunoglobulin gamma Fc receptor I(LOC100358696)	-1,58
ENSOCUG00000001235	ADAM metallopeptidase domain 19(ADAM19)	-1,57
ENSOCUG00000022873	transmembrane protein 178B(LOC100357183)	-1,57
ENSOCUG00000004392	forkhead box N3(FOXN3)	-1,56
ENSOCUG00000001426	integrin subunit beta 7(ITGB7)	-1,56
ENSOCUG00000017539	spleen associated tyrosine kinase(SYK)	-1,56
ENSOCUG00000014498	solute carrier family 16 member 12(SLC16A12)	-1,56
ENSOCUG00000026334	glycoprotein nmb(GPNMB)	-1,56
ENSOCUG00000010297	signal induced proliferation associated 1 like 2(SIPA1L2)	-1,55
ENSOCUG00000017831	platelet activating factor receptor(PTAFR)	-1,54
ENSOCUG00000028059	NLR family CARD domain containing 3(NLRC3)	-1,54
ENSOCUG00000011824	RIO kinase 1(RIOK1)	-1,54
ENSOCUG00000023225	colony stimulating factor 2 receptor alpha subunit(CSF2RA)	-1,54
ENSOCUG00000012238	NACHT and WD repeat domain containing 2(NWD2)	-1,53
ENSOCUG00000011488	Fanconi anemia complementation group I(FANCI)	-1,53
ENSOCUG00000004426	suppressor of glucose, autophagy associated 1(SOGA1)	-1,53
ENSOCUG00000005521	G protein-coupled bile acid receptor 1(GPBAR1)	-1,53
ENSOCUG00000010782	leucine rich repeat containing 8 family member C(LRRC8C)	-1,52
ENSOCUG00000000256	protein tyrosine phosphatase domain containing 1(PTPDC1)	-1,52
ENSOCUG00000009368	dual specificity phosphatase 22(DUSP22)	-1,52
ENSOCUG00000017694	keratin 23(KRT23)	-1,52
ENSOCUG00000006116	F-box protein 48(FBXO48)	-1,52
ENSOCUG00000026714	CD5 molecule like(CD5L)	-1,51
ENSOCUG00000012016	semaphorin 3D(SEMA3D)	-1,51
ENSOCUG00000002211	solute carrier family 16 member 14(SLC16A14)	-1,51
ENSOCUG00000016775	NFKB inhibitor delta(NFKBID)	-1,51
ENSOCUG00000010231	cholinergic receptor nicotinic epsilon subunit(CHRNE)	-1,51

ENSOCUG00000012566	POU class 2 associating factor 1(POU2AF1)	-1,51
ENSOCUG00000009543	potassium calcium-activated channel subfamily M alpha 1(KCNMA1)	-1,50
ENSOCUG00000001754	formin like 3(FMNL3)	-1,50
ENSOCUG00000004014	leucine rich repeat transmembrane neuronal 3(LRRTM3)	-1,50
ENSOCUG00000011397	triggering receptor expressed on myeloid cells 1(LOC100349504)	-1,50
ENSOCUG00000000215	period circadian clock 2(PER2)	-1,49
ENSOCUG00000011550	CD86 molecule(CD86)	-1,49
ENSOCUG00000015721	CD180 molecule(CD180)	-1,49
ENSOCUG00000010719	WD repeat domain 76(WDR76)	-1,49
ENSOCUG00000009751	HCK proto-oncogene, Src family tyrosine kinase(HCK)	-1,48
ENSOCUG00000006540	phospholipase C beta 2(PLCB2)	-1,48
ENSOCUG00000015778	solute carrier family 25 member 47(SLC25A47)	-1,48
ENSOCUG00000003030	PARP1 binding protein(PARPBP)	-1,48
ENSOCUG00000002338	SLAM family member 8(SLAMF8)	-1,47
ENSOCUG00000007865	T-cell surface glycoprotein CD3 gamma chain(LOC100355340)	-1,47
ENSOCUG00000029605	odorant-binding protein(LOC103347146)	-1,47
ENSOCUG00000017018	ring finger protein 152(RNF152)	-1,46
ENSOCUG00000001185	G protein-coupled receptor 149(GPR149)	-1,46
ENSOCUG00000022836	aconitate decarboxylase 1(ACOD1)	-1,46
ENSOCUG00000009070	semaphorin 3C(SEMA3C)	-1,46
ENSOCUG00000004712	GA binding protein transcription factor beta subunit 2(GABPB2)	-1,46
ENSOCUG00000029399	SH2 domain containing 1B(SH2D1B)	-1,44
ENSOCUG00000015870	centrosomal protein 89(CEP89)	-1,44
ENSOCUG00000010547	HLF, PAR bZIP transcription factor(HLF)	-1,44
ENSOCUG00000005728	G protein regulated inducer of neurite outgrowth 2(GPRIN2)	-1,43
ENSOCUG00000002192	post-GPI attachment to proteins 1(PGAP1)	-1,43
ENSOCUG00000017147	CDP-diacylglycerol synthase 1(CDS1)	-1,43
ENSOCUG00000005167	angiopoietin like 1(ANGPTL1)	-1,43
ENSOCUG00000004033	chromosome 16 open reading frame, human C1orf74(C16H1orf74)	-1,43
ENSOCUG00000025681	leucine rich repeat neuronal 4(LRRN4)	-1,43
ENSOCUG00000009651	DnaJ heat shock protein family (Hsp40) member B7(DNAJB7)	-1,42
ENSOCUG00000010621	Bruton tyrosine kinase(BTK)	-1,42
ENSOCUG00000002197	integrin subunit beta 8(ITGB8)	-1,42
ENSOCUG00000024091	solute carrier family 22 member 8(SLC22A8)	-1,42
ENSOCUG00000002480	SLA class II histocompatibility antigen, DQ haplotype D alpha chain(LOC100343144)	-1,42
ENSOCUG00000014953	spectrin repeat containing nuclear envelope family member 3(SYNE3)	-1,42
ENSOCUG00000016192	cysteinyl leukotriene receptor 1(CYSLTR1)	-1,42
ENSOCUG00000010728	apolipoprotein A1(APOA1)	-1,42
ENSOCUG00000002231	very low density lipoprotein receptor(VLDLR)	-1,42
ENSOCUG00000006043	ubiquitin specific peptidase 13 (isopeptidase T-3)(USP13)	-1,42
ENSOCUG00000006029	alpha-2-macroglobulin(LOC100349077)	-1,41
ENSOCUG00000010941	carboxypeptidase X, M14 family member 2(CPXM2)	-1,41
ENSOCUG00000009801	cysteine rich hydrophobic domain 1(CHIC1)	-1,41
ENSOCUG00000015479	POU class 2 homeobox 1(POU2F1)	-1,41
ENSOCUG00000022385	nidogen-1(LOC103347221)	-1,40
ENSOCUG00000024216	myosin IG(MYO1G)	-1,40
ENSOCUG00000000830	dedicator of cytokinesis 2(DOCK2)	-1,40
ENSOCUG00000027586	EPM2A interacting protein 1(EPM2AIP1)	-1,39

ENSOCUG00000008236	lipoprotein lipase(LPL)	-1,39
ENSOCUG00000023075	plectin(LOC100349129)	-1,39
ENSOCUG00000011752	pygopus family PHD finger 1(PYGO1)	-1,39
ENSOCUG00000011054	phosphatidylinositol-4,5-bisphosphate 3-kinase catalytic subunit delta(PIK3CD)	-1,39
ENSOCUG00000006708	actin filament associated protein 1(AFAP1)	-1,39
ENSOCUG00000005885	immunoglobulin superfamily member 10(IGSF10)	-1,38
ENSOCUG00000010659	ADAM metallopeptidase domain 23(ADAM23)	-1,38
ENSOCUG00000009276	myosin binding protein C, slow type(MYBPC1)	-1,37
ENSOCUG00000001587	integrin alpha-M(LOC100351865)	-1,37
ENSOCUG00000017504	synaptotagmin 9(SYT9)	-1,37
ENSOCUG00000015877	secreted frizzled related protein 4(SFRP4)	-1,37
ENSOCUG00000014633	PBX/knotted 1 homeobox 2(PKNOX2)	-1,37
ENSOCUG00000015623	plexin A3(PLXNA3)	-1,37
ENSOCUG00000007763	calcium voltage-gated channel auxiliary subunit alpha2delta 1(CACNA2D1)	-1,37
ENSOCUG00000002018	UHRF1 binding protein 1(UHRF1BP1)	-1,37
ENSOCUG00000014786	INO80 complex subunit D(INO80D)	-1,36
ENSOCUG00000000960	sidekick cell adhesion molecule 2(SDK2)	-1,36
ENSOCUG00000004804	feline leukemia virus subgroup C cellular receptor 1(FLVCR1)	-1,35
ENSOCUG00000003405	major facilitator superfamily domain containing 6(MFSD6)	-1,35
ENSOCUG00000029469	phosphatidylinositol 3,4,5-trisphosphate 3-phosphatase TPTE2(LOC100346342)	-1,35
ENSOCUG00000006445	frizzled class receptor 4(FZD4)	-1,35
ENSOCUG00000016347	phosphoethanolamine/phosphocholine phosphatase(PHOSPHO1)	-1,35
ENSOCUG00000000534	lymphocyte cytosolic protein 2(LCP2)	-1,34
ENSOCUG00000000136	basonuclin 1(BNC1)	-1,34
ENSOCUG00000003720	dual specificity phosphatase 4(DUSP4)	-1,34
ENSOCUG00000009774	lysosomal protein transmembrane 5(LAPTM5)	-1,34
ENSOCUG00000002530	prostaglandin-endoperoxide synthase 1(PTGS1)	-1,34
ENSOCUG00000014567	acyloxyacyl hydrolase(AOAH)	-1,33
ENSOCUG00000004988	lysyl oxidase like 4(LOXL4)	-1,33
ENSOCUG00000004275	semaphorin 5A(SEMA5A)	-1,32
ENSOCUG00000017204	DAB1, reelin adaptor protein(DAB1)	-1,32
ENSOCUG00000007759	ubiquitin specific peptidase 2(USP2)	-1,31
ENSOCUG00000022167	complement C5a receptor 1(C5AR1)	-1,31
ENSOCUG00000029622	SLAM family member 9(LOC103350037)	-1,30
ENSOCUG00000000718	solute carrier family 1 member 2(SLC1A2)	-1,30
ENSOCUG00000015570	FRY microtubule binding protein(FRY)	-1,30
ENSOCUG00000003701	toll like receptor 10(TLR10)	-1,30
ENSOCUG00000024364	MIER family member 2(MIER2)	-1,30
ENSOCUG00000009379	MIS18 kinetochore protein A(MIS18A)	-1,29
ENSOCUG00000013916	protein kinase C beta(PRKCB)	-1,29
ENSOCUG00000001920	G protein-coupled receptor class C group 5 member B(GPRC5B)	-1,29
ENSOCUG00000002071	glycerol-3-phosphate acyltransferase 4(GPAT4)	-1,29
ENSOCUG00000029457	hamartin(LOC103346667)	-1,29
ENSOCUG00000007916	glucosaminyl (N-acetyl) transferase 1, core 2(GCNT1)	-1,28
ENSOCUG00000012990	LON peptidase N-terminal domain and ring finger 3(LONRF3)	-1,28
ENSOCUG00000011956	cadherin related family member 2(CDHR2)	-1,28
ENSOCUG00000006113	pleckstrin(PLEK)	-1,28
ENSOCUG00000010101	interferon regulatory factor 5(IRF5)	-1,28

ENSOCUG00000013467	ring finger protein 150(RNF150)	-1,28
ENSOCUG00000012402	uroplakin-3b(LOC100355286)	-1,28
ENSOCUG00000023355	myosin VIIA(MYO7A)	-1,27
ENSOCUG00000002553	elastin microfibril interfacer 2(EMILIN2)	-1,27
ENSOCUG00000026567	histocompatibility antigen DM heterodimer light chain-like(RLA-DMB)	-1,27
ENSOCUG00000013517	NCK associated protein 1 like(NCKAP1L)	-1,27
ENSOCUG00000024108	potassium voltage-gated channel subfamily C member 3(LOC100338015)	-1,27
ENSOCUG00000025513	insulin like growth factor binding protein 5(IGFBP5)	-1,27
ENSOCUG00000014740	solute carrier family 37 member 2(SLC37A2)	-1,26
ENSOCUG00000029435	ligand-dependent corepressor(LOC100343926)	-1,26
ENSOCUG00000026260	solute carrier family 6 member 8(SLC6A8)	-1,26
ENSOCUG00000013550	upstream transcription factor family member 3(USF3)	-1,26
ENSOCUG00000009436	cyclic nucleotide gated channel alpha 2(CNGA2)	-1,26
ENSOCUG00000012620	Werner syndrome RecQ like helicase(WRN)	-1,26
ENSOCUG00000010987	transmembrane protein 26(TMEM26)	-1,26
ENSOCUG00000009050	reelin(RELN)	-1,26
ENSOCUG00000000401	hexokinase 3(HK3)	-1,26
ENSOCUG00000000763	phospholipase A2 group VII(PLA2G7)	-1,26
ENSOCUG00000027817	protein phosphatase 1 regulatory subunit 16B(PPP1R16B)	-1,25
ENSOCUG00000013120	polyhomeotic homolog 3(PHC3)	-1,25
ENSOCUG00000011691	serine/threonine kinase 17b(STK17B)	-1,25
ENSOCUG00000009478	Rho GTPase activating protein 31(ARHGAP31)	-1,25
ENSOCUG00000000007	GLI pathogenesis related 2(GLIPR2)	-1,25
ENSOCUG00000006803	ectonucleoside triphosphate diphosphohydrolase 5(ENTPD5)	-1,25
ENSOCUG00000016651	AXL receptor tyrosine kinase(AXL)	-1,25
ENSOCUG00000003221	lysine demethylase 7A(KDM7A)	-1,25
ENSOCUG00000014964	ATP binding cassette subfamily B member 4(ABCB4)	-1,25
ENSOCUG00000014482	plexin domain containing 2(PLXDC2)	-1,24
ENSOCUG00000017793	CD38 molecule(CD38)	-1,24
ENSOCUG00000028137	indian hedgehog(IHH)	-1,24
ENSOCUG00000008922	FGR proto-oncogene, Src family tyrosine kinase(FGR)	-1,24
ENSOCUG00000014795	insulin like growth factor 1 receptor(IGF1R)	-1,24
ENSOCUG00000008600	formyl peptide receptor 1(FPR1)	-1,24
ENSOCUG00000021438	adhesion G protein-coupled receptor E5(ADGRE5)	-1,23
ENSOCUG00000015483	fatty acid binding protein 4(FABP4)	-1,23
ENSOCUG00000001711	trophinin(TRO)	-1,23
ENSOCUG00000024991	alpha kinase 1(ALPK1)	-1,23
ENSOCUG00000009447	oxysterol binding protein like 7(OSBPL7)	-1,23
ENSOCUG00000002306	protease, serine, 8(PRSS8)	-1,22
ENSOCUG00000006497	peroxidasin(PXDN)	-1,22
ENSOCUG00000010918	FER tyrosine kinase(FER)	-1,22
ENSOCUG00000009042	CNKSR family member 3(CNKSR3)	-1,22
ENSOCUG00000012372	lipin 3(LPIN3)	-1,21
ENSOCUG00000015909	heparin binding EGF like growth factor(HBEGF)	-1,21
ENSOCUG00000002566	sex hormone binding globulin(SHBG)	-1,21
ENSOCUG00000011309	plexin domain containing 1(PLXDC1)	-1,21
ENSOCUG00000008291	bridging integrator 2(BIN2)	-1,21
ENSOCUG00000022214	acyl-coenzyme A thioesterase 4(LOC100343752)	-1,21

ENSOCUG00000011981	protein tyrosine phosphatase, receptor type C(PTPRC)	-1,21
ENSOCUG00000023778	CD300a molecule(CD300A)	-1,21
ENSOCUG00000001006	tachykinin receptor 1(TACR1)	-1,21
ENSOCUG00000013642	dedicator of cytokinesis 11(DOCK11)	-1,21
ENSOCUG00000016755	UDP-glucuronosyltransferase 2A3(LOC100359229)	-1,20
ENSOCUG00000008757	myoferlin(MYOF)	-1,20
ENSOCUG00000003924	apolipoprotein L3(LOC100350735)	-1,20
ENSOCUG00000010012	adenylate kinase 4(AK4)	-1,20
ENSOCUG00000010637	minichromosome maintenance complex component 5(MCM5)	-1,20
ENSOCUG00000016343	regulator of telomere elongation helicase 1(RTEL1)	-1,19
ENSOCUG00000002613	versican(VCAN)	-1,19
ENSOCUG00000024739	zinc finger BED-type containing 6(ZBED6)	-1,19
ENSOCUG00000025736	apolipoprotein B mRNA editing enzyme catalytic subunit 1(APOBEC1)	-1,19
ENSOCUG00000016115	ras-related C3 botulinum toxin substrate 2 (rho family, small GTP binding protein Rac2)(RAC2)	-1,19
ENSOCUG00000023116	protein argonaute-1(LOC100347349)	-1,19
ENSOCUG00000006571	OTU deubiquitinase 3(OTUD3)	-1,19
ENSOCUG00000008656	guanylate-binding protein 5(LOC100349257)	-1,19
ENSOCUG00000011935	PTC7 protein phosphatase homolog(PPTC7)	-1,19
ENSOCUG00000026383	aldo-keto reductase family 1 member B10(AKR1B10)	-1,19
ENSOCUG00000000786	EPH receptor A2(EPHA2)	-1,19
ENSOCUG00000007922	ST8 alpha-N-acetyl-neuraminide alpha-2,8-sialyltransferase 4(ST8SIA4)	-1,19
ENSOCUG00000004618	CKLF like MARVEL transmembrane domain containing 4(CMTM4)	-1,19
ENSOCUG00000016280	C-X-C motif chemokine ligand 10(CXCL10)	-1,18
ENSOCUG00000017892	zinc finger CCCH-type containing, antiviral 1(ZC3HAV1)	-1,18
ENSOCUG00000024065	nuclear prelamin A recognition factor(NARF)	-1,18
ENSOCUG00000005237	protocadherin 17(PCDH17)	-1,18
ENSOCUG00000001289	septin 11(SEPT11)	-1,18
ENSOCUG00000029190	placenta specific 8(PLAC8)	-1,18
ENSOCUG00000028062	GTPase, IMAP family member 1(GIMAP1)	-1,18
ENSOCUG00000022216	collectin subfamily member 12(COLEC12)	-1,18
ENSOCUG00000013760	neuralized E3 ubiquitin protein ligase 1(NEURL1)	-1,18
ENSOCUG00000014820	cytochrome b reductase 1(LOC100346448)	-1,18
ENSOCUG00000010563	TAP binding protein(TAPBP)	-1,17
ENSOCUG00000002485	HLA class II histocompatibility antigen, DQ beta 1 chain(LOC100351163)	-1,17
ENSOCUG00000001594	solute carrier family 16 member 6(SLC16A6)	-1,17
ENSOCUG00000000575	hepatitis A virus cellular receptor 1(HAVCR1)	-1,17
ENSOCUG00000012814	TROVE domain family member 2(TROVE2)	-1,16
ENSOCUG00000012148	inositol 1,4,5-trisphosphate receptor type 1(ITPR1)	-1,16
ENSOCUG00000000715	CD44 molecule (Indian blood group)(CD44)	-1,16
ENSOCUG00000007286	collagen type IV alpha 4 chain(COL4A4)	-1,15
ENSOCUG00000004436	insulin receptor(INSR)	-1,15
ENSOCUG00000011764	LIM domain containing preferred translocation partner in lipoma(LPP)	-1,15
ENSOCUG00000014976	ERCC excision repair 4, endonuclease catalytic subunit(ERCC4)	-1,15
ENSOCUG00000014646	fasciculation and elongation protein zeta 1(FEZ1)	-1,15
ENSOCUG00000001481	family with sequence similarity 65 member B(FAM65B)	-1,14
ENSOCUG00000004942	protein kinase AMP-activated catalytic subunit alpha 2(PRKAA2)	-1,14
ENSOCUG00000025241	liver carboxylesterase 2-like(LOC100357214)	-1,14

ENSOCUG00000009311	CD84 molecule(CD84)	-1,14
ENSOCUG00000006719	lysophosphatidylcholine acyltransferase 2(LPCAT2)	-1,14
ENSOCUG00000002281	cytochrome P450 2U1(LOC100353947)	-1,13
ENSOCUG00000015138	complement component 1, q subcomponent, C chain(C1QC)	-1,13
ENSOCUG00000012906	SPARC related modular calcium binding 2(SMOC2)	-1,13
ENSOCUG00000008688	cilia and flagella associated protein 43(CFAP43)	-1,13
ENSOCUG00000007380	inhibin beta C subunit(INHBC)	-1,13
ENSOCUG00000004808	Cbl proto-oncogene(CBL)	-1,13
ENSOCUG00000011350	spondin 1(SPON1)	-1,13
ENSOCUG00000001060	ankyrin repeat domain 13A(ANKRD13A)	-1,13
ENSOCUG00000002935	family with sequence similarity 46 member C(FAM46C)	-1,13
ENSOCUG00000012779	G protein-coupled receptor 137B(GPR137B)	-1,12
ENSOCUG00000000142	coagulation factor VIII(F8)	-1,12
ENSOCUG00000023891	DNA damage inducible 1 homolog 2(DDI2)	-1,12
ENSOCUG00000026291	arrestin beta 1(ARRB1)	-1,11
ENSOCUG00000003729	nuclear factor related to kappaB binding protein(NFRKB)	-1,11
ENSOCUG00000006350	solute carrier family 12 member 4(SLC12A4)	-1,11
ENSOCUG00000013527	abhydrolase domain containing 2(ABHD2)	-1,10
ENSOCUG00000005112	F-box protein 32(FBXO32)	-1,10
ENSOCUG00000000427	peptidase M20 domain containing 1(PM20D1)	-1,09
ENSOCUG00000027006	UDP-glucuronosyltransferase 2B16-like(LOC100340258)	-1,08
ENSOCUG00000006566	X-linked Kx blood group(XK)	-1,08
ENSOCUG00000026015	liver carboxylesterase 2-like(LOC100358248)	-1,07
ENSOCUG00000011544	integrin subunit beta 2(ITGB2)	-1,07
ENSOCUG00000001497	fatty acid binding protein 7(FABP7)	1,08
ENSOCUG00000015313	cytochrome c oxidase protein 20 homolog(LOC100349428)	1,10
ENSOCUG00000014585	zinc finger DHHC-type containing 2(ZDHHC2)	1,12
ENSOCUG00000000688	F-box protein 27(FBXO27)	1,13
ENSOCUG00000004733	prostaglandin-E(2) 9-reductase-like(PGER2)	1,13
ENSOCUG00000005985	clusterin(CLU)	1,13
ENSOCUG00000024462	60S ribosomal protein L27(LOC100356974)	1,14
ENSOCUG00000014776	solute carrier family 26 member 8(SLC26A8)	1,17
ENSOCUG00000017350	glycosyltransferase 1 domain containing 1(GLT1D1)	1,17
ENSOCUG00000002702	radical S-adenosyl methionine domain containing 1(RSAD1)	1,18
ENSOCUG00000024412	serum amyloid A-4 protein(LOC100342244)	1,20
ENSOCUG00000003858	glycine N-methyltransferase(GNMT)	1,21
ENSOCUG00000029478	fragile X mental retardation 1 neighbor(FMR1NB)	1,23
ENSOCUG00000006681	ATP synthase, H+ transporting, mitochondrial Fo complex subunit F6(ATP5J)	1,24
ENSOCUG00000008329	ADAM metallopeptidase with thrombospondin type 1 motif 19(ADAMTS19)	1,24
ENSOCUG00000007493	isopentenyl-diphosphate Delta-isomerase 1(LOC100343510)	1,25
ENSOCUG00000016551	C1q and tumor necrosis factor related protein 7(C1QTNF7)	1,28
ENSOCUG00000009195	C-type lectin domain family 4 member E(CLEC4E)	1,30
ENSOCUG00000029235	metallothionein-1A(LOC100343802)	1,31
ENSOCUG00000004160	family with sequence similarity 107 member A(FAM107A)	1,34
ENSOCUG00000012902	glutathione S-transferase Yc(LOC100353428)	1,34
ENSOCUG00000015329	matrix metallopeptidase 7(MMP7)	1,36
ENSOCUG00000008193	actin, alpha 2, smooth muscle, aorta(ACTA2)	1,37
ENSOCUG00000023425	alpha-fetoprotein(LOC103350776)	1,37

ENSOCUG00000014505	tribbles pseudokinase 3(TRIB3)	1,37
ENSOCUG00000009825	biphenyl hydrolase like(BPHL)	1,40
ENSOCUG00000009769	immunoglobulin superfamily member 1(IGSF1)	1,40
ENSOCUG00000007890	SCO-spondin(SSPO)	1,41
ENSOCUG00000016210	RAB15 effector protein(REP15)	1,41
ENSOCUG00000010814	malic enzyme 1(ME1)	1,41
ENSOCUG00000006139	semaphorin 5B(SEMA5B)	1,45
ENSOCUG00000015111	glutamate ionotropic receptor NMDA type subunit 2B(GRIN2B)	1,50
ENSOCUG00000008104	growth differentiation factor 6(GDF6)	1,52
ENSOCUG00000008571	myomesin 1(MYOM1)	1,52
ENSOCUG00000016772	DNA damage inducible transcript 4(DDIT4)	1,53
ENSOCUG00000009725	acyl-CoA wax alcohol acyltransferase 1(AWAT1)	1,54
ENSOCUG00000002542	solute carrier family 22 member 2(SLC22A2)	1,62
ENSOCUG00000015619	calbindin 2(CALB2)	1,64
ENSOCUG00000015771	squalene epoxidase(SQLE)	1,70
ENSOCUG00000008109	chromosome 3 open reading frame, human C8orf34(C3H8orf34)	1,70
ENSOCUG00000024372	trace amine-associated receptor 4(TAAR4)	1,71
ENSOCUG00000007266	sodium channel protein type 1 subunit alpha(LOC100009591)	1,75
ENSOCUG00000017620	serum amyloid protein A(LOC100009259)	1,79
ENSOCUG00000013412	C-C motif chemokine 7(LOC103351517)	1,81
ENSOCUG00000004297	otoancorin(OTOA)	1,81
ENSOCUG00000010549	monocyte to macrophage differentiation associated(MMD)	1,85
ENSOCUG00000013324	hypocretin neuropeptide precursor(HCRT)	1,86
ENSOCUG00000015777	potassium voltage-gated channel subfamily D member 1(KCND1)	1,96
ENSOCUG00000012939	ectonucleotide pyrophosphatase/phosphodiesterase 5 (putative)(ENPP5)	2,03
ENSOCUG00000013934	coiled-coil domain containing 189(CCDC189)	2,07
ENSOCUG00000025992	butyrophilin subfamily 1 member A1-like(LOC100344369)	2,09
ENSOCUG00000007327	contactin associated protein 1(CNTNAP1)	2,10
ENSOCUG00000027125	ATP-binding cassette sub-family A member 3(LOC100353012)	2,31
ENSOCUG00000029754	TSSK6 activating cochaperone(TSACC)	2,32
ENSOCUG00000021120	mucin 15, cell surface associated(MUC15)	2,32
ENSOCUG00000025107	membrane associated ring-CH-type finger 4(MARCH4)	2,33
ENSOCUG00000027492	zymogen granule membrane protein 16(LOC100346271)	2,36
ENSOCUG00000005346	tudor domain containing 15(TDRD15)	2,41
ENSOCUG00000006124	leukemia NUP98 fusion partner 1(LNP1)	2,47
ENSOCUG00000026936	delta like non-canonical Notch ligand 1(DLK1)	2,51
ENSOCUG00000001309	isthmin 1(ISM1)	2,52
ENSOCUG00000002462	solute carrier family 24 member 2(SLC24A2)	2,55
ENSOCUG00000013373	sperm tail PG-rich repeat containing 1(STPG1)	2,64
ENSOCUG00000011025	contactin 4(CNTN4)	2,67
ENSOCUG00000009438	claudin 10(CLDN10)	2,80
ENSOCUG00000026267	aldo-keto reductase family 1, member C1 (dihydrodiol dehydrogenase 1; 20-alpha (3-alpha)-hydroxysteroid dehydrogenase)(AKR1C5)	2,83
ENSOCUG00000003017	glutamate rich 3(ERICH3)	2,98
ENSOCUG00000008391	GDNF family receptor alpha 3(GFRA3)	2,99
ENSOCUG00000029690	zymogen granule membrane protein 16(LOC100350057)	3,03
ENSOCUG00000021940	putative alpha-1-antitrypsin-related protein(LOC100358746)	3,08
ENSOCUG00000003953	chromosome 13 open reading frame, human C1orf146(C13H1orf146)	3,17

ENSOCUG00000015301	sodium voltage-gated channel beta subunit 1(SCN1B)	3,27
ENSOCUG00000029599	killer cell lectin like receptor B1(KLRB1)	3,32
ENSOCUG00000016764	myelin transcription factor 1 like(MYT1L)	3,51
ENSOCUG00000027771	zymogen granule membrane protein 16-like(LOC100359023)	3,52
ENSOCUG00000017790	steroid 17-alpha-hydroxylase/17,20 lyase(LOC100346394)	3,58
ENSOCUG00000008181	carboxypeptidase E(CPE)	3,60
ENSOCUG00000012592	ankyrin repeat and SOCS box containing 5(ASB5)	3,65
ENSOCUG00000005979	chromosome 15 open reading frame, human C4orf17(C15H4orf17)	3,66
ENSOCUG00000022883	glycerophosphodiester phosphodiesterase domain containing 3(GDPD3)	3,66
ENSOCUG00000004205	protease, serine 35(PRSS35)	3,95
ENSOCUG00000029412	zymogen granule membrane protein 16-like(LOC100352055)	4,02
ENSOCUG00000003021	ceramide kinase like(CERKL)	4,59
ENSOCUG00000027755	putative spermatogenesis-associated protein 31D3(LOC100355671)	4,81
ENSOCUG00000017941	platelet microbicidal protein 1(LOC100008921)	5,67
ENSOCUG00000000901	seizure related 6 homolog like 2(SEZ6L2)	6,23
ENSOCUG00000029318	zymogen granule membrane protein 16-like(LOC100351054)	6,75
ENSOCUG00000015803	tubulointerstitial nephritis antigen(TINAG)	7,38
ENSOCUG00000029735	carboxypeptidase E(LOC100343425)	10,72
ENSOCUG00000000199	deuterosome assembly protein 1(DEUP1)	< -0,01
ENSOCUG00000000315	glutamate ionotropic receptor AMPA type subunit 3(GRIA3)	< -0,01
ENSOCUG00000000341	regulator of G-protein signaling 6(RGS6)	< -0,01
ENSOCUG00000000774	protocadherin 9(PCDH9)	< -0,01
ENSOCUG00000001022	solute carrier family 13 member 1(SLC13A1)	< -0,01
ENSOCUG00000001703	cation channel sperm associated 3(CATSPER3)	< -0,01
ENSOCUG00000002830	tensin 4(TNS4)	< -0,01
ENSOCUG00000003724	multiple EGF like domains 10(MEGF10)	< -0,01
ENSOCUG00000004480	cysteine and glycine rich protein 3(CSRP3)	< -0,01
ENSOCUG00000006146	paired box 7(PAX7)	< -0,01
ENSOCUG00000006307	translin associated factor X interacting protein 1(TSNAXIP1)	< -0,01
ENSOCUG00000006728	myomesin 3(MYOM3)	< -0,01
ENSOCUG00000011270	CD1b molecule(CD1B)	< -0,01
ENSOCUG00000014011	glutamate receptor interacting protein 1(GRIP1)	< -0,01
ENSOCUG00000014187	KIAA1024 ortholog(KIAA1024)	< -0,01
ENSOCUG00000015507	sushi domain containing 5(SUSD5)	< -0,01
ENSOCUG00000016053	RAB44, member RAS oncogene family(RAB44)	< -0,01
ENSOCUG00000016787	serpin family B member 5(SERPINB5)	< -0,01
ENSOCUG00000016970	dynein heavy chain domain 1(DNHD1)	< -0,01
ENSOCUG00000022989	ring finger protein 32(RNF32)	< -0,01
ENSOCUG00000027297	EF-hand calcium binding domain 8(EFCAB8)	< -0,01
ENSOCUG00000027575	potassium voltage-gated channel subfamily H member 2(KCNH2)	< -0,01
ENSOCUG00000001040	nei like DNA glycosylase 3(NEIL3)	< -0,02
ENSOCUG00000001986	phospholipase C delta 4(PLCD4)	< -0,02
ENSOCUG00000005563	lysosomal associated membrane protein 3(LAMP3)	< -0,02
ENSOCUG00000006290	leucine-rich repeat and fibronectin type-III domain-containing protein 5(LOC100349270)	< -0,02
ENSOCUG00000009933	germinal center associated signaling and motility(GCSAM)	< -0,02
ENSOCUG00000011975	zinc finger and BTB domain containing 8B(ZBTB8B)	< -0,02
ENSOCUG00000015565	potassium voltage-gated channel subfamily J member 5(KCNJ5)	< -0,02

ENSOCUG00000021520	RASD family member 2(RASD2)	< -0,02
ENSOCUG00000021659	arachidonate 12-lipoxygenase, 12S type(ALOX12)	< -0,02
ENSOCUG00000023547	tubulin alpha-3 chain(LOC100350967)	< -0,02
ENSOCUG00000026674	granzyme A(LOC100346200)	< -0,02
ENSOCUG00000027210	Fc fragment of IgG binding protein(FCGBP)	< -0,02
ENSOCUG00000027997	tescalcin(TESC)	< -0,02
ENSOCUG00000000883	transketolase-like 1(TKTL1)	< -0,03
ENSOCUG00000006061	thymocyte selection associated(THEMIS)	< -0,03
ENSOCUG00000010009	eosinophil peroxidase(EPX)	< -0,03
ENSOCUG00000012981	tropomodulin 2(TMOD2)	< -0,03
ENSOCUG00000023285	trophinin associated protein(TROAP)	< -0,03
ENSOCUG00000029569	keratin 17(KRT17)	< -0,03
ENSOCUG00000006579	sphingosine kinase 1(SPHK1)	< -0,04
ENSOCUG00000007567	solute carrier family 38 member 11(SLC38A11)	< -0,04
ENSOCUG00000011561	olfactory receptor 1J1(LOC100345888)	< -0,04
ENSOCUG00000016659	embigin(EMB)	< -0,04
ENSOCUG00000026537	membrane spanning 4-domains A18(MS4A18)	< -0,04
ENSOCUG00000000194	early growth response 3(EGR3)	< -0,05
ENSOCUG00000000793	calcineurin like EF-hand protein 2(CHP2)	< -0,05
ENSOCUG00000001178	family with sequence similarity 78 member B(FAM78B)	< -0,05
ENSOCUG00000011516	adenylate kinase 7(AK7)	< -0,05
ENSOCUG00000014829	synaptotagmin 1(SYT1)	< -0,05
ENSOCUG00000023952	netrin G1(NTNG1)	< -0,05
ENSOCUG00000004004	family with sequence similarity 135 member B(FAM135B)	< -0,06
ENSOCUG00000004369	chromosome 3 open reading frame, human C5orf46(C3H5orf46)	< -0,06
ENSOCUG00000008657	cell growth regulator with EF-hand domain 1(CGREF1)	< -0,06
ENSOCUG00000027283	solute carrier family 25 member 45(SLC25A45)	< -0,06
ENSOCUG00000009843	SPARC/osteonectin, cwcv and kazal like domains proteoglycan 1(SPOCK1)	< -0,07
ENSOCUG00000014008	chromatin assembly factor 1 subunit B(CHAF1B)	< -0,07
ENSOCUG00000004603	retinal degeneration 3(RD3)	< -0,08
ENSOCUG00000013829	potassium channel tetramerization domain containing 4(KCTD4)	< -0,08
ENSOCUG00000009695	EPS8 like 3(EPS8L3)	< -0,09
ENSOCUG00000017737	purinergic receptor P2X 2(P2RX2)	< -0,11
ENSOCUG00000006014	phosphodiesterase 6H(PDE6H)	< -0,14
ENSOCUG00000003089	proteoglycan 3, pro eosinophil major basic protein 2(PRG3)	< -0,32
ENSOCUG00000003984	solute carrier family 8 member A3(SLC8A3)	> 0,01
ENSOCUG00000006597	calmegin(CLGN)	> 0,01
ENSOCUG00000014837	solute carrier family 39 member 12(SLC39A12)	> 0,01
ENSOCUG00000017128	neuromedin U(NMU)	> 0,01
ENSOCUG00000017476	epoxide hydrolase 4(EPHX4)	> 0,01
ENSOCUG00000021711	protein phosphatase 1 regulatory inhibitor subunit 1B(PPP1R1B)	> 0,01
ENSOCUG00000027106	hyaluronan synthase 2(HAS2)	> 0,01
ENSOCUG00000004691	prominin 2(PROM2)	> 0,02
ENSOCUG00000004847	growth associated protein 43(GAP43)	> 0,02
ENSOCUG00000011440	glutamate ionotropic receptor delta type subunit 2(GRID2)	> 0,02
ENSOCUG00000012760	zinc finger and BTB domain containing 32(ZBTB32)	> 0,02
ENSOCUG00000015813	neuromedin U receptor 1(NMUR1)	> 0,02
ENSOCUG00000024593	aldehyde oxidase 4(AOX4)	> 0,02

ENSOCUG00000028202	family with sequence similarity 178 member B(FAM178B)	> 0,02
ENSOCUG00000001290	synaptotagmin 12(SYT12)	> 0,03
ENSOCUG00000004746	spermatogenesis associated 16(SPATA16)	> 0,03
ENSOCUG00000002301	cyclin B1 interacting protein 1(CCNB1IP1)	> 0,04
ENSOCUG00000002624	Nik related kinase(NRK)	> 0,07
ENSOCUG00000004047	spindle and kinetochore associated complex subunit 3(SKA3)	> 0,08
ENSOCUG00000029446	tripartite motif containing 60(TRIM60)	> 0,12
ENSOCUG00000003481	RERG like(RERGL)	> 0,14
ENSOCUG00000026203	complement C1q like 1(C1QL1)	> 0,14

Supplementary Table S12. Functional analysis of the differentially expressed transcripts in liver tissue between vitrified-transferred progeny and that naturally-conceived in F3.

Category	Term	Count	P-Value
BP	chemotaxis	13	0,000
BP	immune response	18	0,000
BP	inflammatory response	16	0,000
BP	arachidonic acid metabolic process	5	0,001
BP	integrin-mediated signaling pathway	9	0,002
BP	cell adhesion	13	0,002
BP	linoleic acid metabolic process	4	0,003
BP	lipoxygenase pathway	4	0,003
BP	hematopoietic progenitor cell differentiation	9	0,003
BP	regulation of autophagy	5	0,009
BP	sperm capacitation	4	0,011
BP	regulation of ventricular cardiac muscle cell membrane repolarization	4	0,015
BP	T cell receptor signaling pathway	6	0,015
BP	B cell receptor signaling pathway	5	0,018
BP	ventral spinal cord development	3	0,018
BP	lipid transport	5	0,024
BP	negative regulation of tumor necrosis factor production	5	0,024
BP	positive regulation of defense response to virus by host	9	0,025
BP	locomotion involved in locomotory behavior	3	0,027
BP	glial cell differentiation	3	0,027
BP	regulation of calcium ion-dependent exocytosis	3	0,027
BP	neuronal action potential propagation	3	0,027
BP	positive regulation of neutrophil chemotaxis	4	0,027
BP	neutrophil chemotaxis	5	0,027
BP	phagocytosis, recognition	3	0,036
BP	GTP metabolic process	3	0,036
BP	transmembrane transport	8	0,037
BP	positive regulation of cytokine secretion	4	0,038
BP	positive regulation of type I interferon production	3	0,047
BP	skeletal muscle fiber development	4	0,051
BP	positive regulation of gene expression	11	0,052
BP	signal transduction	14	0,053
BP	positive regulation of vascular endothelial growth factor production	4	0,058
BP	receptor clustering	3	0,059
BP	cellular response to exogenous dsRNA	3	0,059
BP	behavioral response to pain	3	0,059
BP	response to oxidative stress	6	0,063
BP	defense response to virus	7	0,070
BP	homophilic cell adhesion via plasma membrane adhesion molecules	7	0,070
BP	negative regulation of T cell activation	3	0,071
BP	peripheral nervous system development	3	0,071
BP	regulation of signal transduction	3	0,071

BP	calcium-mediated signaling	4	0,082
BP	sodium ion transport	4	0,082
BP	secretory granule localization	2	0,088
BP	positive regulation of hydrolase activity	2	0,088
BP	positive regulation of toll-like receptor 7 signaling pathway	2	0,088
BP	Fc-epsilon receptor signaling pathway	2	0,088
BP	enzyme linked receptor protein signaling pathway	2	0,088
BP	positive regulation of toll-like receptor 9 signaling pathway	2	0,088
BP	cyclooxygenase pathway	2	0,088
BP	regulation of NADP metabolic process	2	0,088
BP	cell-cell adhesion mediated by cadherin	2	0,088
BP	positive regulation of protein kinase activity	4	0,090
BP	positive regulation of cell migration	8	0,091
BP	xenophagy	7	0,093
BP	innate immune response	8	0,095
BP	transmission of nerve impulse	3	0,098
BP	neuromuscular synaptic transmission	3	0,098
BP	heterotypic cell-cell adhesion	3	0,098
BP	long-term synaptic potentiation	4	0,099
BP	regulation of heart rate by cardiac conduction	4	0,099
CC	integral component of membrane	212	0,000
CC	extracellular matrix	13	0,000
CC	cell surface	26	0,000
CC	zymogen granule membrane	5	0,005
CC	receptor complex	10	0,008
CC	intracellular membrane-bounded organelle	8	0,020
CC	extracellular space	38	0,033
CC	apical plasma membrane	12	0,034
CC	brush border membrane	4	0,039
CC	proteinaceous extracellular matrix	11	0,050
CC	extracellular exosome	96	0,051
CC	node of Ranvier	3	0,053
CC	voltage-gated potassium channel complex	6	0,056
CC	neuronal cell body	9	0,056
CC	MHC class II protein complex	3	0,077
CC	phagocytic cup	3	0,077
MF	calcium ion binding	39	0,000
MF	peroxidase activity	5	0,000
MF	carbohydrate binding	9	0,006
MF	non-membrane spanning protein tyrosine kinase activity	6	0,006
MF	arachidonate 12-lipoxygenase activity	3	0,009
MF	glycoprotein binding	6	0,026
MF	hyaluronic acid binding	4	0,027
MF	3',5'-cyclic-nucleotide phosphodiesterase activity	3	0,029
MF	protein tyrosine phosphatase activity	8	0,036
MF	transmembrane signaling receptor activity	5	0,036
MF	phospholipid binding	5	0,040
MF	phosphatidylserine binding	4	0,048

MF	transporter activity	8	0,050
MF	heparin binding	7	0,064
MF	voltage-gated calcium channel activity	4	0,067
MF	C-C chemokine receptor activity	3	0,067
MF	secondary active sulfate transmembrane transporter activity	3	0,067
MF	voltage-gated potassium channel activity	5	0,077
MF	iron ion binding	10	0,077
MF	prostaglandin-endoperoxide synthase activity	2	0,077
MF	proton-dependent oligopeptide secondary active transmembrane transporter activity	2	0,077
MF	dioxygenase activity	2	0,077
KEGG	Staphylococcus aureus infection	11	0,000
KEGG	Cell adhesion molecules (CAMs)	16	0,000
KEGG	Serotonergic synapse	13	0,000
KEGG	Calcium signaling pathway	17	0,001
KEGG	Chemokine signaling pathway	16	0,001
KEGG	Long-term depression	9	0,001
KEGG	Platelet activation	13	0,002
KEGG	Retrograde endocannabinoid signaling	11	0,002
KEGG	Asthma	6	0,003
KEGG	Rap1 signaling pathway	17	0,005
KEGG	Neuroactive ligand-receptor interaction	19	0,005
KEGG	Hematopoietic cell lineage	9	0,005
KEGG	Oxytocin signaling pathway	13	0,006
KEGG	Type I diabetes mellitus	7	0,007
KEGG	Fc gamma R-mediated phagocytosis	9	0,007
KEGG	Graft-versus-host disease	6	0,010
KEGG	Regulation of lipolysis in adipocytes	7	0,010
KEGG	Circadian entrainment	9	0,010
KEGG	ECM-receptor interaction	9	0,010
KEGG	Glutamatergic synapse	10	0,012
KEGG	Tuberculosis	14	0,012
KEGG	PI3K-Akt signaling pathway	20	0,016
KEGG	Leishmaniasis	8	0,016
KEGG	Fc epsilon RI signaling pathway	7	0,017
KEGG	Dopaminergic synapse	10	0,021
KEGG	Inflammatory mediator regulation of TRP channels	9	0,025
KEGG	Cytokine-cytokine receptor interaction	14	0,025
KEGG	Morphine addiction	8	0,029
KEGG	VEGF signaling pathway	6	0,033
KEGG	Ovarian steroidogenesis	6	0,036
KEGG	Viral myocarditis	7	0,036
KEGG	Intestinal immune network for IgA production	6	0,039
KEGG	Arachidonic acid metabolism	8	0,040
KEGG	Glycerophospholipid metabolism	8	0,042
KEGG	Rheumatoid arthritis	8	0,042
KEGG	Phosphatidylinositol signaling system	8	0,044
KEGG	Aldosterone synthesis and secretion	7	0,048

KEGG	mTOR signaling pathway	6	0,053
KEGG	Allograft rejection	5	0,061
KEGG	Natural killer cell mediated cytotoxicity	8	0,061
KEGG	Axon guidance	9	0,069
KEGG	Salivary secretion	7	0,074
KEGG	Phagosome	10	0,082
KEGG	Type II diabetes mellitus	5	0,084
KEGG	Amoebiasis	8	0,084
KEGG	Thyroid hormone synthesis	6	0,091

*Functional analysis was referred to the GO term annotation according to the biological process (BP), cellular component (CC) and molecular function (MF) classification, and the KEGG pathways in which they are involved.

Supplementary Table S13. Targeted identification of differentially accumulated metabolites in liver tissue between vitrified- transferred progeny and that naturally-conceived in F1, F2 and F3 generations.

Metabolic pathway	Metabolite name	Fold change F1	Fold change F2	Fold change F3
Glycolisis/Gluconeogenesis	glycerate-3P	0,70	**3,47**	**2,55**
	ThPP	2,56	**1,44**	**1,64**
	2-hydroxy-ethyl-ThPP	0,93	0,04	**0,86**
	ß-D-Glucose-6P	-0,22	-0,07	0,21
	glycerate-1,3P2	-0,41	**-0,64**	0,19
	glyceraldehyde-3P	**-1,58**	-1,15	-0,18
	S-Acetyl-Dihydrolipoamide-E	**0,61**	-0,07	**0,34**
	Lipoamide-E	**-0,54**	**-0,23**	-0,37
	ß-D-Fructose-1,6P2	**-0,98**	0,55	-0,53
	cAMP	**-0,61**	0,34	0,51
Citrate cycle (TCA cycle)	oxalosuccinate	**3,59**	**2,52**	**2,12**
	ThPP	2,56	**1,44**	**1,64**
	2-hydroxy-ethyl-ThPP	0,93	0,04	**0,86**
	succinate	**-0,64**	**-0,40**	0,15
	cis-aconitate	0,00	0,10	0,13
	S-Acetyl-Dihydrolipoamide-E	**0,61**	-0,07	**0,34**
	citrate	**-0,62**	**-0,82**	-0,18
	Lipoamide-E	**-0,54**	**-0,23**	-0,37
	3-carboxy-1-hydroxypropyl-ThPP	**2,65**	0,41	-1,06
Biosynthesis of amino acids	arginine	-0,07	-0,03	0,17
	asparagine	0,05	-0,04	0,30
	aspartate	-0,04	-0,04	0,31
	cysteine	**-2,38**	**-2,32**	-2,11
	glutamic_acid	0,12	0,05	0,35
	glutamine	-0,44	-0,69	-0,05
	histidine	-0,12	-0,03	0,25
	isoleucine-leucine	0,31	-0,15	0,50
	lysine	0,09	0,19	-0,01
	methionine	0,19	**-0,27**	0,39
	phenylalanine	0,03	-0,08	0,29
	proline	0,20	-0,25	0,38
	serine	0,17	**-0,66**	-1,85
	threonine	0,24	0,09	0,34
	tryptophan	**-5,51**	**-2,04**	**-1,25**
	tyrosine	0,44	-0,10	0,51
	valine	0,36	0,01	0,92
Oxidative phosphorylation	NADH	**-2,99**	**-1,33**	1,31
	fumarate	-0,22	-0,18	0,30
	gluconate	-0,73	-0,69	0,00
	succinate	**-0,64**	**-0,40**	0,15
	adenosine_diphosphate	0,47	-0,07	0,09
	FAD2+	0,03	-0,03	-0,17
	glucose	**0,95**	0,21	-0,51

Biosynthesis of unsaturated fatty acids	Adrenic_acid	**-0,65**	**0,74**	**-0,85**
	Arachidic_acid	0,32	**-0,96**	**-0,64**
	Arachidonic_acid	**-0,13**	**0,78**	**-0,59**
	Behenic_acid	0,85	0,34	-0,55
	Docosadienoic_acid	-0,39	**0,37**	-0,44
	Docosahexaenoic_acid	-0,09	0,26	-0,37
	Docosapentaenoic_acid	**-0,40**	**0,50**	**-0,78**
	Erucic_acid	-0,09	**-0,07**	**-0,27**
	Icosadienoic_acid	-0,10	**0,39**	-0,23
	Icosapentaenoic_acid	**0,10**	**0,42**	-0,34
	Icosatrienoic_acid	-0,09	**0,53**	-0,41
	Icosenoic_acid	0,00	-0,01	-0,15
	Lignoceric_acid	0,99	0,05	-0,29
	Linoleic_acid	-0,07	**0,50**	**-0,72**
	Nervonic_acid	**0,24**	**0,24**	**-0,59**
	Oleic_acid	-0,07	0,04	**-0,55**
	α-Linolenic_acid	**0,29**	0,20	**-0,62**
	Palmitic_acid	0,06	**-0,15**	**-0,36**
	Stearic_acid	**0,50**	**0,32**	**-0,24**
Arachidonic acid metabolism	11,14,15-theta	**1,16**	**-1,14**	-0,50
	hepoxilin_A3	0,32	0,21	0,36
	leukotriene_F4	0,64	-0,03	0,27
	11-dehydro_Thromboxane_B2	0,53	0,15	0,23
	6-Ketoprostaglandin_E1	-0,08	-0,16	0,18
	Trioxilin_A3	0,05	0,12	0,20
	5,6-Dihydroxy-8Z,11Z,14Z-eicosatrienoic_acid	**1,18**	**-1,08**	0,06
	6-Ketoprostaglandin_E1	**0,78**	**-0,47**	-0,08
	Tetrahydro-3,4-furandiol	0,13	0,09	-0,29
	prostaglandin_B2	**-0,80**	**-0,84**	-0,33
	11,14,15-theta	**1,16**	**-1,14**	-0,50
	15-Keto-prostaglandin_F2alpha	**-0,74**	**-1,03**	-0,37
	5-Oxo-ETE	**0,66**	-0,38	-0,47
	5-HETE	**0,36**	-0,07	**-0,50**
	11(12)oxido-5,8,14-eicosatrienoic_acid	**0,43**	-0,12	**-0,52**
	14,15-dihydroxy-5,-8,11-eicosatrienoic_acid	0,35	0,01	**-0,72**
	5(6)oxido-8,11,14-eicosatrienoic_acid	0,16	0,38	**-0,96**
	Lecithins	0,18	-0,68	**-1,25**
	11,12,19-/11,12,20-trihydroxy-5,8,14-eicosatrienoic	**1,24**	**-1,21**	**-0,66**
	14,15,19-/14,15,20-trihydroxy-5,8,11-eicosatrienoic	**1,05**	**-0,55**	-0,36
	Prostacyclin	**0,87**	**-1,11**	-0,50
Phosphatidylcholines	PC_(36:5)	**-0,57**	0,31	**-1,00**
	PC_(38:7)	0,22	0,04	-0,26
	PC_(34:4)	**-0,76**	0,61	**-0,81**
	PC_(30:3)	-0,27	0,02	**-0,77**
	PC_(40:6)	**0,58**	**-0,64**	-0,27
	PC_(38:6)	-0,25	0,25	-0,47

	PC_(36:4)	**-0,58**	0,38	**-0,83**
	PC_(30:0)	**-0,49**	-0,02	**-0,96**
	PC_(34:1)	-0,45	0,01	-0,51
	PC_(34:2)	**-0,43**	0,03	**-0,70**
	PC_(38:5)	**-0,42**	0,33	**-0,69**
	PC_(36:3)	**-0,46**	0,27	**-0,71**
	PC_(38:4)	**-0,49**	0,58	**-0,74**
	PC_(34:0)	-0,32	-0,54	-0,61
	PC_(32:1)	-0,46	0,08	**-0,79**
	PC_(36:1)	-0,13	-0,11	**-0,86**
	PC_(32:0)	**-0,54**	-0,37	-0,43
	PC_(34:6)	-0,55	-0,63	**-0,87**
	PC_(36:2)	-0,10	-0,13	**-0,92**
Sphingolipid metabolism	SM_(18:0)	-0,21	0,21	**-0,80**
	SM_(d34:0)	0,05	-0,24	-0,54
	SM_(16:0)	-0,19	-0,32	**-0,71**
Cholesterol metabolism	cholesterol	**-0,20**	-0,07	-0,17
	cholesterol_alpha-epoxide	0,04	-0,03	**-0,48**
Glycerolipid metabolism	TAG_(50:1)	**1,71**	**0,46**	**-1,84**
	TAG_(50:4)	**1,40**	-0,58	**-1,95**
	TAG_(54:5)	**0,28**	**1,57**	**-1,02**
	TAG_(54:8)	**0,81**	**1,38**	**-1,56**
	TAG_(52:1)	**1,59**	**0,43**	**-1,13**
	TAG_(52:2)	**1,10**	**0,68**	**-1,07**
	TAG_(54:9)	**2,01**	**-0,70**	**-1,34**
	TAG_(50:0)	**1,83**	**1,07**	**-1,35**

Red denotes statistical differences at $p<0.05$.

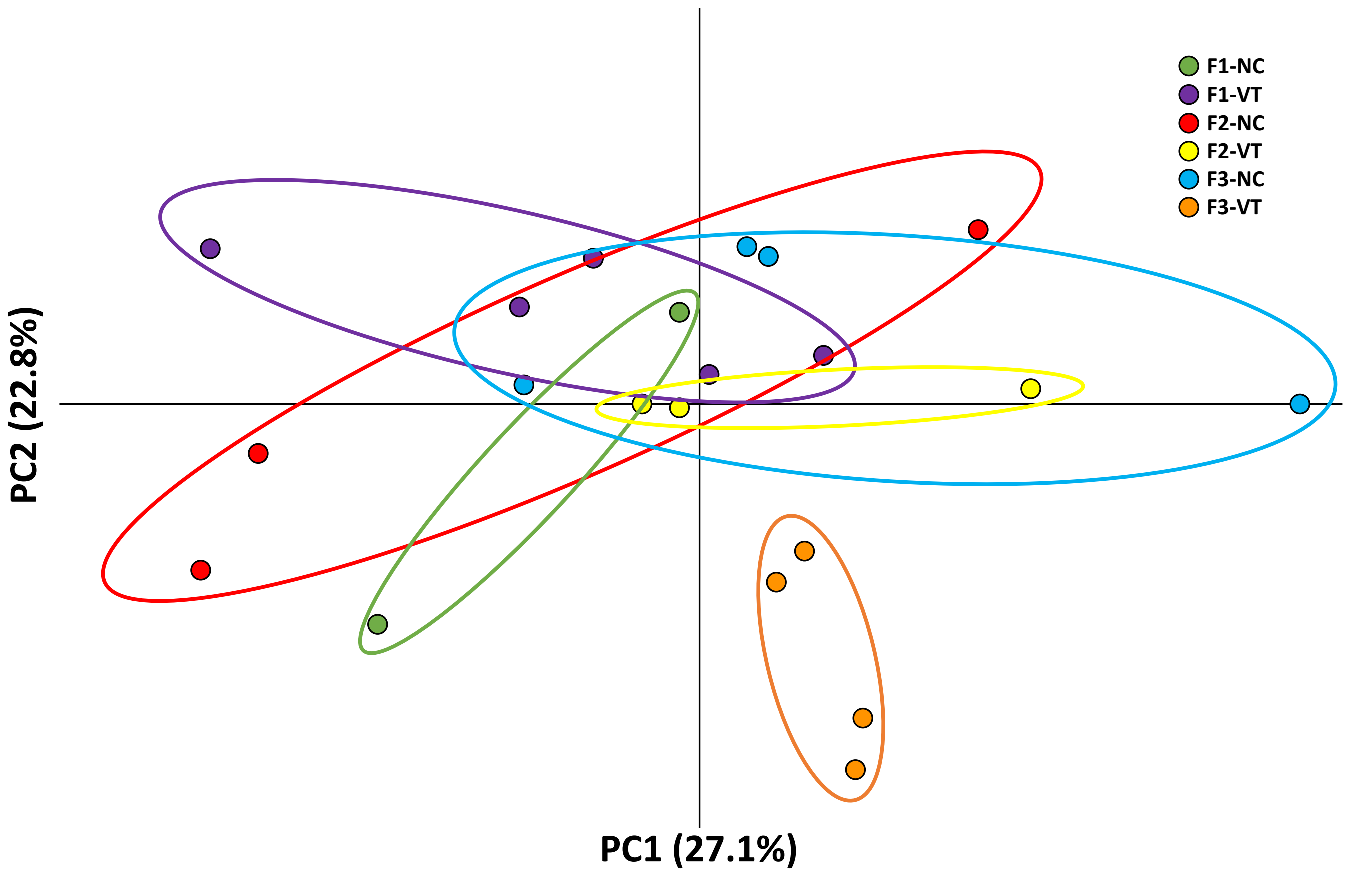

Supplementary Fig. S1. Principal component analysis, assessing the expression profile variability of each sample in each generation. Adjusted color ellipses indicate the dispersion of the samples by experimental group (NC:naturally-conceived; VT:vitrified-transferred) and generation (F1, F2 and F3).
